# Population nutrikinetics of green tea extract

**DOI:** 10.1371/journal.pone.0193074

**Published:** 2018-02-21

**Authors:** Catharina Scholl, Anna Lepper, Thorsten Lehr, Nina Hanke, Katharina Luise Schneider, Jürgen Brockmöller, Thomas Seufferlein, Julia Carolin Stingl

**Affiliations:** 1 Research Division, Federal Institute of Drugs and Medical Devices (BfArM), Bonn, Germany; 2 Institute of Pharmacology of Natural Products and Clinical Pharmacology, University of Ulm, Ulm, Germany; 3 Clinical Pharmacy, Department of Pharmacy, Saarland University, Saarbrücken, Germany; 4 Institute for Clinical Pharmacology, University Medical Center Göttingen, Georg-August University, Göttingen, Germany; 5 Department of Internal Medicine I, University of Ulm, Ulm, Germany; Weill Cornell Medical College Qatar, QATAR

## Abstract

Green tea polyphenols may contribute to the prevention of cancer and other diseases. To learn more about the pharmacokinetics and interindividual variation of green tea polyphenols after oral intake in humans we performed a population nutrikinetic study of standardized green tea extract. 84 healthy participants took green tea extract capsules standardized to 150 mg epigallocatechin-gallate (EGCG) twice a day for 5 days. On day 5 catechin plasma concentrations were analyzed using non-compartmental and population pharmacokinetic methods. A strong between-subject variability in catechin pharmacokinetics was found with maximum plasma concentrations varying more than 6-fold. The AUCs of EGCG, EGC and ECG were 877.9 (360.8–1576.5), 35.1 (8.0–87.4), and 183.6 (55.5–364.6) h*μg/L respectively, and the elimination half lives were 2.6 (1.8–3.8), 3.9 (0.9–10.7) and 1.8 (0.8–2.9) h, respectively. Genetic polymorphisms in genes of the drug transporters MRP2 and OATP1B1 could at least partly explain the high variability in pharmacokinetic parameters. The observed variability in catechin plasma levels might contribute to interindividual variation in benefical and adverse effects of green tea polyphenols. Our data could help to gain a better understanding of the causes of variability of green tea effects and to improve the design of studies on the effects of green tea polyphenols in different health conditions.

**Trial registration:** ClinicalTrials.gov: NCT01360320

## Introduction

Green tea prepared from the dried leaves of the plant *Camellia Sinensis* (L.) Kuntze is one of the most widely consumed bevarages in the world. Tea leaves contain up to 30% phenolic substances, commonly known as tea catechins. The major catechins in green tea are (-)-epigallocatechin-3-gallate (EGCG), (-)-epigallocatechin (EGC), (-)-epicatechin-3-gallate (ECG) and (-)-epicatechin (EC) [1]. Epidemiological studies showed a reduction of the cancer risk and a protective effect on the cardiovascular system with green tea consumption [2–4]. These effects seem to be governed by green tea catechins, particularly EGCG [3,5]. Due to these presumed beneficial effects green tea polyphenols are of growing interest for cancer prevention. Several observational and interventional studies have been conducted investigating these chemopreventive effects (reviewed in [2–4,6,7]).

The oral bioavailability of EGCG and other green tea catechins which is important for its systemic and chemopreventive effects has been investigated in humans in several studies administering purified EGCG [8–12], tea [13–16] or green tea extract [10–12,17–19]. EGCG, EGC, ECG, EC are absorbed after oral administration with t_max_ varying from one to five hours. The oral bioavailability is suggested to be low in humans [17] as it was earlier demonstrated in rodents [20,21]. The plasma concentrations of EGCG were proportional to the dose administered but showed high interindividual variability in a study including 10 volunteers [9]. No significant differences in nutrikinetic parameters of purified EGCG and green tea extract were observed [10,11]. In studies investigating daily dosing of EGCG or green tea extract after overnight fast for up to two weeks almost no accumulation of catechins in the blood plasma was detected [8,11,19].

Green tea catechins are absorbed in the small intestine [22] and biotransformed in the liver and in enterocytes in the small intestine mostly by phase II metabolizing enzymes leading to methylated, sulfated and glucuronidated metabolites [23]. Catechins passing the small intestine either unabsorbed or after enterohepatic recycling are then in substantial amounts broken down to ring-fission metabolites most propably by colonic bacteria [24,25]. Methylation of green tea catechins is catalyzed by catechol-O-methyltransferase (COMT) [23,26]. In several studies it was shown that EGCG is methylated by COMT, forming 4’-O-methyl-EGCG, 4”-O-methyl-EGCG and 4’-4”-di-O-methyl-EGCG metabolites [26,27]. EGCG was shown to inhibit COMT activity *in vitro* [27,28], but a recent study in healthy volunteers consuming a single dose (750 mg) of EGCG after one night fasting demonstrated that COMT activity measured in erythrocytes was not inhibited [29]. Green tea catechins are extensively sulfated or glucuronidated. In plasma samples more than 80% of polyphenols have been found to be conjugated after ingestion of green tea extract. EGCG and EC were detected mainly in sulfate forms, whereas for EGC the glucuronide forms dominated in the plasma samples [30]. Sulfotransferases (SULT) contribute in sulfate conjugation of catechins, but it is still not clear which isoforms are exactly involved. EC is sulfated through SULT1A1 in human liver cytosol [31], but EGCG appears to be a substrate for SULT2A1 but not SULT1A1 [32]. SULT1A1 is the main isoform in the human liver and SULT1A1 and SULT1A3 are highly expressed in the intestine. EGCG and EGC are inhibitors of a number of SULT isoforms including SULT1A1 and SULT1A3 [33]. Glucuronidation of EGCG and EGC is catalyzed predominantely by UDP-glucurinosyltransferases (UGT) 1A1, 1A8 and 1A9. The intestinal specific UGT1A8 isoform has been shown to have the highest catalytic efficiency of these UGTs [23,34].

Bioavailability of green tea catechins may not only depend on biotransformation processes but also on uptake and excretion. Multidrug resistance-associated protein (MRP) 1, MRP2 and p-glycoprotein (Pgp) have been shown to be involved in the excretion of green tea catechins. *In vitro* studies showed that EGCG is a substrate for MRP1 and MRP2 but not for Pgp [35] and that ECG and EC may be substrates of MRP2 [36]. In addition, several studies suggest that green tea catechins inhibit Pgp as well as organic anion-transporting polypeptide (OATP)1B1, OATP1B3, organic cation transporter (OCT)1, OCT2, multidrug and toxin extrusion (MATE)1 and MATE2-K transporters [37,38].

Several inherited polymorphisms in drug metabolizing enzymes and transporters have been reported to influence the pharmacokinetic and disposition of drugs. But there is not much knowledge and evidence if functional polymorphisms in drug metabolizing enzymes and drug transporters affect the pharmacokinetic of green tea catechins.

A detailed characterization of interindividual variation in plasma concentrations of green tea catechins is important to interpret both beneficial but also adverse systemic effects. Blood concentrations of green tea polyphenols are relevant for cardiovascular and metabolic effects. Together with unabsorbed catechins having local effects, systemic concentrations of the polyphenols may also be relevant for the anti-cancer effects in the colon. To better understand the systemic exposure with polyphenols for the outcomes of clinical trials on health preventive effects of green tea polyphenols, the objectives of the present study were to characterize the nutrikinetics of EGCG, EGC and ECG in a large and comprehensive study in healthy volunteers, using the standardized green tea extract capsules and to investigate which factors have a relevant impact on that systemic exposure and the large interindividual variation in catechin plasma concentrations.

## Materials and methods

The protocols for this trial (S1–S3 Files) and supporting TREND checklist (S4 File) are available as supporting information.

### Study drug

Green tea extract capsules were produced by Dr. Loges (Hamburg, Germany) using a commercially available green tea extract. Each capsule contained approximately 225 mg total catechins. The catechin content of the capsules was standardized to 150 mg EGCG, containing in addition 18 mg EGC, 35 mg ECG, 10 mg EC and 1.3 mg caffeine.

### Participants

**Fig 1:**
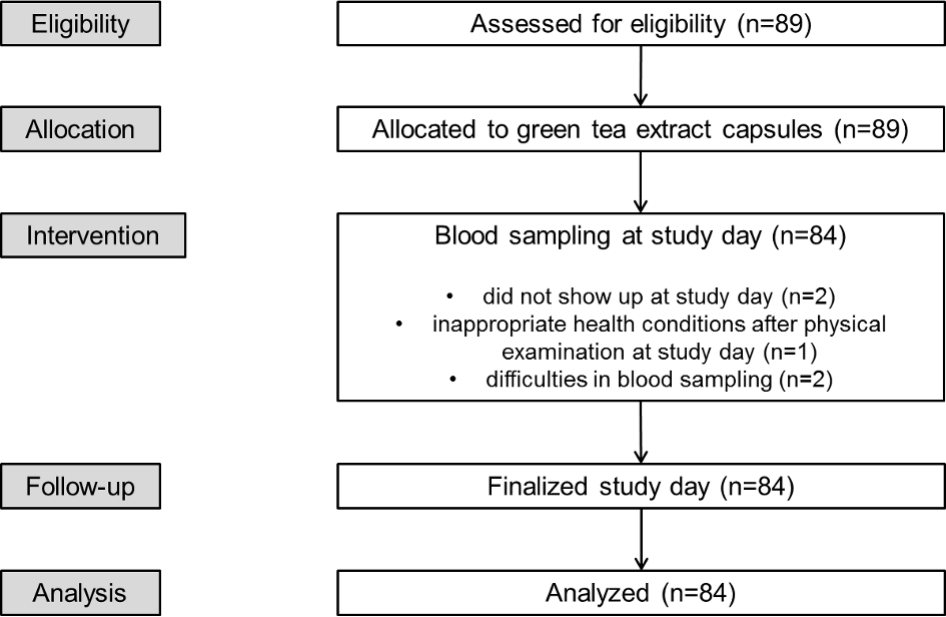
Consort flowchart.

89 healthy volunteers with Western European ancestry were enrolled in the study between 1^st^ November 2011 and 30^th^ June 2012. Participants were approached with a notice at the local university notice-board and interested volunteers were recruited. Before the start of the study participants were physically examined and a blood sample was taken to evaluate the health condition of the participant. During this check-up the following demographic and diagnostic parameters were collected: gender, age, weight, height, BMI, typical clinical biochemistry parameters (AST, ALT, Bilirubin), complete blood count, green tea consumption habits, smoking status, frequency of alcohol consumption and use of oral contraceptives. Exclusion criteria for the participation in the study were pregnancy, any acute or chronic disease and regular use of drugs with the exception of oral contraceptives. Five participants dropped out of the study (dropout rate: 5.6%) due to inappropriate health conditions after physical examination, difficulties in blood sampling, or did not show up at study day (Fig 1). All participants had a minimum age of 18 years and a body mass index (BMI) between 18 and 30 kg/m^2^. The study protocol was approved by the Ethical Committee of the University Ulm (permission: 171/11); participants gave their written informed consent after detailed explanation of the study. Sample size was estimated a priori based on AUC values published previously [8]. Assuming a frequency of 20% of carriers of the methionine variant (heterozygote plus homozygote carrier) and an allele frequency of 11% for the COMT polymorphism an effect on the pharmacokinetics would be detectable in n = 100 participants. If n = 20 participants carry either Val/Met or Met/Met genotypes and n = 80 carry Val/Val genotype a difference of less than 30% in the oral clearance of EGCG could be detected with a power of 80% and a significance of 0.05. After finally including 84 participants in the analysis a subsequent sample size estimation showed that a group sample sizes of 67 and 17 achieve 80% power to detect a difference of 25.8% with a significance level (alpha) of 0.05 using a two-sided two-sample equal-variance t-test.

### Study design

The study was an open one-arm nutrikinetic study conducted at the university Ulm. It was aimed to measure the plasma concentrations of green tea catechins in a cohort of healthy individual. To capture a possible influence of a continuous intake of catechins, e.g. accumulation of catechins, transcriptional changes or inhibition of membrane transporters and metabolizing enzymes caused by catechins, plasma concentrations were determined after 5 days of intake of green tea extract.

**Fig 2:**
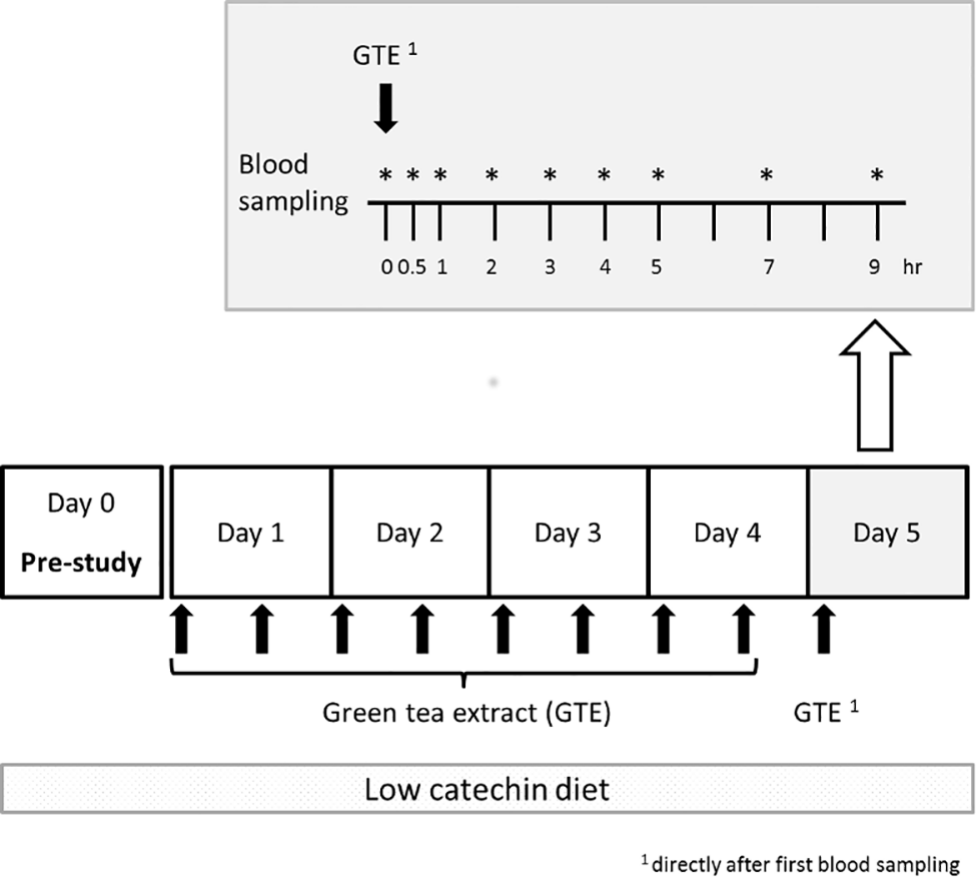
Schematic representation of the study design and blood sampling. The experiment includes one pre-study day and 5 days treatment with standardized green tea extract (GTE). Blood sampling for nutrikinetics takes place on day 5. Dosing of GTE: day 1–4: twice a day (≙ 2 x 150 mg EGCG/day), day 5: after overnight fasting intake of one GTE capsule (150 mg EGCG).

The study was conducted as briefly described. One day before the treatment period participants were requested to stop any green tea consumption and to avoid polyphenol rich nutrition during study. All participants were asked if they did extensive physical exercises in the days before and during the study. None of the participants stated an extensive physical activity. For the first 4 days of the treatment period participants took green tea capsules twice daily (≙ 2 x 150 mg EGCG/day). Capsules were swallowed in the morning and in the evening (12 h interval) with a glass of tap water during meal. After one night fasting, on the morning of day 5 a blood sample for the baseline measurements was taken. Afterwards another capsule of green tea extract (150 mg EGCG) was administered and subsequently venous blood samples were taken after 0.5, 1, 2, 3, 4, 5, 7, and 9 hours (Fig 2). A standardized meal and snack were served 4 h and 10 h after the intake of the green tea extract capsule.

### Tea polyphenol concentration measurement

Venous blood was drawn into ethylene diamine tetraacetic acid (EDTA) containing tubes and centrifuged within 10 minutes after sampling at 3500 rpm for 10 minutes. Then, 1 mL aliquots of plasma were mixed with 20 μL of antioxidant solution (2 g ascorbic acid per 10 mL double distilled water including 70 μL of 0.5 M EDTA). Plasma samples were frozen immediately after mixing with the antioxidant solution and stored at -80°C until analysis.

Calibrators for EGCG, EGC, ECG, and EC were obtained from Sigma-Aldrich. As internal standard (IS) deuterated EGCG (Epigallocatechingallate-d4-d6) from Synfine (Richmond Hill, Canada) was used and the peak areas of all analytes were normalized to the area of the deuterated (d4) isotope of EGCG. Calibrator and IS stock solutions were prepared as 1 mg/mL solutions in 50% methanol including 2% of the antioxidant solution given above. Calibrators and IS for measurement were prepared in bovine serum with the same proportion of antioxidant solution as used for the human plasma samples to be analyzed.

Quantification was performed after precipitation and online solid phase extraction using reversed phase HPLC with tandem mass spectrometric detection. HPLC-MS/MS-system with column switching was used as essentially described earlier [39]. The HPLC-MS/MS-system consisted of a vantage mass spectrometer with a HESI-II Ionization Probe (Thermo Separations, Waltham, MA, USA). The liquid chromatography unit consisted of a P680-pump, a HPG3400, a WTS 3000 and a column compartment with column switching valves (Dionex, Germering, Germany). The precipitation reagent stock solution was prepared from 350 mL methanol, 150 mL 0.3 M ZnSO_4_ and 9.375 mL acetic acid. For precipitation 200 μL of plasma to be analyzed and 400 μL of precipitation reagent (45 mL precipitation reagent stock solution, 0.9 mL antioxidant solution, 112.5 mL internal standard) were mixed for 15 minutes and then centrifuged at 4500 rpm for 15 minutes. 4 x 95 μL sample supernatant was injected using a multiple injection mode onto the extraction column 3.0 x 10 mm 3 μm Aqua Perfect (MZ-Analysentechnik Mainz Germany) and washed with 3 mL/min 1% acidic acid. After column switching, the analytes were eluted onto an analytical column (BEH Shield RP18 1.7 μm 2.1 x 100 μm, Waters Milford USA). Electrospray MS/MS detection was used with ionization in the positive mode using the following transitions: EGCG: 459/139, EGCG-d4-d6: 463/141, EGC: 307/139, ECG: 443/123 and EC: 291/139. Integration and calculation of the ratios of analyte/IS peak areas was performed using the QuanLynx software. Interassay coefficient of variation over all series was 12.6%, 13.8% and 8.9% for the 5 ng/mL, 50 ng/mL and 500 ng/mL controls, respectively.

### DNA isolation and genotyping of *ABCC2*, *SLCO1B1*, *ABCB1*, *COMT*, *SULT1A1* and *UGT1A1*

DNA was extracted from EDTA blood using QIAamp DNA Mini Kit (Qiagen, Hilden) following the manufacturer’s instruction. Following genotypes for MRP2 (*ABCC2)*: c.1249G>A (rs2273697), c.3972C>T (rs3740066), c.-24C>T (rs717620), c.4544G>A (rs8187710), *OATP1B1 (SLCO1B1)*: c.521T>C (rs4149056), c.388A>G (rs2306283), c.463C>A (rs11045819), Pgp (*ABCB1)*: c.3435T>C (rs1045642), c.1236T>C (rs1128503), c.2677T>G (rs2032582), *COMT* c.1947G>A (rs4680) *SULT1A1* c.-197G>A (rs750155) and UGT1A1*28: (TA)7 repeat (rs8175347) were detemined using PCR amplification with real-time PCR probes. In brief, 5 μL DNA (10 ng/μL) was amplified using Lightcycler® FastStart DNA Master HybProbe Mix (Roche, Germany) and the respective LightSNiP Assay (TIBMolBiol, Berlin). The following PCR protocol was applied: 10 min at 95°C, followed by 45 cycles for amplification: 10 s at 95°C, 10 s at 60°C, 15 s at 72°C. Melting curve analysis was performed to distinguish between the different genotypes.

### Pharmacokinetic analysis

Non-compartmental analysis (NCA) was performed using Phoenix WinNonlin 6.4 (Certara USA Inc., Princeton, NJ, USA). The linear trapezoidal-linear interpolation method was applied to calculate the areas under the plasma concentration-time curves (AUCs). Computation formulas used in the non-compartmental analysis are given in the modeling supplement (S5 File). Correlation analyses were performed on AUCs, volumes of distribution and clearances of the three measured catechins.

The population pharmacokinetic (PopPK) model development for the separate characterization of EGCG, EGC and ECG nutrikinetics was performed using non-linear mixed-effects modeling techniques (NONMEM version 7.3, ICON Development Solutions, Ellicott City, MD, USA), that allow estimation of population medians for model parameters with simultaneous quantification of interindividual variability (IIV) and residual (unexplained) variability. The first-order conditional estimation algorithm in NONMEM with interaction option was applied and IIV was modeled using exponential random effects models. An example of an exponential model is Pki = Θk·eηki where Pki denotes the value of the parameter k of the individual i, Θk is the typical value of the population parameter k and ηki is the difference between the natural logarithms Pki and Θk. It is assumed that all Pki are log-normally distributed. In addition, all ηki are assumed to be independently, multivariately symmetrically distributed with zero mean and the variance ω^2^k. The use of an exponential model has several attractive features. It ensures that all parameters are strictly positive avoiding the estimation of negative non-physiological individual values. Also ω^2^k becomes dimensionless and expresses approximately the coefficient of variation in the model parameters [40]. Model selection was based on several analyses, including the objective function value (OFV) provided by NONMEM, precision of parameter estimates and visual inspection of goodness-of-fit plots. One nested model was considered superior to another when the OFV was reduced by 3.84 points or more (Chi-square, p<0.05, 1 degree of freedom).

For internal model evaluation, a visual predictive check (VPC) was performed based on 1000 simulations using the final PK models with their fixed- and random-effects parameters. Median simulated plasma concentrations and corresponding 5^th^ and 95^th^ percentiles were plotted against time, and overlaid with the observed data. SAS version 9.4 (SAS Institute Inc., Cary, NC, USA) was used for statistical analyses (characterization of the study population, analysis of frequency distributions and correlations between different PK parameters) and generation of graphics.

Furthermore a large panel of covariates was tested, including gender, age, weight, height, BMI, green tea consumption habits, smoking status, frequency of alcohol consumption, use of oral contraceptives, genotypes of drug transporters and phase II enzymes suggested to be involved in catechin transport and biotransformation and multiple blood count parameters. A stepwise covariate model-building strategy was employed using forward inclusion of covariates followed by backward elimination. For the forward inclusion, covariates were added to the model in the order of their ranking (decrease of OFV when included individually compared to the base model), covariates were retained in the model if their inclusion improved the OFV significantly (p≤0.01). For the backward elimination, included covariates were removed from the model (the covariate causing the smallest increase of OFV when removed individually is eliminated first), covariates were retained in the model if their removal worsened the OFV significantly (p≤0.001). Following the first covariate testing, the significance requirement for covariate inclusion and retention of the different analysed transporter and phase II enzyme genotypes were lowered to p≤0.025 for both, forward inclusion and backward elimination, in an explorative approach to generate hypotheses to explain the high interindividual differences found in the observed pharmaocokinetic profiles.

The final EGCG, EGC and ECG models were used for prediction of single dose and steady-state plasma concentration time profiles as well as AUC, maximum plasma concentration (C_max_) and minimum plasma concentration (C_trough_) values for the assessment of green tea catechin accumulation in the steady-state during a twice daily dosing regimen.

## Results

Plasma concentration time curves of green tea catechins at the 5^th^ day of twice daily oral intake of standardized green tea extract capsules were assessed in 84 healthy volunteers. Details on the characteristics of the study participants are given in Table 1.

**Table 1:** General characteristics of the study population.

	Total	Male	Female
Number of participants	84 (89)	30	54
Age (mean) [years]	24.5 (SD 3.9)	24.7 (SD 2.5)	24.4 (SD 4.5)
Age (range) [years]	19–49	21–32	19–49
Weight [kg]	68.0 (SD 11.7)	80.2 (SD 9.2)	61.3 (SD 6.3)
Height [m]	1.7 (SD 0.1)	1.8 (SD 0.1)	1.7 (SD 0.1)
BMI [kg/m^2^]	22.7 (SD 2.6)	24.1 (SD 2.8)	21.9 (SD 2.1)
**Green tea consumption**			
Yes	10	4	6
No	74	26	48
**Smoking status**			
Never	56	17	39
Former	8	3	5
Current	20	10	10
**Alcohol consumption**			
Yes	75	28	47
No	9	2	7
**Contraceptives**			
Yes	37	0	37
No	47	30	17
**Clinical Covariates**			
AST [U/L]	24.3 (SD 7.5)	28.5 (SD 10.4)	22.0 (SD 5.2)
ALT [U/L]	23.8 (SD 10.4)	29.8 (SD 12.2)	20.5 (SD 7.4)
Bilirubin [μmol/L]	10.6 (SD 6.5)	15.3 (SD 7.6)	8.1 (SD 4.1)
Leucocytes [Giga/L]	6.5 (SD 1.8)	6.2 (SD 1.8)	6.6 (SD 1.8)
Erythrocytes [Tera/L]	4.6 (SD 0.5)	5.1 (SD 0.4)	4.3 (SD 0.3)
Hemoglobin [g/dL]	14.0 (SD 1.5)	15.5 (SD 1.1)	13.1 (SD 0.8)
Hematocrit	0.4 (SD 0.04)	0.5 (SD 0.03)	0.4 (SD 0.02)
Thrombocytes [Giga/L]	236.8 (SD 53.0)	228.9 (SD 41.3)	241.1 (SD 58.0)

SD: standard deviation

### Concentration-time profiles and non-compartmental pharmacokinetic analysis

**Fig 3:**
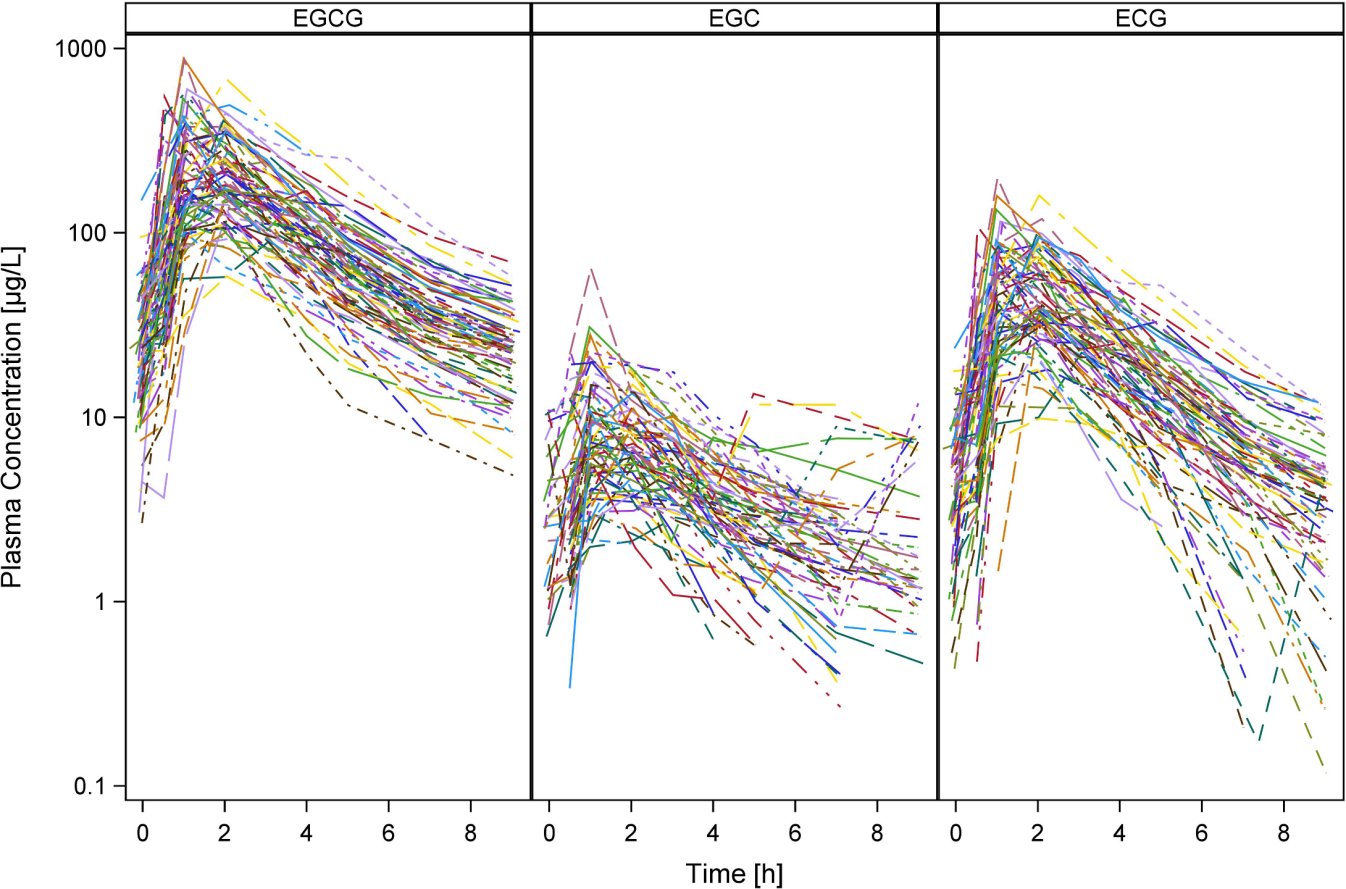
Individual measured plasma concentration-time profiles of EGCG, EGC and ECG on the 5^th^ day of oral administration.

Individual plasma concentration-time profiles of EGCG, EGC and ECG at the 5^th^ day of oral administration are presented in Fig 3. Plasma levels of EC were below detection limit. Basal plasma concentrations for EGCG, EGC and ECG were low but variable between individuals with mean values of 26.8 μg/L (limit of detection (LoD)– 149.9 μg/L), 3.8 μg/L (LoD– 10.9 μg/L) and 4.5 μg/L (LoD– 23.6 μg/L), respectively. Peak plasma concentrations (C_max_), area under the curve (AUC_0-end_) and time to reach C_max_ (t_max_) from the non-compartmental analysis are presented in Table 2. Drug concentrations declined in a biexponential fashion with terminal half-lives of 2.6 (1.8–3.8), 3.9 (0.9–10.7) and 1.8 (0.8–2.9) h for EGCG, EGC and ECG, respectively.

**Table 2:** Pharmacokinetic parameters and their variability in the study population (5th - 95th percentile), non-compartmental analysis.

	EGCG	EGC	ECG
C_max_ [μg/L]	275.4 (95.1–577.0)	10.7 (3.0–22.6)	60.1 (18.1–118.9)
t_max_ [h]	1.6 (1.0–2.1)	1.5 (0.5–3.1)	1.7 (1.0–2.2)
AUC_0-end_ [h*μg/L]	877.9 (360.8–1576.5)	35.1 (8.0–87.4)	183.6 (55.5–364.6)
t_1/2_ [h]	2.6 (1.8–3.8)	3.9 (0.9–10.7)	1.8 (0.8–2.9)
V/F [L]	717.1 (241.3–1636.2)	2410.0 (437.8–5619.1)	548.8 (224.6–1179.5)
CL/F [L/h]	187.0 (77.5–362.6)	523.4 (163.3–1267.3)	236.1 (91.5–566.8)

V: volume of distribution, F: bioavailability, CL: total body clearance

**Fig 4:**
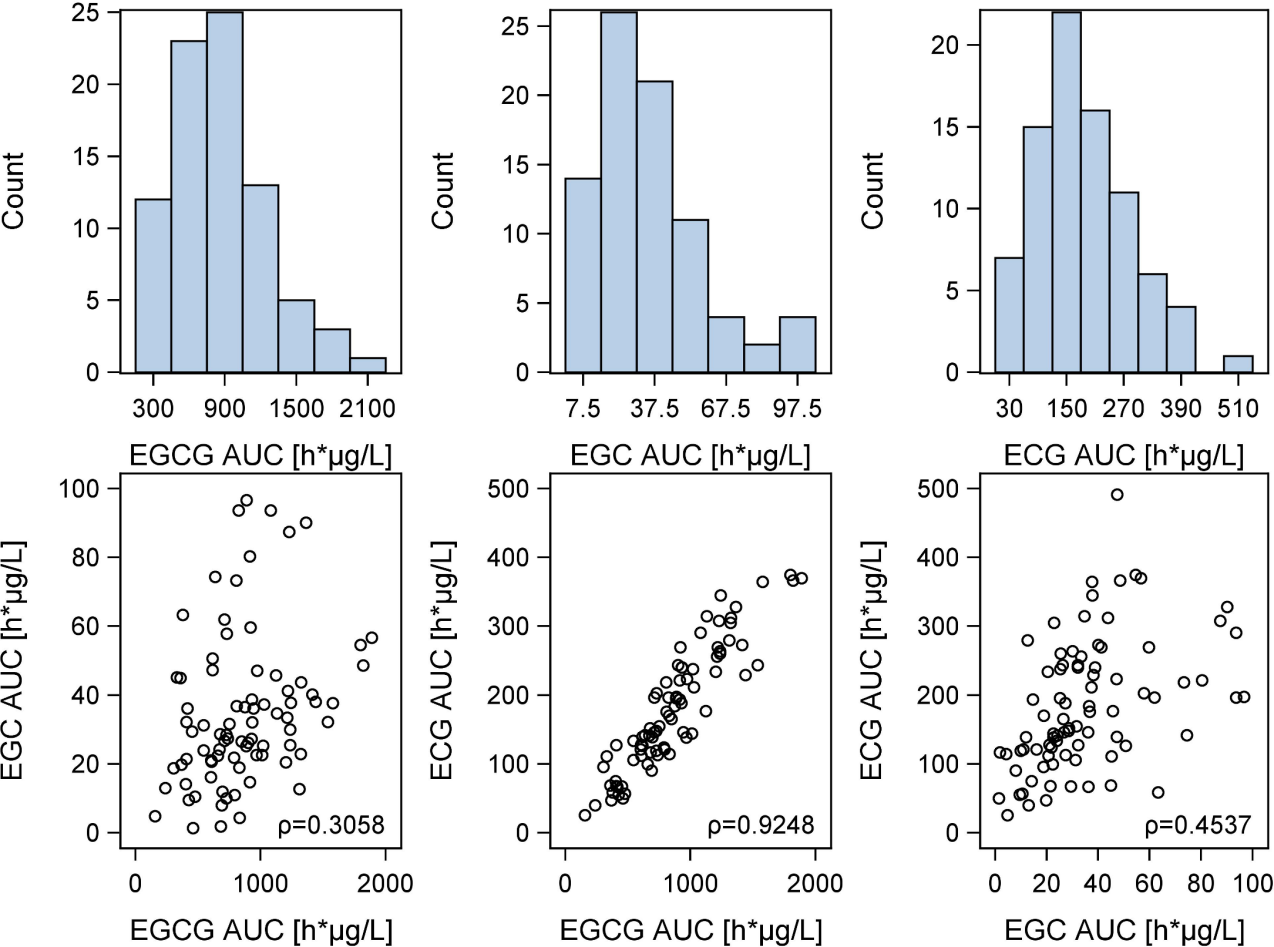
Correlation between the AUCs of the different green tea catechins. Distribution histograms of the AUCs and scatter plots showing the correlation between the AUCs of the different green tea catechins. ρ: Pearson correlation coefficients: AUC_EGCG_−AUC_EGC_: 0.3058 (p = 0.0052); AUC_EGCG_−AUC_ECG_: 0.9248 (p<0.0001), AUC_EGC_−AUC_ECG_: 0.4537 (p<0.0001).

AUC values, volumes of distribution (V/F) and total body clearances (CL/F) of EGCG, EGC and ECG were significantly correlated. Scatter Plots showing the relation of AUCs of the different catechins are presented in Fig 4.

### Compartmental pharmacokinetic analysis / PopPK model development

Concentration-time profiles of the three catechins were best described by two-compartment models with one central and one peripheral compartment. Absorption of EGCG and ECG were modeled by a zero-order dissolution into the depot compartment (constant amount dissolved per time, concentration independent) combined with a first-order absorption processes from the depot (absorption rate is concentration dependent) with a lag time of 19 minutes. In contrast, absorption of EGC was best described by a first-order absorption process only, with a lag time of 25 minutes.

**Fig 5:**
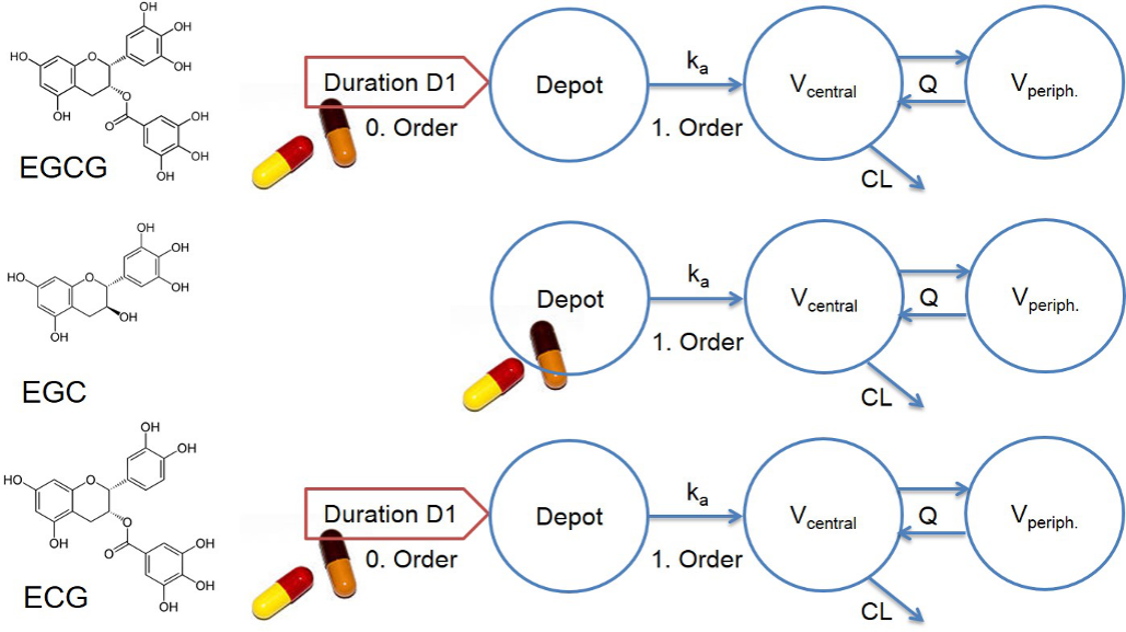
PopPK models. Structures of the three independently developed PopPK models employing two-compartment nested models with different descriptions of the absorption processes for the gallate (EGCG, ECG) and non-gallate (EGC) green tea catechins. The depot compartment (Depot) represents the gastrointestinal tract, from where the catechins are absorbed into the central volume of distribution (V_central_), representing the blood and quickly equilibrating tissues such as liver and kidney. V_periph._: peripheral volume of distribution, D1: dissolution duration, ka: absorption time constant, Q: intercompartmental clearance, CL: clearance from V_central_.

Distribution and elimination of EGCG, EGC and ECG were characterized by a central and a peripheral volume of distribution with an intercompartment clearance (Q/F) and a systemic first-order clearance from the central volume of distribution (CL/F). A schematic representation of the three structural models describing absorption, distribution and elimination of the green tea catechins is shown in Fig 5. The model equations are given in the modeling supplement (S5 File). The PopPK model parameter estimates are presented in Table 3. All structural model parameters could be precisely estimated with relative standard errors ≤32% (Table 3). Large interindividual variabilities on the dissolution and absorption processes of EGCG and ECG (D1 and ka, Table 3) on the one hand, and on the central volume of distribution and intercompartmental clearance of EGC (V_central_/F and Q/F, Table 3) on the other hand were identified, indicating wide variation in absorption and transport through barriers within the human body.

**Table 3:** Pharmacokinetic parameters, estimated with the final two-compartment models.

		EGCG		EGC		ECG	
Model parameter	Unit	Estimate	RSE [%]	Estimate	RSE [%]	Estimate	RSE [%]
***Fixed effects***							
D1	[h]	0.301	11	-	-	0.422	12
Lag time	[h]	0.327	5	0.413	5	0.319	9
ka	[1/h]	1.86	11	0.830	8	1.78	13
V_central_/F	[L]	334	7	1180	31	395	7
Q/F	[L/h]	59.7	11	324	23	56.2	11
V_peripheral_/F	[L]	1260	17	10100	32	1300	18
CL/F	[L/h]	148	7	560	16	196	6
F	-	1.00*	-	1.00*	-	1.00*	-
OATP1B1_rs4149056	-	-0.152	32	-	-	-	-
MRP2_rs717620_EGCG	-	0.740	10	-	-	-	-
CONCEP	-	-	-	0.450	24	-	-
COMT_rs4680	-	-	-	0.760	15	-	-
UGT1A1*28	-	-	-	0.744	15	-	-
OATP1B1_rs2306283	-	-	-	0.653	18	-	-
MRP2_rs3740066	-	-	-	2.33	26	-	-
MRP2_rs717620_EGC	-	-	-	-0.370	27	-	-
***Random effects*: *interindividual variability***							
IIV D1	[%CV]	103	8	-	-	70	10
IIV Lag time	[%CV]	32	20	11	25	43	16
IIV F	[%CV]	46	9	37	18	57	9
IIV ka	[%CV]	79	14	-	-	78	14
IIV V_central_/F	[%CV]	-	-	80	17	-	-
IIV Q/F	[%CV]	-	-	92	14	-	-
IIV CL/F	[%CV]	25	15	38	26	39	16
***Random effects*: *residual variability***							
Prop. residual variability	[%]	17	9	28	8	18	9
Add. residual variability	[μg/L]	5	18	-	-	1	29

* Parameter fixed

Add.: additive, CL: clearance from central compartment, COMT_rs4680: effect of SNP on F, CONCEP: effect of contraceptives on V_central_/F, CV: coefficient of variation, D1: dissolution duration, F: relative bioavailability, IIV: interindividual variability, ka: absorption time constant, Lag time: delay in absorption, MRP2_rs3740066: effect of SNP on Q/F, MRP2_rs717620_EGC: effect of SNP on V_central_/F, MRP2_rs717620_EGCG: effect of SNP on F, OATP1B1_rs2306283: effect of SNP on CL/F, OATP1B1_rs4149056: effect of SNP on CL/F, Prop.: proportional, Q: intercompartmental clearance, RSE: relative standard error, UGT1A1*28: effect of SNP on CL/F, V: volume of distribution. Mathematical implementation of covariates is detailed in the modeling supplement.

**Fig 6:**
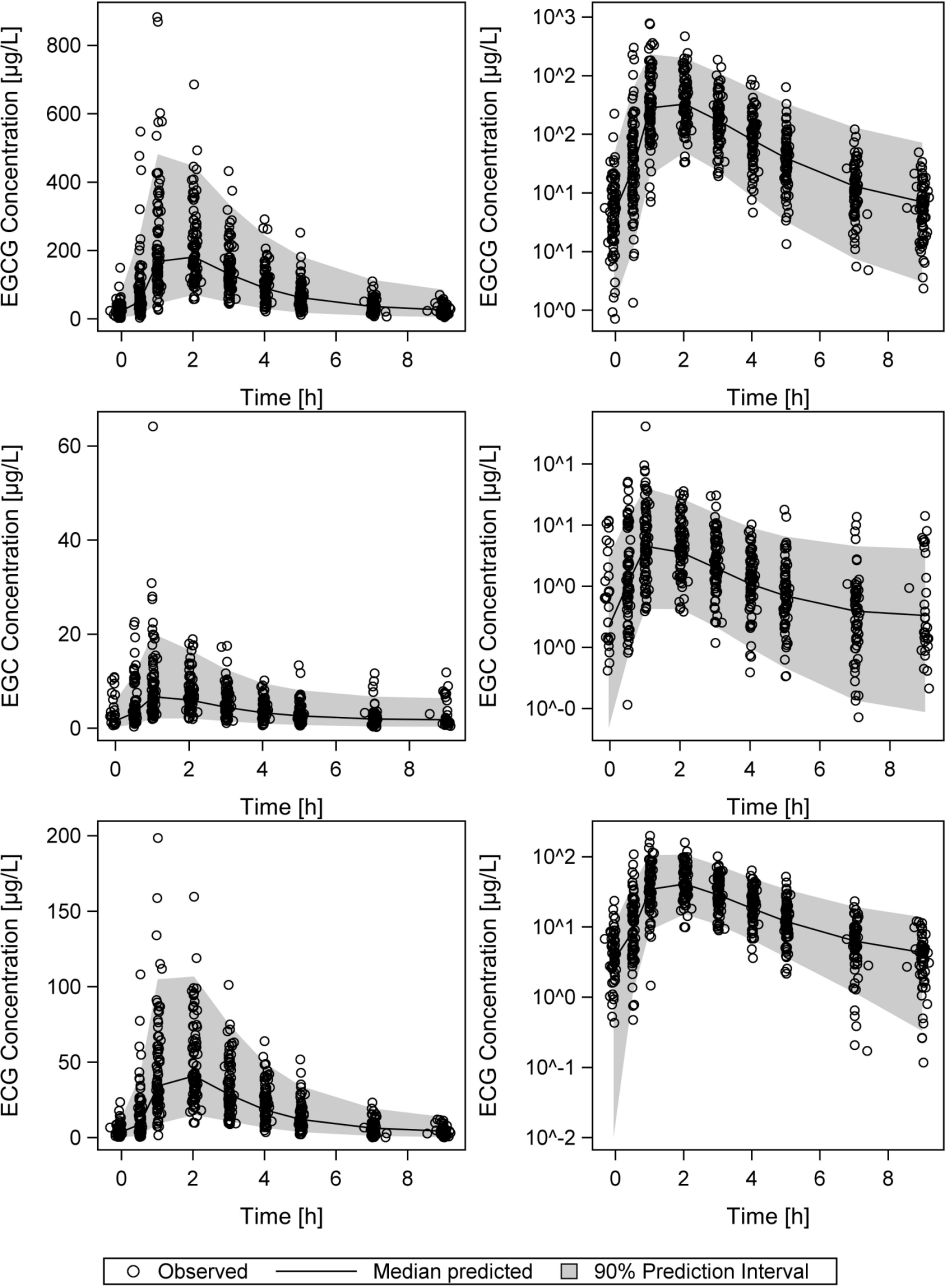
Visual predictive checks of the simulation of 1000 individual plasma concentration-time profiles. Visual predictive checks showing the medians (black lines) and corresponding 90% prediction intervals (shaded grey areas) of the simulation of 1000 individual plasma concentration-time profiles using the final PopPK model parameters of EGCG, EGC and ECG, overlaid with the respective observed data (circles). Linear (left panel) and logarithmic (right panel) presentation of plasma concentrations.

**Fig 7:**
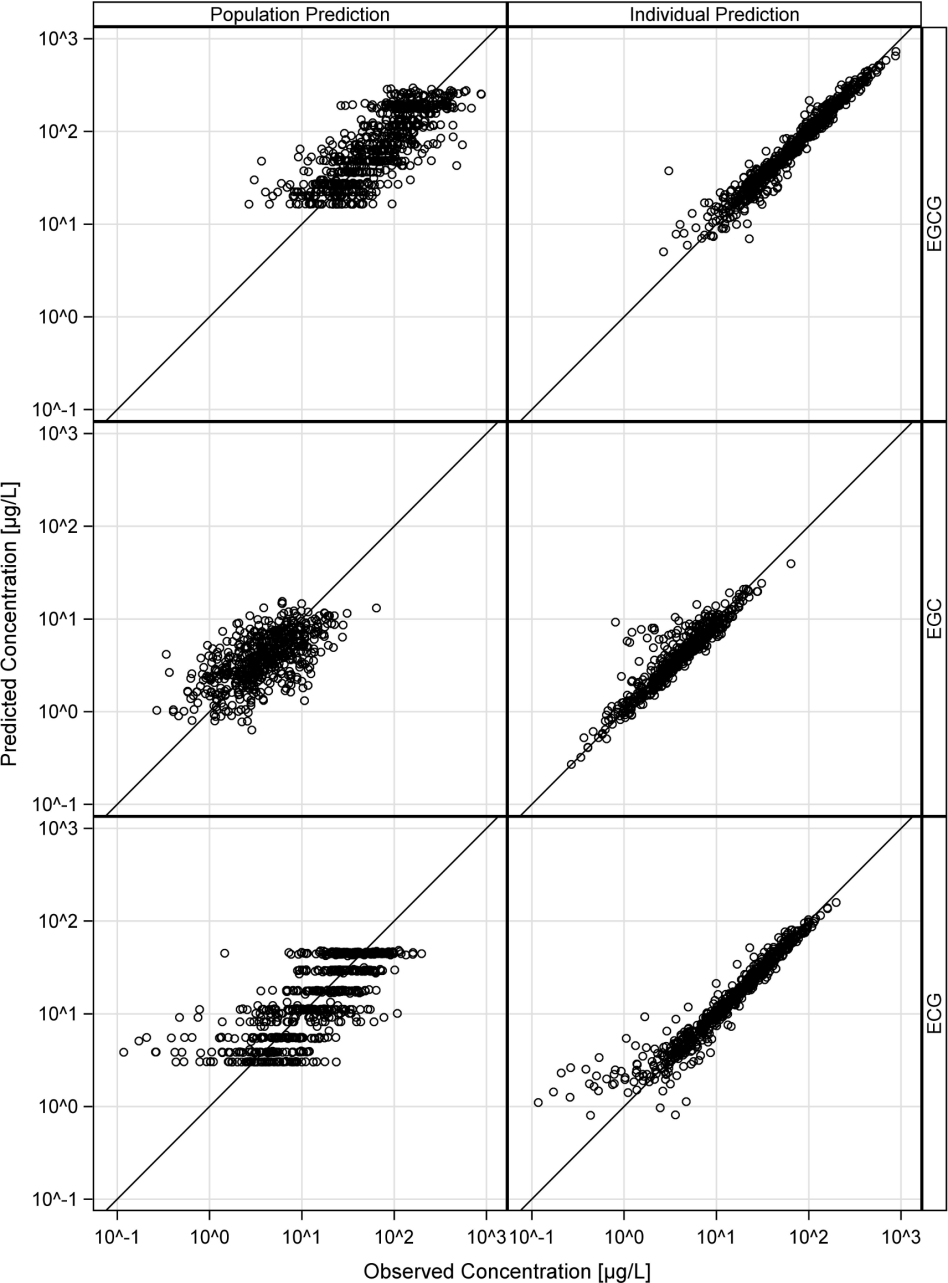
Goodness-of-fit plots of the final PopPK models of EGCG, EGC and ECG. Observed (x-axis) vs. predicted (y-axis) plasma concentrations (black circles) scattered around the line of identity. Population predictions (left panel) and individual plasma concentration predictions (right panel) of all three catechins.

The VPCs in Fig 6 demonstrate good descriptive performance of all three models with neither bias nor under- or overestimation of the model variabilities. Population and individual observed versus predicted plasma concentrations (goodness-of-fit plots, Fig 7) are randomly distributed around the line of identity, indicating good descriptive properties. Plots of conditional weighted residuals are shown in the modeling supplement (S5 File).

Covariate screening revealed a significant influence of contraceptives on V_central_/F of the EGC dose (55% reduction of V_central_/F with contraception compared to V_central_/F of men and women not using oral contraceptives, p<0.0001). The most likely physiologic interpretation of this is that oral contraceptives inhibit membrane transporter (Pgp and MRP2) involved in the excretion of EGC. Including this covariate into the model explained 10% of the interindividual variability in V_central_/F.

We genotyped several functional polymorphism in genes coding for the drug transporters MRP2, OATP1B1, Pgp and the phase II metabolizing enzymes COMT, SULT1A1 and UGT1A1, that are suggested to be involved in the transport and biotransformation of green tea catechins. All genotypes were in Hardy–Weinberg equilibrium and allele frequencies were comparable to those reported in the literature for Europeans (Table 4).

**Table 4:** Distribution of allele frequencies in the study population.

	SNP	Genotype			Allele frequency		HWE (p- value)
**ABCC2 (MRP2)**	**rs2273697**	**GG**	**GA**	**AA**	**G**	**A**	** **
		50	30	4	0.77	0.23	0.92
	**rs3740066**	**CC**	**CT**	**TT**	**C**	**T**	
		40	32	12	0.67	0.33	0.26
	rs717620*	**CC**	**CT**	**TT**	**C**	**T**	
		58	22	3	0.82	0.17	0.85
	**rs8187710**	**GG**	**GA**	**AA**	**G**	**A**	
		77	7	0	0.96	0.04	0.35
**SLCO1B1 (OATP1B1)**	**rs4149056**	**TT**	**CT**	**CC**	**T**	**C**	** **
		54	27	3	0.80	0.20	0.88
	**rs2306283**	**AA**	**GA**	**GG**	**A**	**G**	** **
		29	40	15	0.58	0.42	0.95
	**rs11045819**	**CC**	**AC**	**AA**	**C**	**A**	** **
		63	20	1	0.87	0.13	0.92
**ABCB1 (PGP)**	**rs1045642**	**TT**	**CT**	**CC**	**T**	**C**	** **
		21	43	20	0.51	0.49	0.94
	**rs1128503**	**TT**	**CT**	**CC**	**T**	**C**	** **
		19	38	27	0.45	0.55	0.53
	**rs2032582**	**TT**	**GT**	**GG**	**T**	**G**	** **
		22	38	24	0.49	0.51	0.48
**COMT**	**rs4680**	**GG**	**GA**	**AA**	**G**	**A**	** **
		14	47	23	0.45	0.55	0.3
**SULT1A1**	**rs750155**	**GG**	**GA**	**AA**	**C**	**T**	** **
		18	45	21	0.48	0.52	0.61
UGT1A1*28: (TA)7 repeat	**rs8175347**	**(-)**	**(-/TA)**	**TA**	**(-)**	**TA**	** **
		37	37	10	0.66	0.34	0.95

*for one individual genotype could not be determined, SNP: single nucleotide polymorphism, HWE: Hardy-Weinberg Equilibrium

Lowering our level of significance requirement in the covariate screening to p≤0.025 for forward inclusion and backward elimination, several polymorphisms with influence on the kinetic of EGCG and EGC were identified. Relative bioavailability and CL/F of EGCG were influenced by *ABCC2* -24C>T (MRP2_rs717620) and *SLCO1B1* 521T>C (OATP1B1_rs4149056) genotypes, respectively. Wildtype carriers of *ABCC2* -24C>T showed 26% less relative bioavailability than carriers of the variant allele (p = 0.00721). In addition, CL/F of EGCG was lower in carriers of the C allele of *SLCO1B1* 521T>C (p = 0.01212, see Table 5).

**Table 5:** Covariates included into the final models.

P-value	Covariate	Affected parameter	Change of parameter
**EGCG final model**			
0.00721	MRP2_rs717620	F	CC: -26%
0.01212	OATP1B1_rs4149056	CL/F	TT: -0%, CT: -15%, CC: -30%
**EGC final model**			
0.00001	Contraceptives	V_central_/F	-55%
0.00153	MRP2_rs3740066	Q/F	CC: +133%
0.00163	OATP1B1_rs2306283	CL/F	AA: -35%
0.01654	MRP2_rs717620	V_central_/F	CC: -0%, CT: -37%, TT: -74%
0.01795	UGT1A1*28	CL/F	(-): -26%
0.02350	COMT_rs4680	F	AA: -24%

CL: clearance from central compartment, F: relative bioavailability, ka: absorption time constant, Lag time: delay in absorption, Q: intercompartmental clearance, V: volume of distribution. Transporter and enzyme covariates were included into the final model if p≤0.025 during backward elimination

Two genotypes of *ABCC2* (-24C>T and 3972C>T (MRP2_rs3740066)), one genotype of *SLCO1B1* (388A>C (OATP1B1_rs2306283)), *UGT1A1* (rs8175347) and *COMT* (rs4680) affected the kinetic of EGC. Wildtype carriers of *SLCO1B1* 388A>C and *UGT1A1* rs8175347 showed a 35% (p = 0.00163) and 26% (p = 0.01795) reduction in CL/F of EGC, respectively. In addition, wildtype carriers of *ABCC2* 3972C>T exhibited a higher Q/F compared to the remaining population. An influence of *ABCC2* -24C>T on V_central_/F of EGC was also determined, showing a reduced V_central_/F in carriers of the T allele (Table 5). Furthermore, EGC relative bioavailability was reduced 24% in carriers of the low activity AA genotype of *COMT* compared to the remaining population. No covariate was identified for the ECG model. Habitual tea drinking did not affect the kinetic of any of the catechins.

**Fig 8:**
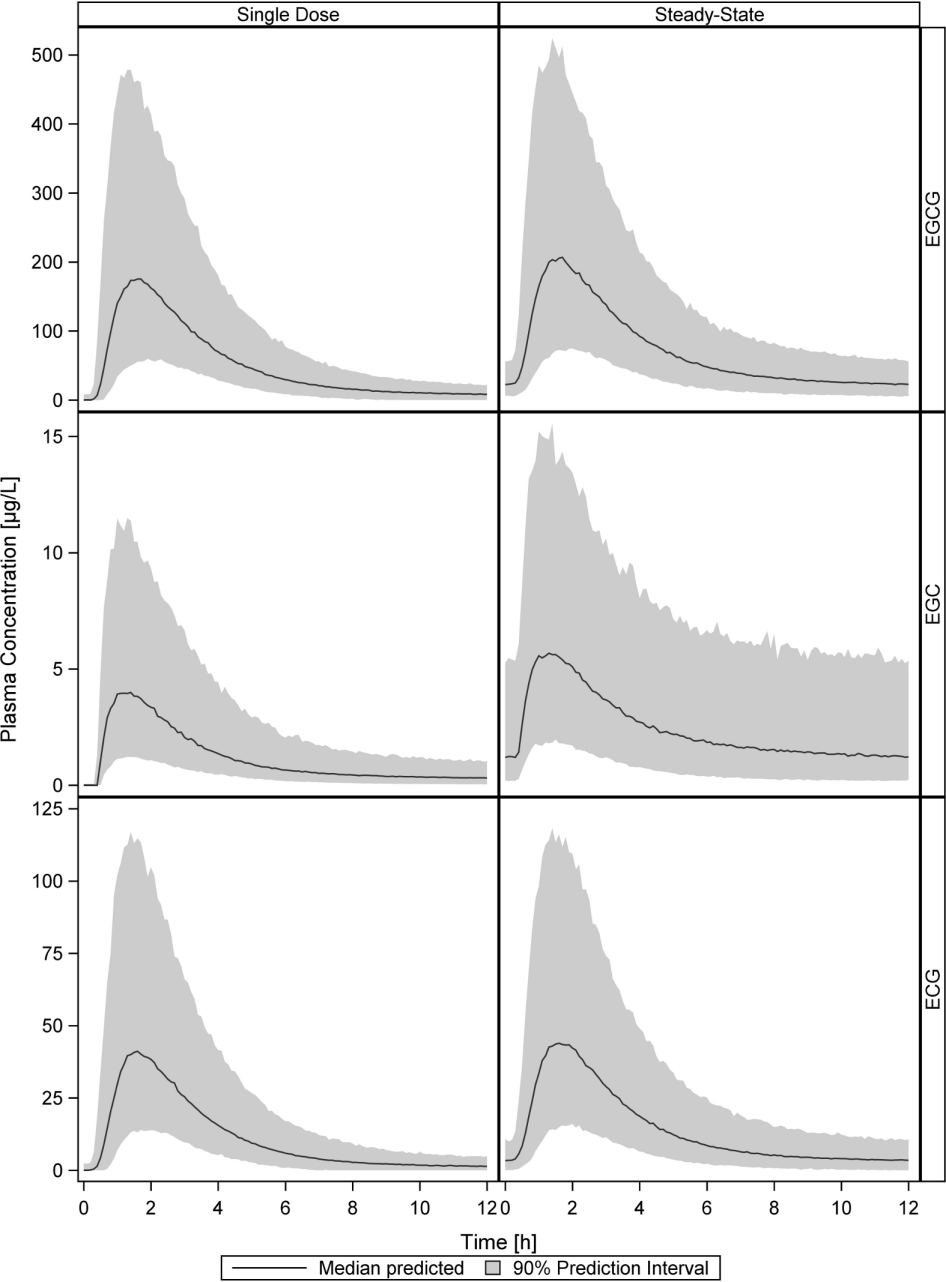
Simulation of single dose and steady-state plasma concentrations using the final PopPK model parameters of EGCG, EGC and ECG. Shown are the predicted medians (black lines) and corresponding 90% prediction intervals (shaded grey areas) of the simulation of 1000 individual plasma concentration-time profiles for each green tea catechin.

Exposure of EGCG, EGC and ECG after a single dose of green tea extract and under steady-state conditions was predicted based on the final PopPK models. For all three catechins only little accumulation could be observed at steady-state after dosing every 12 hours (Fig 8). The maximum increase of AUC_0-12_ found was twofold, median AUC values for single dose and steady-state conditions respectively are 657.0 and 902.0 h*μg/L for EGCG, 15.8 and 30.4 h*μg/L for EGC and 144.2 and 176.7 h*μg/L for ECG.

## Discussion

In epidemiological and observational studies health beneficial effects of green tea have been shown. Interindividual variation in plasma concentrations could be an explanation for in parts contradictory effects seen in different populations or diseases. Our population-based analysis of plasma concentrations of green tea catechins might help to understand this interindividual variability. Population-based nutrikinetics modeling of polyphenols has previously been described after consumption of polyphenol rich extracts, like grape wine or black tea extracts. In these nutrikinetics models catechins and metabolites generated by gut microbial bioconversion were measured in plasma and urine to integrate metabolomics and nutrikinetics [41,42]. Here we present the largest population-based nutrikinetic study of green tea catechins in plasma samples from 84 healthy volunteers consuming standardized green tea extract capsules after multiple dosing. Our study design allows a precise estimation of interindividual variation in pharmacokinetic parameters of green tea catechins on a population-based level and, due to the high number of participants, gives us the opportunity to study if individual pharmacogenetic variations in drug transport and metabolism may explain interindividual variations in plasma concentrations of green tea catechins.

With half lifes of about 3 hours, pharmacokinetic steady state is already achieved after one day, but 5 days were choosen to consider possible alterations in absorption due to transcriptional changes in membrane transporters or metabolizing enzymes. Considering the plasma concentrations, green tea catechins seem to have a low bioavailability, as it was also previously suggested [13,17,22]. No substance appropriately pure and safe for intravenous administration were available to us and without intravenous administration, no precise estimation of the absolute bioavailability can be made from the present study. However, considering that these hydrophilic substances will probably distribute initially only in the extracellular space (about 20 l), with the central volume of distribution estimated as 425 l for EGCG, one may estimate that less than 5% of EGCG is absorbed from the gut into the systemic circulation. Catechins were absorbed with moderate velocity and quickly eliminated, with maximum plasma concentrations measured after approximately 1.6 h and terminal half-lifes of 2.6 h, 3.9 h and 1.8 h for EGCG, EGC and ECG, respectively (Table 2). Accordingly, consistent with the findings of other groups, in our study only a small degree of accumulation after twice daily dosing was found, with a 1.4-fold increase of EGCG AUC_0-12_, a 1.9-fold increase of EGC AUC_0-12_ and a 1.2-fold increase of ECG AUC_0-12_ [8,11].

AUC values, volumes of distribution and total body clearances were significantly correlated between the different catechins, with the strongest associations between kinetic parameters of EGCG and ECG. EGCG, EGC and ECG share a similar chemical structure, varying only in the number of hydroxyl group substitutions in the B ring of their flavan-3-ol structure (epigallocatechin: tri-hydroxyl, epicatechin: di-hydroxyl) and the gallate substitution [1]. A conversion of gallate to non-gallate forms may also be possible [14]. This similarity in structure is most probably responsible for comparable kinetics of the green tea catechins, however the two gallate catechins (EGCG and ECG) clearly distinguished from the non-gallate form (EGC).

The best model for the description of EGCG and ECG absorption turned out to be a sequential zero-order, first-order absorption process with lag time, whereas EGC absorption was better described by a model lacking the zero-order process suggesting different dissolution behavior or different sites of absorption of gallate and non-gallate forms in the small intestine. Normalizing plasma levels to the doses of EGCG, EGC and ECG in the green tea capsules reveals higher relative plasma levels for EGCG and ECG than for EGC. This finding is in contrast to previous publications showing lower relative plasma levels for EGCG; however, in these former studies free and conjugated forms of each catechin were quantified together [12,14,15]. *In vitro* studies, investigating the transport of tea catechins across Caco-2 cells, a model of the intestinal epithelium, found higher efflux rates for a non-gallate catechin (EC) than for the corresponding gallate form (ECG) [22,43,44], suggesting the gallate moiety to be responsible for this difference. Lower efflux rates of gallate catechin (ECG) are associated with an higher cellular accumulation compared to EC [44]. It has also been described that catechins containing a gallate moiety are present in the plasma mainly as unconjugated forms whereas non-gallate catechins are usually conjugated [12,17,22]. In general conjugation may facilitate elimination; this could additionally contribute to the finding of higher relative plasma levels found for EGCG and ECG. As we measured only unconjugated catechins in the plasma it can not be excluded that total level of EGC may be much higher.

We saw a high intersubject variability in the nutrikinetics of EGCG, EGC and ECG. Maximum plasma concentrations (C_max_) varied 6.1, 7.7 and 6.6-fold for EGCG, EGC and ECG within the study population as measured by their 5^th^ to 95^th^ percentiles (Table 2). This variability is in agreement with previous studies investigating the pharmacokinetics of green tea catechins in considerably smaller populations [11,22,45]. For EGCG and ECG the highest interindividual variabilities in the compartmental analysis were detected on the zero-and first-order absorption processes, whereas for EGC the interindividual variability was high for the central volume of distribution and the intercompartmental distribution. However, with oral administration both parameters depend on the bioavailablity. Differences in the intestinal absorption of gallate and non-gallate catechins caused by an inhibition of efflux transporters (e.g. MRP2, Pgp) by the gallate moiety, could be one plausible explanation for the observed difference in the interindividual variability. Highly variable blood concentrations of green tea polyphenols may be relevant to understand interindividual effects in cardiovascular or metabolic prevention but may also be considered in respect to adverse systemic effects, like liver damage. Reviews assessing the liver-related safety of green tea show only mild liver-related events after intake of green tea extract [46], but also a few severe cases of liver injury have recently been reported occurring mostly after intake of high amounts of green tea over a long time period [47]. Considering our results a multiple dosing regime with several low doses of green tea extract per day could be advisable to reduce the risk of adverse reactions.

In the presented covariate analysis, gender, age, weight, height and BMI did not influence the kinetics of EGCG, EGC or ECG, neither did the tested lifestyle parameters smoking, alcohol consumption or regular consumption of green tea before the study. Interestingly, use of contraceptives significantly reduced V_central_/F of EGC (55% reduction, p<0.0001). In contrast, no influence of contraceptives on V_central_/F of EGCG or ECG was found. 69% of the women participating in the study stated the use of contraceptives. In several publications the effect of sexual hormones on drug transporters was investigated. *In vitro*, progestins were shown to inhibit Pgp and MRP2 [38,39] whereas pharmacologic concentrations of ethinylestradiol increase the expression and activity MRP2 [40]. Pgp, MRP1 and MRP2 also seem to be involved in the absorption and excretion of green tea catechins. In Caco-2 cells it was demonstrated that EC, and to a lower extend also ECG, are transported by Pgp and MRP2 [44]. Investigations in MDCKII cells overexpressing human Pgp, MRP1 and MRP2 genes indicate that MRP1 and MRP2 are transporters for EGCG and its methylated metabolites [24,35]. The use of oral contraceptives might therefore be responsible for an altered transport of green tea catechins which could lead to differences in the distribution and elimination of EGC. Investigating genetic polymorphisms in genes coding for drug transporters that might be involved in the active transport of green tea catechins in the intestine and liver revealed that V_central_/F of EGC was also reduced in carriers of the low functional T allele of MRP2 -24C>T. The polymorphism -24C>T is located in the 5’ untranslated region (UTR) of the *ABCC2* gene and several *in vivo* studies demonstrated an impact of this polymorphism on the pharmacokinetic of different drugs (e.g. methotrexate, mycophenolic acid, irinotecan) [48]. In addition we found that genetic polymorphisms in the *SLCO1B1* gene coding for OATP1B1 were associated with the clearance of EGCG and EGC (CL/F of EGCG: 30% reduction in homozygous carriers of the C allele of *SLCO1B1* 521T>C (SLCO1B1*5), p = 0.01212; CL/F of EGC: 35% reduction in the wildtype compared to the variant allele *SLCO1B1* 388A>G (SLCO1B1*1B) allele, p = 0.00163). OATP1B1 is expressed predominantly on the basolateral membrane of hepatocytes and mediates active intracellular hepatic uptake of various anionic compounds [49]. *In vitro* studies suggested a lower function of the SLCO1B1*5 variant compared to wildtype [50], in agreement with our finding of a lower CL/F of EGCG with this genotype. The fact that CL/F of EGC was reduced in wildtype individuals in comparison to carriers of the variant allele SLCO1B1*1B is also in accordance with several publications indicating that SLCO1B1*1B is associated with an increased transport function of OATP1B1, even if reports on the effect of SLCO1B1*1B are inconsistent and substrate dependent [51]. *In vitro* studies suggested green tea catechins to be potent inhibitors of OATP1B1, but our results indicate that OATP1B1 could not only be inhibited by catechins but could also be involved in their hepatic clearance.

Regarding the effect of genetic polymorphisms of the main phase II enzymes involved in the biotransformation of green tea catechins (COMT, SULT1A1, UGT1A1) we observed only a weak impact on the kinetics of EGC. We could identify a 24% reduction in bioavailability of EGC in carriers of the low activity genotype Met/Met of COMT as well as a 26% reduction of CL/F in the wildtype carriers of *UGT1A1* rs8175347. The influence of *COMT* rs4680 genotype on the kinetic of EGCG or green tea catechins has been addressed in a few previous studies [29,52,53], but these studies identified also no or only small effects of *COMT* rs4680 genotype on kinetic parameters of tea catechins. Considering that methylation, sulfation and glucuronidation play an interesting role in catechin biotransformation, it was most obvious to analyze known functional polymorphisms within this genes. However, these polymorphisms had no effects on EGCG and ECG kinetics and statistically weak and counterintuitive effects on EGC. Since we do not have a convincing explanation for the latter findings, the best explanation may be that there is no relevant effect on all three catechins.

In summary, the plasma concentration-time profiles and their interindividual variation of EGCG, EGC and ECG after twice daily oral administration could be described in a large sample of healthy volunteers. Within the very homogenous study population a high intersubject variability of plasma concentrations was revealed. We found that the use of contraceptives and inherent genetic variations in genes coding for MRP2 and OATP1B1 impact on the pharmacokinetic of EGCG and EGC even if this explains only a part of the large intersubject variability. The investigated polymorphisms in the genes of Pgp and SULT1A1 showed no significant influence on the pharmacokinetic parameters of EGCG, EGC and ECG. Further explanations of the large variability in green tea catechin plasma concentrations might be interindividual differences in gastrointestinal solubility or in the influence of the microbiome. The pharmacokinetic data obtained in this study may be important for extrapolation of the *in vitro* effects seen with green tea catechins to possible effects in humans. The highly variable blood concentrations of green tea catechins will be key to understand the individual pharmacodynamic effects in cardiovascular, metabolic or cancer prevention of green tea extract as well as in adverse reactions. The data may be useful to optimize and individualize future studies investigating health benefit of green tea catechins.

## Supporting information

S1 Table Blood concentrations of green tea catechins. (TXT)

S1 File Study protocol MIRACLE Study (german version). (PDF)

S2 File Study protocol nutrikinetics (german version). (PDF)

S3 File Study protocol nutrikinetics (english version). (PDF)

S4 File Trend checklist. (PDF)

S5 File Modeling supplement. (PDF)

## References

[1] Balentine DA, Wiseman SA, Bouwens LC (1997) The chemistry of tea flavonoids. Crit Rev Food Sci Nutr 37: 693–704. doi: 10.1080/10408399709527797 9447270 10.1080/10408399709527797

[2] Johnson R, Bryant S, Huntley AL (2012) Green tea and green tea catechin extracts: an overview of the clinical evidence. Maturitas 73: 280–287. doi: 10.1016/j.maturitas.2012.08.008 22986087 10.1016/j.maturitas.2012.08.008

[3] Kanwar J, Taskeen M, Mohammad I, Huo C, Chan TH, et al (2012) Recent advances on tea polyphenols. Front Biosci (Elite Ed) 4: 111–131. 22201858 10.2741/363 PMC3303150

[4] Yuan JM (2013) Cancer prevention by green tea: evidence from epidemiologic studies. Am J Clin Nutr 98: 1676S–1681S. doi: 10.3945/ajcn.113.058271 24172305 10.3945/ajcn.113.058271 PMC3831544

[5] Rahmani AH, Al Shabrmi FM, Allemailem KS, Aly SM, Khan MA (2015) Implications of Green Tea and Its Constituents in the Prevention of Cancer via the Modulation of Cell Signalling Pathway. Biomed Res Int 2015: 925640 doi: 10.1155/2015/925640 25977926 10.1155/2015/925640 PMC4419223

[6] Chow HH, Hakim IA (2011) Pharmacokinetic and chemoprevention studies on tea in humans. Pharmacol Res 64: 105–112. doi: 10.1016/j.phrs.2011.05.007 21624470 10.1016/j.phrs.2011.05.007 PMC3152306

[7] Stingl JC, Ettrich T, Muche R, Wiedom M, Brockmoller J, et al (2011) Protocol for minimizing the risk of metachronous adenomas of the colorectum with green tea extract (MIRACLE): a randomised controlled trial of green tea extract versus placebo for nutriprevention of metachronous colon adenomas in the elderly population. BMC Cancer 11: 360 doi: 10.1186/1471-2407-11-360 21851602 10.1186/1471-2407-11-360 PMC3176243

[8] Ullmann U, Haller J, Decourt JD, Girault J, Spitzer V, et al (2004) Plasma-kinetic characteristics of purified and isolated green tea catechin epigallocatechin gallate (EGCG) after 10 days repeated dosing in healthy volunteers. Int J Vitam Nutr Res 74: 269–278. doi: 10.1024/0300-9831.74.4.269 15580809 10.1024/0300-9831.74.4.269

[9] Ullmann U, Haller J, Decourt JP, Girault N, Girault J, et al (2003) A single ascending dose study of epigallocatechin gallate in healthy volunteers. J Int Med Res 31: 88–101. doi: 10.1177/147323000303100205 12760312 10.1177/147323000303100205

[10] Henning SM, Niu Y, Liu Y, Lee NH, Hara Y, et al (2005) Bioavailability and antioxidant effect of epigallocatechin gallate administered in purified form versus as green tea extract in healthy individuals. J Nutr Biochem 16: 610–616. doi: 10.1016/j.jnutbio.2005.03.003 16081270 10.1016/j.jnutbio.2005.03.003

[11] Chow HH, Cai Y, Hakim IA, Crowell JA, Shahi F, et al (2003) Pharmacokinetics and safety of green tea polyphenols after multiple-dose administration of epigallocatechin gallate and polyphenon E in healthy individuals. Clin Cancer Res 9: 3312–3319. 12960117

[12] Lee MJ, Maliakal P, Chen L, Meng X, Bondoc FY, et al (2002) Pharmacokinetics of tea catechins after ingestion of green tea and (-)-epigallocatechin-3-gallate by humans: formation of different metabolites and individual variability. Cancer Epidemiol Biomarkers Prev 11: 1025–1032. 12376503

[13] Fung ST, Ho CK, Choi SW, Chung WY, Benzie IF (2013) Comparison of catechin profiles in human plasma and urine after single dosing and regular intake of green tea (Camellia sinensis). Br J Nutr 109: 2199–2207. doi: 10.1017/S0007114512004370 23110850 10.1017/S0007114512004370

[14] Warden BA, Smith LS, Beecher GR, Balentine DA, Clevidence BA (2001) Catechins are bioavailable in men and women drinking black tea throughout the day. J Nutr 131: 1731–1737. 11385060 10.1093/jn/131.6.1731

[15] Renouf M, Guy P, Marmet C, Longet K, Fraering AL, et al (2010) Plasma appearance and correlation between coffee and green tea metabolites in human subjects. Br J Nutr 104: 1635–1640. doi: 10.1017/S0007114510002709 20691128 10.1017/S0007114510002709

[16] Henning SM, Wang P, Abgaryan N, Vicinanza R, de Oliveira DM, et al (2013) Phenolic acid concentrations in plasma and urine from men consuming green or black tea and potential chemopreventive properties for colon cancer. Mol Nutr Food Res 57: 483–493. doi: 10.1002/mnfr.201200646 23319439 10.1002/mnfr.201200646 PMC3600069

[17] Chow HH, Hakim IA, Vining DR, Crowell JA, Ranger-Moore J, et al (2005) Effects of dosing condition on the oral bioavailability of green tea catechins after single-dose administration of Polyphenon E in healthy individuals. Clin Cancer Res 11: 4627–4633. doi: 10.1158/1078-0432.CCR-04-2549 15958649 10.1158/1078-0432.CCR-04-2549

[18] Nakagawa K, Okuda S, Miyazawa T (1997) Dose-dependent incorporation of tea catechins, (-)-epigallocatechin-3-gallate and (-)-epigallocatechin, into human plasma. Biosci Biotechnol Biochem 61: 1981–1985. 9438978 10.1271/bbb.61.1981

[19] Hodgson AB, Randell RK, Mahabir-Jagessar TK, Lotito S, Mulder T, et al (2014) Acute effects of green tea extract intake on exogenous and endogenous metabolites in human plasma. J Agric Food Chem 62: 1198–1208. doi: 10.1021/jf404872y 24400998 10.1021/jf404872y

[20] Chen L, Lee MJ, Li H, Yang CS (1997) Absorption, distribution, elimination of tea polyphenols in rats. Drug Metab Dispos 25: 1045–1050. 9311619

[21] Lambert JD, Lee MJ, Lu H, Meng X, Hong JJ, et al (2003) Epigallocatechin-3-gallate is absorbed but extensively glucuronidated following oral administration to mice. J Nutr 133: 4172–4177. 14652367 10.1093/jn/133.12.4172

[22] Henning SM, Choo JJ, Heber D (2008) Nongallated compared with gallated flavan-3-ols in green and black tea are more bioavailable. J Nutr 138: 1529S–1534S. 18641202 10.1093/jn/138.8.1529S PMC2942025

[23] Sang S, Lambert JD, Ho CT, Yang CS (2011) The chemistry and biotransformation of tea constituents. Pharmacol Res 64: 87–99. doi: 10.1016/j.phrs.2011.02.007 21371557 10.1016/j.phrs.2011.02.007

[24] Lambert JD, Sang S, Yang CS (2007) Biotransformation of green tea polyphenols and the biological activities of those metabolites. Mol Pharm 4: 819–825. doi: 10.1021/mp700075m 17963356 10.1021/mp700075m

[25] van Duynhoven J, Vaughan EE, Jacobs DM, Kemperman RA, van Velzen EJ, et al (2011) Metabolic fate of polyphenols in the human superorganism. Proc Natl Acad Sci U S A 108 Suppl 1: 4531–4538. 20615997 10.1073/pnas.1000098107 PMC3063601

[26] Chen D, Wang CY, Lambert JD, Ai N, Welsh WJ, et al (2005) Inhibition of human liver catechol-O-methyltransferase by tea catechins and their metabolites: structure-activity relationship and molecular-modeling studies. Biochem Pharmacol 69: 1523–1531. doi: 10.1016/j.bcp.2005.01.024 15857617 10.1016/j.bcp.2005.01.024

[27] Lu H, Meng X, Yang CS (2003) Enzymology of methylation of tea catechins and inhibition of catechol-O-methyltransferase by (-)-epigallocatechin gallate. Drug Metab Dispos 31: 572–579. 12695345 10.1124/dmd.31.5.572

[28] Nagai M, Conney AH, Zhu BT (2004) Strong inhibitory effects of common tea catechins and bioflavonoids on the O-methylation of catechol estrogens catalyzed by human liver cytosolic catechol-O-methyltransferase. Drug Metab Dispos 32: 497–504. doi: 10.1124/dmd.32.5.497 15100171 10.1124/dmd.32.5.497

[29] Lorenz M, Paul F, Moobed M, Baumann G, Zimmermann BF, et al (2014) The activity of catechol-O-methyltransferase (COMT) is not impaired by high doses of epigallocatechin-3-gallate (EGCG) in vivo. Eur J Pharmacol 740: 645–651. doi: 10.1016/j.ejphar.2014.06.014 24972245 10.1016/j.ejphar.2014.06.014

[30] Lee MJ, Wang ZY, Li H, Chen L, Sun Y, et al (1995) Analysis of plasma and urinary tea polyphenols in human subjects. Cancer Epidemiol Biomarkers Prev 4: 393–399. 7655336

[31] Vaidyanathan JB, Walle T (2002) Glucuronidation and sulfation of the tea flavonoid (-)-epicatechin by the human and rat enzymes. Drug Metab Dispos 30: 897–903. 12124307 10.1124/dmd.30.8.897

[32] Wang T, Cook I, Leyh TS (2016) Isozyme Specific Allosteric Regulation of Human Sulfotransferase 1A1. Biochemistry 55: 4036–4046. doi: 10.1021/acs.biochem.6b00401 27356022 10.1021/acs.biochem.6b00401 PMC6379071

[33] Coughtrie MW, Johnston LE (2001) Interactions between dietary chemicals and human sulfotransferases-molecular mechanisms and clinical significance. Drug Metab Dispos 29: 522–528. 11259344

[34] Lu H, Meng X, Li C, Sang S, Patten C, et al (2003) Glucuronides of tea catechins: enzymology of biosynthesis and biological activities. Drug Metab Dispos 31: 452–461. 12642472 10.1124/dmd.31.4.452

[35] Hong J, Lambert JD, Lee SH, Sinko PJ, Yang CS (2003) Involvement of multidrug resistance-associated proteins in regulating cellular levels of (-)-epigallocatechin-3-gallate and its methyl metabolites. Biochem Biophys Res Commun 310: 222–227. 14511674 10.1016/j.bbrc.2003.09.007

[36] Vaidyanathan JB, Walle T (2003) Cellular uptake and efflux of the tea flavonoid (-)epicatechin-3-gallate in the human intestinal cell line Caco-2. J Pharmacol Exp Ther 307: 745–752. doi: 10.1124/jpet.103.054296 12970388 10.1124/jpet.103.054296

[37] Knop J, Misaka S, Singer K, Hoier E, Muller F, et al (2015) Inhibitory Effects of Green Tea and (-)-Epigallocatechin Gallate on Transport by OATP1B1, OATP1B3, OCT1, OCT2, MATE1, MATE2-K and P-Glycoprotein. PLoS One 10: e0139370 doi: 10.1371/journal.pone.0139370 26426900 10.1371/journal.pone.0139370 PMC4591125

[38] Albassam AA, Markowitz JS (2017) An Appraisal of Drug-Drug Interactions with Green Tea (Camellia sinensis). Planta Med 83: 496–508. doi: 10.1055/s-0043-100934 28118673 10.1055/s-0043-100934

[39] Streit F, Binder L, Hafke A, Brandhorst G, Braulke F, et al (2011) Use of total and unbound imatinib and metabolite LC-MS/MS assay to understand individual responses in CML and GIST patients. Ther Drug Monit 33: 632–643. doi: 10.1097/FTD.0b013e3182263ac4 21912334 10.1097/FTD.0b013e3182263ac4

[40] Sheiner LB (1984) Analysis of pharmacokinetic data using parametric models—1: Regression models. J Pharmacokinet Biopharm 12: 93–117. 6747821 10.1007/BF01063613

[41] van Velzen EJ, Westerhuis JA, van Duynhoven JP, van Dorsten FA, Grun CH, et al (2009) Phenotyping tea consumers by nutrikinetic analysis of polyphenolic end-metabolites. J Proteome Res 8: 3317–3330. doi: 10.1021/pr801071p 19374449 10.1021/pr801071p

[42] van Velzen EJJ, Westerhuis JA, Grün CH, Jacobs DM, Eilers PHC, et al (2014) Population-based nutrikinetic modeling of polyphenol exposure. Metabolomics 10: 1059–1073.

[43] Tagashira T, Choshi T, Hibino S, Kamishikiryou J, Sugihara N (2012) Influence of gallate and pyrogallol moieties on the intestinal absorption of (-)-epicatechin and (-)-epicatechin gallate. J Food Sci 77: H208–215. doi: 10.1111/j.1750-3841.2012.02902.x 22938538 10.1111/j.1750-3841.2012.02902.x

[44] Kadowaki M, Sugihara N, Tagashira T, Terao K, Furuno K (2008) Presence or absence of a gallate moiety on catechins affects their cellular transport. J Pharm Pharmacol 60: 1189–1195. doi: 10.1211/jpp.60.9.0011 18718123 10.1211/jpp.60.9.0011

[45] Chow HH, Cai Y, Alberts DS, Hakim I, Dorr R, et al (2001) Phase I pharmacokinetic study of tea polyphenols following single-dose administration of epigallocatechin gallate and polyphenon E. Cancer Epidemiol Biomarkers Prev 10: 53–58. 11205489

[46] Isomura T, Suzuki S, Origasa H, Hosono A, Suzuki M, et al (2016) Liver-related safety assessment of green tea extracts in humans: a systematic review of randomized controlled trials. Eur J Clin Nutr. 10.1038/ejcn.2016.165 PMC7608275 27805619

[47] Mazzanti G, Di Sotto A, Vitalone A (2015) Hepatotoxicity of green tea: an update. Arch Toxicol 89: 1175–1191. doi: 10.1007/s00204-015-1521-x 25975988 10.1007/s00204-015-1521-x

[48] Bruhn O, Cascorbi I (2014) Polymorphisms of the drug transporters ABCB1, ABCG2, ABCC2 and ABCC3 and their impact on drug bioavailability and clinical relevance. Expert Opin Drug Metab Toxicol 10: 1337–1354. doi: 10.1517/17425255.2014.952630 25162314 10.1517/17425255.2014.952630

[49] Konig J, Cui Y, Nies AT, Keppler D (2000) A novel human organic anion transporting polypeptide localized to the basolateral hepatocyte membrane. Am J Physiol Gastrointest Liver Physiol 278: G156–164. doi: 10.1152/ajpgi.2000.278.1.G156 10644574 10.1152/ajpgi.2000.278.1.G156

[50] Lee HH, Ho RH Interindividual and interethnic variability in drug disposition: polymorphisms in organic anion transporting polypeptide 1B1 (OATP1B1; SLCO1B1). Br J Clin Pharmacol 83: 1176–1184. doi: 10.1111/bcp.13207 27936281 10.1111/bcp.13207 PMC5427225

[51] Gong IY, Kim RB Impact of genetic variation in OATP transporters to drug disposition and response. Drug Metab Pharmacokinet 28: 4–18. 23047721 10.2133/dmpk.dmpk-12-rv-099

[52] Inoue-Choi M, Yuan JM, Yang CS, Van Den Berg DJ, Lee MJ, et al (2010) Genetic Association Between the COMT Genotype and Urinary Levels of Tea Polyphenols and Their Metabolites among Daily Green Tea Drinkers. Int J Mol Epidemiol Genet 1: 114–123. 21191472 PMC3010377

[53] Miller RJ, Jackson KG, Dadd T, Nicol B, Dick JL, et al (2012) A preliminary investigation of the impact of catechol-O-methyltransferase genotype on the absorption and metabolism of green tea catechins. Eur J Nutr 51: 47–55. doi: 10.1007/s00394-011-0189-0 21445620 10.1007/s00394-011-0189-0

