# Supplementary material for: Population nutrikinetics of green tea extract
Source: PLoS One. 2018 Feb 21;13(2):e0193074. doi: 10.1371/journal.pone.0193074 (PMC5821365; doi:10.1371/journal.pone.0193074)
Supplement: S1 File — (PDF) [file pone.0193074.s002.pdf]

gefördert durch Deutsche Krebshilfe

Studienprotokoll

MIRACLE-Studie

Minimizing the Risk of Metachronous Adenomas of the Colorectum with Green Tea Extract

Präventionsstudie zu Grünteeextrakt als Nahrungsergänzungsmittel zur Vermeidung metachroner kolorektaler Adenome - Klinische Studie zu einem Nahrungsergänzungsmittel

Version:

23.6.2011 final

Registriernummer:

NCT01360320

Leiter der Studie:

Prof. Dr. med. Thomas Seufferlein
Direktor der Universitätsklinik und Poliklinik für Innere Medizin I
Martin-Luther-Universität Halle-Wittenberg
Ernst-Grube-Straße 40
06120 Halle (Saale)
Tel: +49 (0) 345 557 2661
Fax: +49 (0) 345 557 2253
E-Mail: thomas.seufferlein@uk-halle.de

Sponsor:

Martin-Luther-Universität Halle-Wittenberg
D-06099 Halle (Saale)
Tel: +49 (0) 345 557 5420
Fax: +49 (0) 345 557 5424

Datenmanagement und Monitoring:

Koordinierungszentrum für Klinische Studien der Medizinischen Fakultät der Martin-Luther-Universität Halle-Wittenberg
06097 Halle (Saale)
Tel: +49 (0) 345 557 4908
Fax: +49 (0) 345 557 5210
http://www.kks-halle.de

Der Inhalt des Studienplans ist vertraulich zu behandeln und darf ohne Zustimmung der Studienleitung weder mündlich noch schriftlich an Unbeteiligte weitergegeben werden.

Inhaltsverzeichnis

ABKÜRZUNGSVERZEICHNIS **5**

1. ÜBERSICHT **6**

1.1 ZUSAMMENFASSUNG 6
1.2 SYNOPSE 7
1.3 FLOW CHART 11
1.4 AUSGANGSSITUATION 11
1.4.1 In vitro Untersuchungen zu antiproliferativen Effekten von Grüntee-Katechinen *12*
1.4.2 In vivo Untersuchungen zu chemoprotektiven Eigenschaften von Grüntee-Katechinen *13*
1.4.3 Klinische Daten *13*
1.4.4 Grüntee zur Prävention kolorektaler Adenome *15*
1.4.5 Kinetische Untersuchungen zu Grüntee-Katechinen beim Menschen *15*
1.5 FRAGESTELLUNG UND BEGRÜNDUNG DES VORHABENS 17
1.6 ABSCHÄTZUNG DER ÜBERTRAGBARKEIT DER ERWARTETEN STUDIENERGEBNISSE AUF DIE PRAXIS 18
1.7 PRIMÄRES STUDIENZIEL 19
1.8 SEKUNDÄRE ZIELE 19
1.9 WISSENSCHAFTLICHES BEGLEITPROGRAMM 19
1.9.1 Zusatzprojekt „Biomarker und Nutrigenetik" *19*
1.9.2 Zusatzprojekt „Histologische Gewebebank" *20*
1.9.3 Zusatzprojekt „Nutrikinetik von Epigallokatechingallat (EGCG) in oraler Formulierung: Populationsvariabilität und Nutrigenomik" *20*

2 ALLGEMEINE ANGABEN ZUR STUDIE **20**

2.1 ART DER STUDIE / STUDIENDESIGN 20
2.2 ZAHL UND ART DER VERGLEICHSGRUPPEN 21
2.3 UMFANG DER STUDIE / MACHBARKEIT 21
2.4 PROBANDENREKRUTIERUNG 22
2.5 RANDOMISIERUNGSPRINZIP 22
2.6 ZEITPLAN 23
2.7 ZEITLICHES STUDIENABLAUFSCHEMA 24

3 STUDIENZENTREN **26**

3.1 TEILNEHMENDE ZENTREN 26
3.2 BEDINGUNGEN FÜR DIE TEILNAHME AN DER STUDIE 26

4 AUSWAHL DER PROBANDEN **26**

4.1 EINSCHLUSSKRITERIEN 27
4.2 AUSSCHLUSSKRITERIEN 27

5 AUFNAHME/REGISTRIERUNG UND RANDOMISATION **28**

5.1 ENROLMENT UND RUN-IN 28
5.2 RANDOMISATION 28
5.3 PROBENENTNAHME FÜR BIOMATERIALBANKEN 29
5.3.1 Im Rahmen des Zusatzprojektes „Biomarker und Nutrigenetik" *29*
5.3.2 Im Rahmen des Zusatzprojektes „Histologische Gewebebank" *30*
5.4 LOGISTIK DES VERSANDS DER STUDIENSUBSTANZ 32

6 BEHANDLUNGSPLAN **32**

6.1 BESCHREIBUNG DES GRÜNTEEEXTRAKTES: 32
6.2 KENNZEICHNUNG, BESCHRIFTUNG 33
6.3 DOKUMENTATION ÜBER DEN VERBLEIB DER PRÜFPRÄPARATE 34
6.4 NEBENWIRKUNGEN, KONTRAINDIKATIONEN 34
6.5 WECHSELWIRKUNG MIT ANDEREN SUBSTANZEN 35
6.6 PHARMAKOKINETIK 36
6.7 PHARMAKODYNAMIK 36
6.8 NOTFALLMAßNAHMEN 36

6.9 BEHANDLUNGSSCHEMA 37
6.10 BEGLEITTHERAPIE 37

7 KLINISCHE UNTERSUCHUNGEN 37
7.1 EINGANGS-/EINSCHLUSSUNTERSUCHUNG 37
7.2 VERLAUFSUNTERSUCHUNGEN 38
7.3 ABSCHLUSSUNTERSUCHUNG 39
7.4 STUDIENBEDINGTE BLUTABNAHMEN 39
7.5 REFERENZINSTITUTIONEN 40
7.6 SPÄTERER AUSSCHLUSS VON PROBANDEN 40

8 METHODEN ZUR ERFASSUNG DER WIRKSAMKEIT UND DER CHEMOPROTEKTIVEN EFFEKTIVITÄT 41
8.1 ZEITPLAN 41
8.2 WIRKSAMKEIT 41
8.3 INDIVIDUELLE UND ALLGEMEINE ABBRUCHKRITERIEN 42

9 METHODEN ZUR ERFASSUNG DER SICHERHEIT – UNERWÜNSCHTE EREIGNISSE 42
9.1 DEFINITIONEN UNERWÜNSCHTER EREIGNISSE 42
9.2 EINTEILUNG UNERWÜNSCHTER EREIGNISSE 43
9.2.1 Schweregrade unerwünschter Ereignisse 43
9.2.2 Kausalzusammenhang unerwünschter Ereignisse 43
9.2.3 Gegenmaßnahmen 44
9.2.4 Ausgang unerwünschter Ereignisse 44
9.3 ERFASSUNG UND DOKUMENTATION 44
9.4 BERICHTERSTATTUNG 45
9.4.1 Berichterstattung durch den Prüfarzt 45

10 DAUER DER STUDIE / STUDIENABBRUCH 46
10.1 REGULÄRES STUDIENENDE 46
10.2 VORZEITIGES STUDIENENDE / STUDIENABBRUCH 46

11 BIOMETRISCHE PLANUNG UND AUSWERTUNG 47
11.1 ZIELGRÖßEN 47
11.1.1 Primäres Studienziel 47
11.1.2 Sekundäre Ziele 47
11.2 DEFINITION VON AUSWERTUNGSKOLLEKTIVEN 47
11.2.1 Intention-to-treat Population 47
11.2.2 Per-protocol-Population 48
11.3 PLANUNG DES STUDIENUMFANGS 48
11.4 PLANUNG DER STATISTISCHEN AUSWERTUNG DER HAUPTZIELGRÖßE 49
11.5 EXPLORATIVE AUSWERTUNGEN DER SEKUNDÄREN ZIELGRÖßEN 49
11.6 SICHERHEITSANALYSE 50

12 DATENMANAGEMENT 50
12.1 DATENERFASSUNG, -AUSWERTUNG UND -WEITERGABE 50

13 QUALITÄTSSICHERUNG 51
13.1 ADVISORY BOARD 51
13.2 STANDARDISIERUNG, VALIDIERUNG UND MONITORING 51
13.3 MAßNAHMEN ZUR GEWÄHRLEISTUNG DER ADHÄRENZ UND KOHORTENPFLEGE 52

14 ETHISCHE BELANGE 53
14.1 BEGUTACHTUNG DES PRÜFPLANS DURCH DIE ZUSTÄNDIGE ETHIK-KOMMISSION 53
14.2 AUFKLÄRUNG UND EINWILLIGUNG DER PROBANDEN 53
14.3 VERWENDUNG, SPEICHERUNG UND WEITERGABE VON DATEN 54

15 GESETZLICHE UND ADMINISTRATIVE REGELUNGEN 54
15.1 VERSICHERUNG 54

15.2 FINANZIERUNG ... 54
15.3 PUBLIKATION ... 54
15.4 EINHALTUNG DES PROTOKOLLS UND PROTOKOLLÄNDERUNGEN ... 55

16 VERZEICHNIS DER MITWIRKENDEN / UNTERSCHRIFTEN ... 56

17 LITERATURVERZEICHNIS ... 58

18 APPENDICES ... 61

18.1 PROBANDENINFORMATION MIRACLE-STUDIE ... 61
18.2 PROBANDENINFORMATION ZUSATZPROJEKT „BIOMARKER UND NUTRIGENETIK“ ... 69
18.3 PROBANDENINFORMATION ZUSATZPROJEKT „HISTOLOGISCHE GEWEBEBANK“ ... 71
18.4 EINWILLIGUNGSERKLÄRUNG ... 73
18.5 INFORMATION UND EINWILLIGUNGSSERKLÄRUNG ZUM DATENSCHUTZ ... 74
18.6 ÜBEREIGNUNGSVERTRAG ZUSATZPROJEKT „BIOMARKER UND NUTRIGENETIK“ ... 75
18.7 ÜBEREIGNUNGSVERTRAG ZUSATZPROJEKT „HISTOLOGISCHE GEWEBEBANK“ ... 76
18.8 ETIKETT DER PRÜFSUBSTANZ ... 77
18.9 FRAGEBOGEN „ALTERNATIVE HEILMETHODEN” ... 78
18.10 ECOG-PERFORMANCE STATUS [50] ... 81
18.11 WORLD MEDICAL ASSOCIATION: DECLARATION OF HELSINKI [49] ... 82
18.12 VERSICHERUNGSBESCHEINIGUNGEN ... 86

Abkürzungsverzeichnis

AE	Adverse Event
ALT	Alanin-Aminotransferase
AMG	Arzneimittelgesetz
ASS	Acetysalicylsäure
AST	Aspartat-Aminotransferase
AUC	Area under the curve
COX 2	Cyclooxygenase 2
CRF	Case Report Form
CRO	Clinical Research Organization
Da	Dalton (atomare Maßeinheit)
DNA	Desoxyribonukleinsäure
DSMB	Data safety monitoring board
EC	Epicatechin
ECG	Epicatechin-3-gallat
ECOG	Eastern Cooperative Oncology Group
EDTA	Ethylendiamintetraessigsäure
EGC	Epigallocatechin
EGCG	Epigallocatechin-3-gallat
EGFR	Epidermal growth factor receptor
FAP	Familiäre adenomatöse Polyposis
FU	Follow-up
GCP	Good Clinical Practice
G-Protein	Guaninnucleotid-bindendes Protein
HER	Human epidermal growth factor receptor
HNPCC	Hereditäres non-polypöses Kolonkarzinom
ICH	International Conference on Harmonization
ITT	Intention-to-treat
KI	Konfidenz-Intervall
KKS	Koordinierungszentrum für Klinische Studien
KKSH	Koordinierungszentrum für Klinische Studien Halle
LR	Lamininrezeptor
LKP	Leiter der Klinischen Prüfung
MHD	Mindesthaltbarkeitsdatum
MLU	Martin Luther-Universität
MRP	Multidrug Resistance-Related Proteine
NCI	National Cancer Institute
NSAID	Non-steroidal anti-inflammatory drugs
NSAR	Nicht-steroidalen Antirheumatika
OR	Odds ratio
PP	Per-protocol
RR	Relatives Risiko
SAE	Serious Adverse Event
siRNA	Small interfering Ribonucleic acid
SOP	Standard operating procedure
SUSAR	Suspected Unexpected Serious Adverse Reaction
TGF	Transforming growth factor
ULN	Upper Limit of Normal
VEGF	Vascular endothelial growth factor
WHO	Weltgesundheitsorganisation

1. Übersicht

1.1 Zusammenfassung

Die vorliegende Studie ist eine randomisierte, doppelblinde, Placebo-kontrollierte Studie zur Prävention von metachron auftretenden kolorektalen Adenomen oder serratierten Läsionen im Kolon mit Grünteeextrakt.

Eingeschlossen werden Menschen mit kompletter Koloskopie innerhalb der letzten sechs Monate, bei denen kolorektale Adenome oder serratierte Läsionen festgestellt und komplett entfernt wurden. Eine histologische Sicherung der Diagnose Adenom muss erfolgt sein. Nach einer einmonatigen Run-In Phase erhalten die Teilnehmer an der Studie über drei Jahre randomisiert und kontrolliert entweder 300 mg/d Katechine aus Grünteeextrakt in Kapselform oder ein entsprechendes Placebo. Dokumentiert wird jeweils über einen 4-Monatszeitraum die Einnahme weiterer, potentiell chemoprotektiver Substanzen (Aspirin, andere NSAIDs), unerwünschte Ereignisse, chronische Erkrankungen, sowie der durchschnittliche Konsum an Grüntee.

Vor und nach dem Run-In, nach 4, 12, 20 und 36 Monaten erfolgt eine Kontrolle der Leberparameter (AST, ALT, Bilirubin) im Blut. Eine Kontrollkoloskopie erfolgt im Abstand von 3 Jahren, kann nach Maßgabe des untersuchenden Arztes jedoch auch früher erfolgen. Das Wiederauftreten von Adenomen, serratierten Läsionen oder ggf. Adenokarzinomen wird histologisch dokumentiert. Bei den Koloskopien werden Größe und Lokalisation aller Adenome oder sonstiger Schleimhautläsionen im Befund erhoben und die histologischen Befunde von Gewebsbiopsien und Adenomabtragungen werden dokumentiert.

Primärer Zielparameter ist das Wiederauftreten von Kolonadenomen innerhalb des Beobachtungszeitraumes von 3 Jahren. Sekundäre Zielparameter sind Zahl, Größe, Lokalisation und die histologischen Subtypen der Adenome bzw. Schleimhautläsionen und die Zeit bis zum Wiederauftreten von Adenomen sowie das Auftreten und die Frequenz schwerer Dysplasien und Karzinome in beiden Gruppen.

Fallzahlschätzung: Im Screening sind ca. 4000 Probanden mit Kolonadenomen erforderlich, um 2534 Probanden zu randomisieren. Von diesen 2534 werden von 2406 Probanden Daten zur Koloskopie erwartet, 2028 Probanden sollten die Einnahme erfolgreich beendet haben

und für die Auswertung per Protocol zur Verfügung stehen. Ein begleitendes translationales Forschungsprojekt ist vorgesehen.

1.2 Synopse

Studientitel	Minimizing the Risk of Metachronous Adenomas of the Colorectum with Green tea Extract
Kurzbezeichnung	MIRACLE-Studie
Studienleitung	Prof. Dr. med. Thomas Seufferlein<br>Direktor der Universitätsklinik und Poliklinik für Innere Medizin I, Martin-Luther-Universität Halle-Wittenberg
Studienkoordination	Prof. Dr. med. Julia Stingl<br>Sektion Klinische Pharmakologie, Institut für Naturheilkunde und Klinische Pharmakologie, Universität Ulm
Förderung / Finanzierung	Gefördert durch die Deutsche Krebshilfe e. V.
Studiendesign	Zweiarmige, doppelblinde, randomisierte, stratifizierte, multizentrische, Placebo-kontrollierte Parallelgruppenstudie
Geplante Probandenzahl	4000 Teilnehmer im Screening, 2941 Teilnehmer Run-In, 2406 Teilnehmer im ITT- und 2028 auswertbare Teilnehmer im PP-Kollektiv
Studienpopulation	Probanden nach Koloskopie und Adenomentfernung mit histologisch gesichertem Befund
Therapien	Grünteeextrakt in Kapseln, à 150mg, Dosierung 300mg Katechine pro Tag oder Placebo; tägliche Einnahme über 3 Jahre Verum versus Placebo
Geplanter Studienbeginn	September 2011
Studiendauer (klinischer Teil) pro Proband	3 Jahre

Geplante Gesamtstudiendauer	6,5 Jahre
Primäre Zielgröße	Inzidenz metachroner kolorektaler Adenome während eines Beobachtungszeitraumes von maximal 3 Jahren
Sekundäre Zielgrößen	• Zahl, Lokalisation, Größe und histologische Subtypen der Adenome bzw. Schleimhautläsionen (tubulär, villös, tubulovillös, serratiert, schwere Dysplasie, invasives Wachstum) sowie die Zeitspanne bis zum Wiederauftreten von Adenomen bei Koloskopiefrequenz < 3 Jahren.<br>• Frequenz schwerer Dysplasien und Karzinome in beiden Gruppen.<br>• Explorative Auswertungen zur genetischen, biochemischen und metabolomischen Suszeptibilität für Adenomentstehung (Zusatzprojekt „Biomarker und Nutrigenetik“).<br>• Immunhistochemische und genetische Untersuchungen der histologischen Gewebeproben von Probanden mit fortgeschrittenen Polypen (hochgradige Dysplasie oder serratierte Adenome) hinsichtlich häufig in der Adenom/Karzinomentstehung vorkommender Gewebemerkmale (K-ras-Mutation, B-raf-Mutation, spezifische micro-RNAs) (Zusatzprojekt „Histologische Gewebebank“)<br>• Untersuchung der Nutrikinetik und Nutrigenetik von Grünteeextrakt (EGCG und dessen Metabolite) bei einer Stichprobe (n=100 gesunde Probanden, Zusatzprojekt „Nutrikinetik von Epigallokatechingallat (EGCG) in oraler Formulierung: Populationsvariabilität und Nutrigenomik“).

	• Einfluss der Meinung zu „alternativen Heilmethoden“ der Probanden (Fragebogen) auf die primären und sekundären Zielgrößen und die Compliance
Einschlusskriterien	• Teilnehmer/Innen im Alter von 50-80 Jahren mit Koloskopie innerhalb der letzten 6 Monate und nach Entfernung histologisch gesicherter kolorektaler Adenome oder serratierter Läsionen<br>• Allgemeinzustand nach ECOG-Skala (Eastern Cooperative Oncology Group Performance status scale) < 2<br>• Schriftliche Einwilligung in die Studie
Ausschlusskriterien	• Bekannte Familienanamnese für Hereditäres kolorektales Karzinom ohne Polyposis (HNPCC) oder Familiäre adenomatöse Polyposis (FAP)<br>• Anamnestisch oder gegenwärtiges Bestehen eines kolorektalen Karzinoms<br>• gegenwärtiges Bestehen anderer Tumorerkrankungen mit Ausnahme von kurativ erfolgreich behandelten Tumoren ohne aktuelle Medikation sowie Basaliomen<br>• Malabsoptionssyndrom, Kurzdarmsyndrom und verkürzende chirurgische Interventionen (Ileostomie), die die Resorption beeinträchtigen können<br>• Schwere Lebererkrankung (Hepatitis, Leberzirrhose > Child A, Erhöhung von ALT und AST > 2,5-faches der Norm, Erhöhung von Bilirubin > 2,5 -faches der Norm)<br>• Chronisch entzündliche Darmerkrankungen<br>• Regelmäßige Einnahme von NSAR oder COX-2 Hemmern über mehr als drei Monate pro Jahr (Ausnahme: Einnahme von niedrigdosiertem ASS bis 100 mg/d)

	• Regelmäßige Einnahme immunsuppressiver Medikation<br>• Teilnehmer, die die Kapseln nicht schlucken können<br>• Teilnehmer, deren Fähigkeit zur Einwilligung in die Studie eingeschränkt ist<br>• Bekannte Allergie auf Grünen Tee<br>• Regelmäßiger Konsum von Grünteeextrakt (mit EGCG Gehalt von >100mg) über ein halbes Jahr oder mehr in den letzten 2 Jahren
Biometrie	Prof. Dr. Rainer Muche,<br>Institut für Biometrie, Universität Ulm
Studienzentren	20-30 gastroenterologische Schwerpunktpraxen und zertifizierte Darmkrebszentren bundesweit; Klinik für Innere Medizin I, MLU Halle (Saale)

1.3 Flow Chart

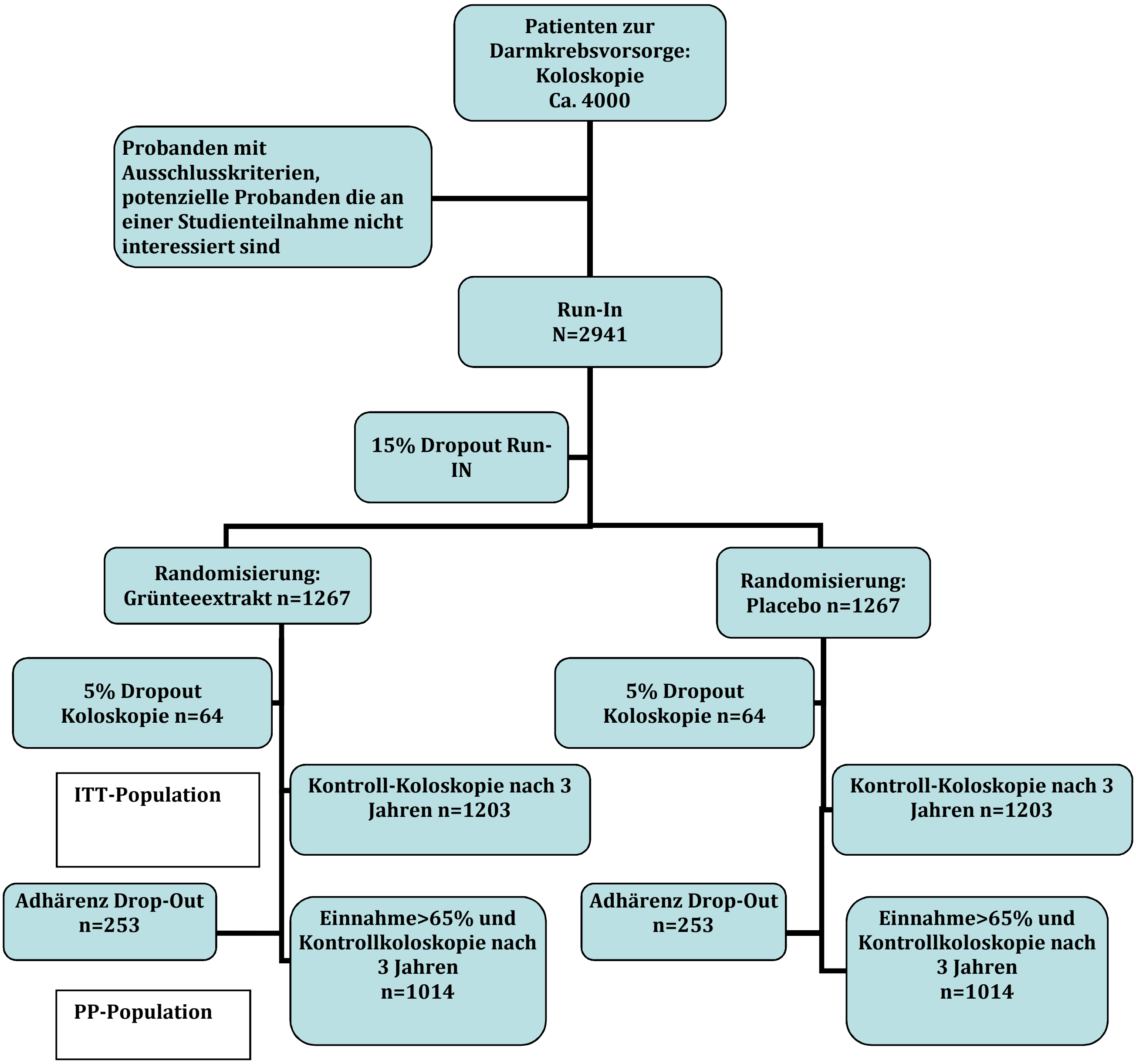

1.4 Ausgangssituation

Grüner Tee, aus den Blättern der Pflanze Camellia Sinensis hergestellt, gehört zu den ältesten und weltweit populärsten Getränken. Die Inhaltstoffe von Grünem Tee zeigen neben positiven Wirkungen auf das kardiovaskuläre System [1,2] auch präventive Wirkungen auf das Tumorwachstum bei Krebserkrankungen [3-5].

Die wirksamen Inhaltsstoffe von Grünem Tee sind die Polyphenole, gerbstoffartige Substanzen, die antioxidative, antimutagene und antiinflammatorische Eigenschaften besitzen [3]. Grünteeextrakt enthält zu etwa 30 % Polyphenole, insbesondere monomere

Flavan-3-ole, auch Catechine genannt [6]. Die vier wichtigsten, in Grüntee enthaltenen Catechine sind (-)-Epigallocatechin-3-gallat (EGCG), welches etwa 50 % des Catechingehaltes ausmacht, (-)-Epigallocatechin (EGC, 10-20 % des Catechingehaltes) und (-)-Epicatechin-3-gallat (ECG, 10-20 % des Catechingehaltes) sowie (-)-Epicatechin (EC, das 5-10 % des Catechingehaltes) [7].

Eine Tasse Grüntee (2,5g Blätter in 200ml Wasser) enthält etwa 300-400mg Polyphenole, davon 20-50mg EGCG (Ullmann et al. 2004). Kommerziell erhältliche Grünteeextrakt-Präparate enthalten zwischen 60mg und 150mg EGCG pro Kapsel (z.B. Loges Praevent®, Vcaps®, Grüntee Kapseln Kräuterhaus St. Bernhard).

1.4.1 In vitro Untersuchungen zu antiproliferativen Effekten von Grüntee-Katechinen

Grünteeextrakt und EGCG wurden in einer Vielzahl von molekularen Untersuchungen, Tierversuchen und auch in zahlreichen Studien am Menschen hinsichtlich ihrer Krebs-präventiven Eigenschaften untersucht. Die molekularen Wirkweisen sind vielfältig: Induktion von Apoptose in Krebszellen über Proteasomen-Hemmung sowie über TGF-β [8,9], Hemmung der Aktivierung von Tyrosinkinasen (EGFR, HER2 und -3) [10], Inhibition der Aktivität von Topoisomerase I [11], sowie Hemmung der VEGF-induzierten Angiogenese [12]. Auch eine Hemmung der COX-2 Expression in Krebszellen wurde für EGCG nachgewiesen [13]. Die COX-2 Hemmung wird für den chemoprotektiven Effekt von Aspirin oder selektiven COX-2 Hemmstoffen hinsichtlich der Entstehung von Kolonadenomen verantwortlich gemacht.

Die Wirkung von Katechinen in Zellen wird über ihre Bindung an den Lamininrezeptor an der Plasmamembran vermittelt [14]. Es handelt sich um einen transmembranösen Rezeptor von 67 kDa, der intrazellulär über ein G-Protein (eEF1A) zur Aktivierung einer Myosinphosphatase führt (Abb. 1 aus [14]). Eine Inaktivierung des Lamininrezeptors (LR-67) mittels siRNA führt zu einem Verlust des proapoptotischen Effektes von EGCG auf Myelomzellen [15].

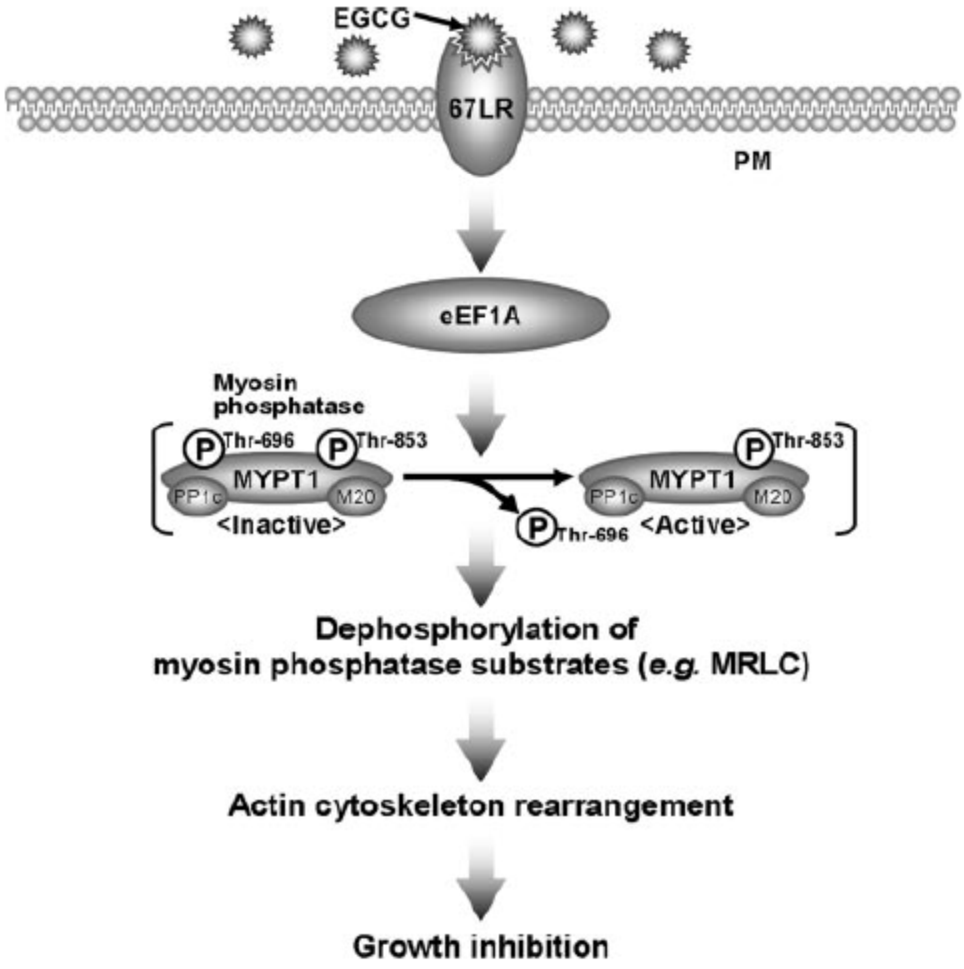

Abbbildung 1:

Darstellung der intrazellulären Effekte der Bindung von EGCG am Laminin-rezeptor[14].

1.4.2 In vivo Untersuchungen zu chemoprotektiven Eigenschaften von Grüntee-Katechinen

Die Gabe von Grünteeextrakt im Trinkwasser führt in Tiermodellen zu einer verminderten Karzinogenese. So reduziert eine Grünteegabe z.B. bei Ratten die Zahl präneoplastischer Läsionen nach Azoxymethan-Exposition [16,17]. Im Apc^{min} Mausmodell verhindert Grüntee die Entstehung von Kolonadenomen [18,19].

1.4.3 Klinische Daten

Epidemiologische Studien weisen auf eine präventive Wirkung von Grüntee auf die Entstehung von Kolonkarzinomen auch beim Menschen hin [20-22]. Eine 2006 durchgeführte Metaanalyse zeigt, dass allerdings Faktoren wie Studiendesign, Geschlecht der Probanden

und Ethnizität das Ergebnis der epidemiologischen Studien beeinflussen [23]. Vor allem scheint die Menge an Grüntee, die über die Zeit konsumiert wird, eine Rolle für die anti-Tumor Wirkung des Tees zu spielen. In den Tierstudien wurde neben fester Nahrung ausschließlich Grünteeextrakt, aufgelöst im Trinkwasser, verabreicht, was verglichen mit gebrühtem Grünem Tee in den epidemiologischen Studien wahrscheinlich zu einer gleichmäßigeren und höheren Exposition führt.

In Abb. 2 sind die Ergebnisse der epidemiologischen Studien zur Risikoreduktion von Kolonkarzinomen durch Trinken von Grünem Tee dargestellt (aus [23]).

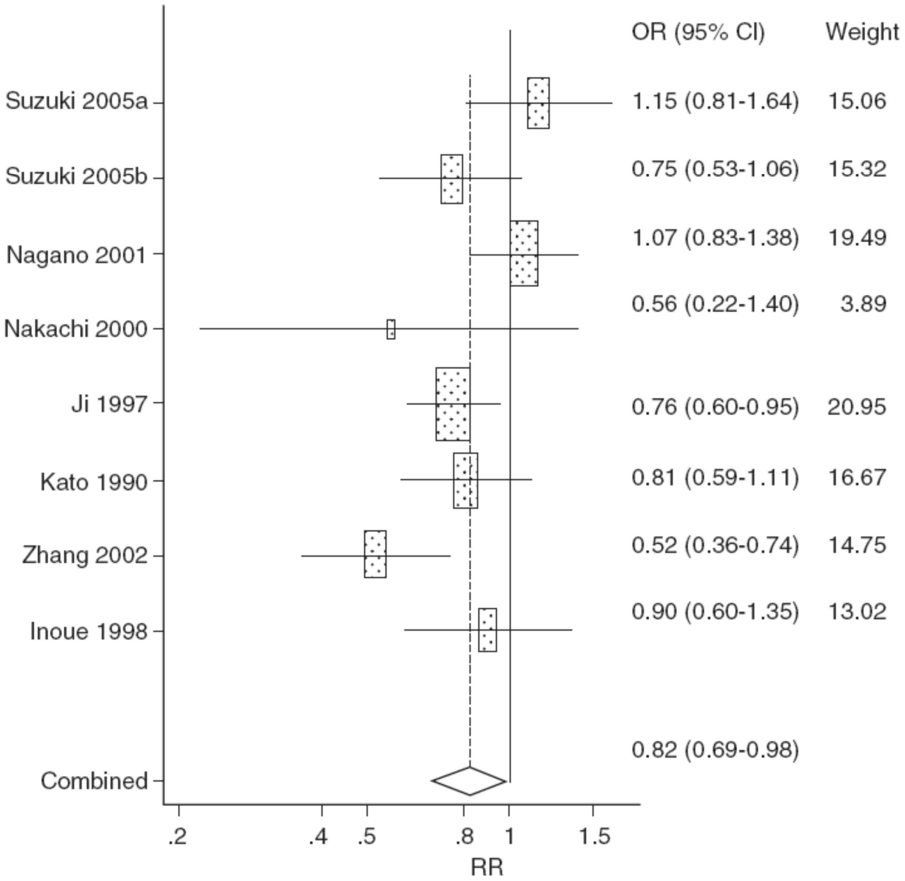

Abbildung 2: Metaanalyse zu kolorektalem Krebsrisiko und Einnahme von Grünem Tee [23].

Derzeit gibt es eine kleine Anzahl prospektiver kontrollierter Studien zur Krebsprävention mit Grünem Tee. In diesen Studien wurden definierte Mengen an Katechinen aus Grünteeextrakt verwendet. So konnten positive Effekte der Polyphenole für die Prävention von Prostatakarzinomen [24], Hautkrebs, Karzinomen des Mund- und Rachenraums [25,26], hepatozellulärem Karzinom [27] und Cervixkarzinom [28] sowie des asymptomatischen Rai

Stadiums einer chronisch lymphatischer Leukämie [5] gezeigt werden. Derzeit sind 6 Phase II Studien zu Polyphenon E (von der Japanischen Firma Mitsui Norin produzierter Grünteeextrakt mit definierten Anteilen an Katechinen) in der Prävention am National Cancer Institute in den USA zu unterschiedlichen Tumorentitäten (Lungenkrebs, Brustkrebs, Cervix-Karzinom, chronisch lymphatische Leukämie und Ösophaguskarzinom) registriert und befinden sich in der Durchführung. In diesen prospektiven Studien werden vergleichsweise hohe Mengen Grünteeextrakt oral verabreicht. So wurde in der Studie zur Prävention von Prostatakarzinomen 600 mg Grünteekatechine (ECGC-Gehalt etwa 50 %) pro Tag verabreicht, was etwa 7 Tassen Grüntee oder 1,4 Litern entspricht. Zum Vergleich: Im APC^{min}-Mausmodell wurden 0,6 %ige Teeextraktlösungen, was einer ECGC Menge von etwa 180 mg in 250 ml Flüssigkeit entspricht, verwendet [19]. Allerdings war diese Teeextraktlösung die einzige Trinkquelle für die Tiere.

1.4.4 Grüntee zur Prävention kolorektaler Adenome

Zur Sekundärprävention von Kolonadenomen und zur Prävention von Kolonkarzinomen nach Polypektomie liegen eine Reihe von prospektiven Studien zu nichtsteroidalen Antirheumatika/Aspirin und COX2-Hemmern (Rofecoxib, Celecoxib) vor[29-34]. Grünteekatechine mit ihren COX2-inhibierenden und chemoprotektiven Eigenschaften könnten daher eine interessante Option zur Chemoprävention darstellen. Eine, prospektive Pilotstudie an 125 Patienten zeigte, dass im Vergleich zur Kontrollgruppe eine Supplementation der Ernährung mit Grünteeextrakt zu einer Halbierung der Inzidenz metachroner Adenome nach 1 Jahr führte (15% vs. 31%, 95% KI: 0.24-0.99, $p<0.05$) [35]. Diese Daten sind vielversprechend, insbesondere wenn man das Nebenwirkungsspektrum von Medikamenten mit COX2-inhibierenden Eigenschaften mit denen von Grünteeextrakt vergleicht. Allerdings wurden diese Daten in einem offenen Studiendesign mit einer relativ geringen Fallzahl gewonnen. Für den wirklichen Nachweis der chemopräventiven Wirksamkeit von Grüntee ist ein kontrolliertes, verblindetes, randomisiertes Studiendesign notwendig.

1.4.5 Kinetische Untersuchungen zu Grüntee-Katechinen beim Menschen

Bisher ist unklar, ob Grüntee und Grünteeextrakt vorwiegend lokal auf die Darmmukosa wirken (aufgrund biliärer Exkretion) oder auch systemisch wirksam sind.

Resorption, Verteilung und Ausscheidung von Grüntee wurde für die Hauptkatechine Epigallocatechin-3-Gallat, Epigallocatechin und Epicatechin beim Menschen nach Einmalgabe sowie nach Mehrfachgaben von Grüntee-Kapseln untersucht. Epigallocatechin-3-Gallat wird nach oraler Einnahme (Kapsel) schnell absorbiert und erreicht maximale Konzentrationen im Blut nach etwa 1-2 Stunden. Nach Einmaldosis einer 400 mg EGCG Kapsel wurden maximale Plasmakonzentrationen von 112 ng/ml und eine AUC von 623 ng/ml*h gemessen, nach Einmaldosis von 800 mg EGCG wurden 438 ng/ml und eine AUC von 2779 ng/ml*h gemessen [36]. Die Halbwertszeit im Plasma von EGCG liegt bei 2-4h. Die Einnahme von 400 mg und 800 mg über 4 Wochen führte zu keiner Akkumulation (die Predose-Werte waren negativ, auch bei multipler Dosierung), da die Halbwertszeit zu kurz ist. Dennoch lag die AUC bei der 800 mg Dosis 60 % höher, was möglicherweise an einer Hemmung des präsystemischen intestinalen Metabolismus liegt. Bei einer Aufteilung von 800 mg/d in mehrere Einzeldosen ist damit mehr EGCG im Blut nachweisbar als bei einer täglichen Einmaldosis [37-39]. EGCG liegt im Plasma vorwiegend ungebunden (92%) vor, wird vorwiegend über die Galle intestinal ausgeschieden und im Urin weder unkonjugiert noch in der glukuronidierten Form nachgewiesen [37-39].

In Abbildung 3 ist der Zeitverlauf über 24 h nach Einnahme einer 800 mg- bzw. 400 mg-haltigen Kapsel Epigallocatechin-3-Gallat dargestellt. Die runden Markierungen zeigen den Verlauf nach einer einmaligen Einnahme („before“), die dreieckigen Markierungen zeigen den Verlauf nach 4-wöchiger Einnahme („after“).

Derzeit sind in der Datenbasis für Klinische Studien des NIH (www.clinicaltrials.gov) 55 klinische Studien zu Grünteeextrakt aufgeführt (Stand 03/09). Von diesen 55 Studien sind 26 Studien zur Behandlung von Krebserkrankungen und 7 Studien explizit zur Prävention maligner Erkrankungen konzipiert. Die verwendeten Dosierungen (Gehalt an EGCG) in den Präventionsstudien liegen zwischen 200 mg und 800 mg täglich. In einer publizierten Studie zur Chemoprävention (Sekundärprävention von Prostatakarzinomen) wurden 600 mg Gesamtkatechine mit einem Gehalt an EGCG von 51 % pro Tag verabreicht [24]. In der Pilotstudie zur Prävention metachroner kolorektaler Adenome wurden 1,5 g Grünteeextrakt, jedoch mit einem relativ niedrigen Anteil an EGCG von 158 mg sowie einem Gesamtkatechingehalt von 333 mg pro Tag verabreicht [35].

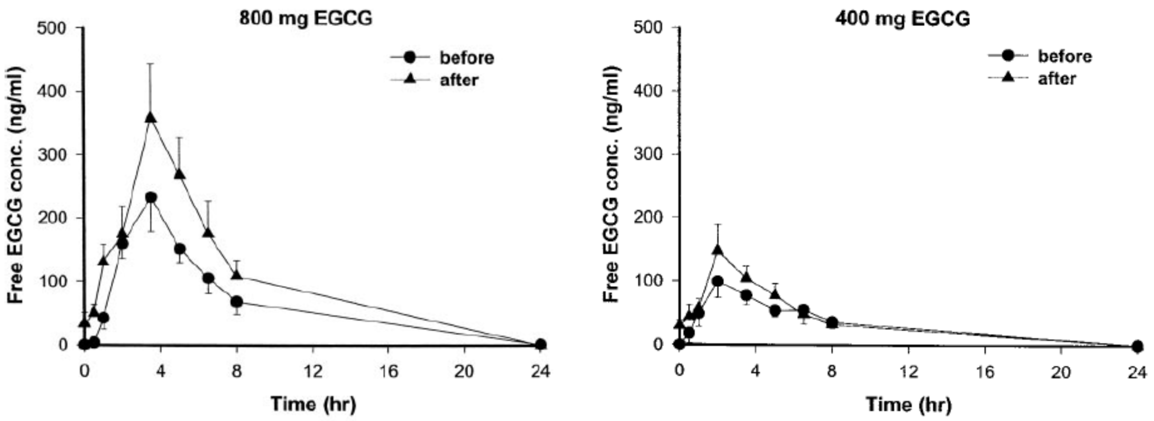

Abbildung 3: Plasma-Konzentrationsverläufe von EGCG nach Einmaldosis von 800 mg bzw. 400 mg in Kapselform nach einmaliger (Kreise) bzw. nach vierwöchiger (Dreiecke) Einnahme.[37]

1.5 Fragestellung und Begründung des Vorhabens

Darmkrebs ist bei Männern und Frauen die zweithäufigste Krebserkrankung in Deutschland. Zur wirksamen Prävention kolorektaler Karzinome wurde 2003 das Koloskopiescreening in Deutschland eingeführt, an dem mittlerweile mehrere Millionen Menschen teilgenommen haben. Bei etwa einem Drittel der Untersuchten werden Adenome oder serratierte Läsionen gefunden. Im Vergleich zur Normalbevölkerung besteht bei diesen Patienten ein erhöhtes Risiko für ein erneutes Auftreten von Polypen. Daher wird empfohlen, zumindest bei Patienten mit 3 - 10 Adenomen, oder mindestens einem Adenom, das 1 cm oder größer ist, oder einem Adenom mit villöser Histologie die erste Kontrollkoloskopie nach 3 Jahren durchzuführen. Bei Patienten mit 1 oder 2 Adenomen < 1 cm ohne höhergradige intraepitheliale Neoplasie dürfte eine Kontrollkoloskopie nach 5 Jahren ausreichend sein (S3-Leitlinie Kolorektales Karzinom: Prävention, Diagnostik und Therapie, Deutsche Gesellschaft für Verdauungs- und Stoffwechselkrankheiten DGVS, 2008, [40]). Für beide Patientengruppen ist eine suffiziente, d.h. effektive, aber nebenwirkungsarme Sekundärprophylaxe wünschenswert. Eine medikamentöse Sekundärprävention, u.a. mit Aspirin, anderen NSARs oder COX2-Hemmstoffen wird von der aktuellen S3 Leitlinie nicht empfohlen. Dies liegt an der ungünstigen Nutzen-Risiko-Relation. Nebenwirkung wie z.B. gastrointestinale Blutungen bei Aspirin und kardiovaskuläre Nebenwirkungen bei COX2- Hemmern wiegen den potenziellen Nutzen der medikamentösen Sekundärprävention in der Normalbevölkerung

auf [40]. Durch die aktuellen Konzepte kann daher der Bedarf an einer nebenwirkungsarmen, effektiven Sekundärprävention von Darmpolypen nicht gedeckt werden. Die Wirksamkeit von Grüntee zur Prävention von unterschiedlichen Krebsarten ist in der Literatur gezeigt. Im Tiermodell wurde in mehreren Studien eine den NSAIDs vergleichbare präventive Wirkung von Grünteeextrakt, bzw. Epigallokatechingallat, auf die Entstehung von Darmpolypen gezeigt. Unter www.clinicalTrials.gov sind derzeit 55 Klinische Studien zu Grünteeextrakt sowie seinem wirksamen Hauptbestandteil Epigallokatechingallat registriert. Die publizierten Daten zur Prävention von Prostatakarzinomen und Cervixkarzinomen bei Hochrisikopatienten, sowie eine Pilotstudie zur Prävention metachroner Kolonadenome mit Grünteeextakt sind sehr vielversprechend [24 28 35].

In der vorliegenden Studie soll die Möglichkeit einer Prävention metachroner Kolonadenome durch Einnahme von Grünteeextrakt evaluiert werden. Aufgrund des bisherigen Fehlens einer Standardprävention im zu untersuchenden Kollektiv (Teilnehmer an einer Vorsorgekoloskopie mit komplett entfernten Kolonadenomen) wird diese Intervention mit Placebo verglichen [41].

1.6 Abschätzung der Übertragbarkeit der erwarteten Studienergebnisse auf die Praxis

Die Fallzahl der Studie wurde dahingehend geplant, dass klinisch relevante Unterschiede in der präventiven Wirkung von Grünteeextrakt in der Größenordnung der Ergebnisse der chemopräventiven Studie zu Aspirin[30] erkannt werden können. Unterschiede in dieser Größenordnung würden eine Einnahme von Grünteeextrakt in der breiten Bevölkerung und über längere Zeit rechtfertigen. Im Falle einer Bestätigung der Hypothese, dass Grünteeextrakt beim Menschen eine relevante chemopräventive Wirkung auf die Inzidenz metachroner Kolonadenome hat, könnten die Ergebnisse direkt in die klinische Praxis übertragen werden. Bei 1000 Studienteilnehmern, die den Grünteeextrakt über drei Jahre einnehmen, können ausreichende Erfahrungen mit diesem Extrakt gewonnen werden. Eine Extrapolation, dass auch das Trinken von Grüntee eine vergleichbare chemopräventive Wirkung hat, wäre aus den Daten nicht ohne weiteres möglich, da Grüntee in Wirkstoffgehalt, Koffeingehalt und Resorption je nach Zubereitung sehr variiert und daher

eine gleichmäßige Dosierung von Epigallokatechingallat, das als der hauptwirksame Bestandteil von Grüntee gilt, nicht ausreichend gegeben ist.

1.7 Primäres Studienziel

Inzidenz metachroner kolorektaler Adenome oder serratierter Läsionen in der Gruppe, die Grünteeextrakt erhält, im Vergleich zur Placebogruppe bei der Follow-up Koloskopie 3 Jahre nach der initialen Polypektomie.

1.8 Sekundäre Ziele

- Zahl, Lokalisation, Größe und histologische Subtypen der Adenome bzw. Schleimhautläsionen (tubulär, villös, tubulovillös, serratiert, schwere Dysplasie, invasives Wachstum) sowie die Zeitspanne bis zum Wiederauftreten von Adenomen bei Koloskopiefrequenz < 3 Jahren.
- Frequenz schwerer Dysplasien und Karzinome in beiden Gruppen.
- Explorative Auswertungen zur genetischen, biochemischen und metabolomischen Suszeptibilität für Adenomentstehung (Zusatzprojekt „Biomarker und Nutrigenetik").
- Immunhistochemische und genetische Untersuchungen der histologischen Gewebeproben von Probanden mit fortgeschrittenen Polypen (hochgradige Dysplasie oder serratierte Adenome) hinsichtlich häufig in der Adenom/Karzinomentstehung vorkommender Gewebemerkmale (K-ras-Mutation, B-raf Mutation, spezifische micro-RNAs) (Zusatzprojekt „Histologische Gewebebank")
- Untersuchung der Nutrikinetik und Nutrigenetik von Grünteeextrakt (EGCG und dessen Metabolite) bei einer Stichprobe (n=100 gesunde Probanden, Zusatzprojekt „Nutrikinetik").
- Einfluss der Meinung zu „alternativen Heilmethoden" der Probanden (Fragebogen) auf die primären und sekundären Zielgrößen und die Compliance

1.9 Wissenschaftliches Begleitprogramm

1.9.1 Zusatzprojekt „Biomarker und Nutrigenetik"

Dieses wissenschaftliche Begleitprojekt umfasst explorative Auswertungen zur genetischen, biochemischen und metabolomischen Suszeptibilität für Adenomentstehung.

Dabei sollen bei Studienteilnehmern mit fortgeschrittenen Polypen, schweren Dysplasien/ oder Karzinomen bei der 3-Jahres-Kontrolle genomweite Untersuchungen zu genetischen Polymorphismen sowie Kandidatengenuntersuchungen zu bekannten funktionellen Genloci durchgeführt werden.

Da es für Epigallokatechingallat bekannte pharmakogenetische Polymorphismen in der Biotransformation (COMT, SULT, UGT) oder auch für den Transport in die Zelle (z.B. MRP1,2) gibt, soll die Bedeutung dieser Polymorphismen für die präventive Wirksamkeit in einem Kandidatengen-Ansatz geprüft werden.

1.9.2 Zusatzprojekt „Histologische Gewebebank“

Es sind immunhistochemische und genetische Untersuchungen der histologischen Gewebeproben von Probanden mit fortgeschrittenen Polypen (hochgradige Dysplasie oder serratierte Adenome) hinsichtlich häufig in der Adenom/Karzinomentstehung vorkommender Gewebemerkmale (K-ras-Mutation, B-raf-Mutation, spezifische micro-RNAs) geplant.

1.9.3 Zusatzprojekt „Nutrikinetik von Epigallokatechingallat (EGCG) in oraler Formulierung: Populationsvariabilität und Nutrigenomik“

Im Rahmen dieser Studie soll zusätzlich an einer unabhängigen Kohorte von n = 100 gesunden Probanden eine Untersuchung zur multiple dose Kinetik von EGCG 2x150 mg/d durchführt werden. Dafür werden gesondert ein Studienplan, Einverständniserklärungen und Probandenaufklärungen verfasst und eine gesonderte Zustimmung der zuständigen Ethikkommission eingeholt.

Diese Untersuchungen sind teilweise Gegenstand separater Projekte, für das durch diese Studie die Voraussetzungen geschaffen werden.

2 Allgemeine Angaben zur Studie

2.1 Art der Studie / Studiendesign

Es handelt sich um eine Studie zur Chemoprävention mit einem Nahrungsergänzungsmittel (Dietary supplement).

Das Design stellt eine zweiarmige, randomisierte, stratifizierte, doppelblinde, Placebo-kontrollierte Studie im Parallelgruppendesign zur chemoprotektiven Wirkung von Grünteeextrakt auf die Inzidenz metachroner kolorektaler Adenome oder serratierter Läsionen dar. Es wird eine 1-monatige Run-In-Phase mit Verum vorangestellt.

2.2 Zahl und Art der Vergleichsgruppen

Es werden zwei gleich große Gruppen verglichen: Placebo versus Grünteeextrakt. Die Gruppen werden nach Randomisierung parallel gebildet. Zusätzlich erfolgt eine Stratifizierung hinsichtlich der Einnahme von ASS (≤ 100 mg/d; ≥ 100 mg/d ist ein Ausschlußkriterium).

2.3 Umfang der Studie / Machbarkeit

Die Studie wird multizentrisch in gastroenterologischen Schwerpunktpraxen und in zertifizierten Darmkrebszentren sowie an der Klinik für Innere Medizin I, MLU Halle (Saale) durchgeführt.

Die Probandenzahl (Intention-to-treat Population) umfasst 1203 auswertbare Probanden pro Gruppe, was bei einer angenommenen Drop-Out Rate von 15% in der Run-In Periode und 5% für die Folgekoloskopie einen Einschluss von 2941 Teilnehmern in die Run-In Periode und 2534 in die Randomisation erfordert. Die per-Protocol Population umfasst unter Annahme von 20% Dropout dann 1014 Teilnehmer pro Gruppe (s. 12.2). Es wird erwartet, dass ca. 4000 Patienten nach Polypektomie im Rahmen einer Koloskopie angesprochen werden müssen, um 2941 Teilnehmer in die Run-In Periode einladen und davon 2534 randomisieren zu können. Genauere Angaben zur Fallzahlplanung finden sich im entsprechenden Abschnitt.

In Deutschland werden jährlich ca. 500 000 Menschen im Rahmen einer Vorsorgekoloskopie untersucht. Eine große gastroenterologische Schwerpunktpraxis bzw. ein großes Darmkrebszentrum führt im Jahr etwa 3000-7000 Koloskopien durch, davon sind ca. 10-15% Koloskopien mit Entfernung von Polypen. Von diesen sind bei etwa der Hälfte der Untersuchten die Einschlußkriterien der Studie erfüllt. Es sollen 20-30 gastroenterologische Schwerpunktpraxen bzw. zertifizierte Darmkrebszentren sowie die Klinik für Innere Medizin I der MLU Halle (Saale) in diese Studie einbezogen werden. Diese Zahl an Studienzentren

ermöglicht es, die Rekrutierung der geplanten 2941 Probanden in spätestens drei Jahren durchzuführen.

2.4 Probandenrekrutierung

Die Probandenrekrutierung erfolgt über die klinischen Partner. Die potentiellen Teilnehmer werden nach Überprüfen der Einschlusskriterien von den behandelnden Ärzten über die Studie informiert. Die Studie wird bundesweit bekannt gemacht, um einen hohen Bekanntheitsgrad zu erzielen.

Vor dem eigentlichen Beginn der Studie wird eine einmonatige Verum-Run-In Phase durchgeführt, während der alle Studienteilnehmer pro Tag 2 Kapseln Grünteeextrakt einnehmen. Nach der einmonatigen Run-In Periode beginnt die randomisierte Zuteilung von Studiensubstanz bzw. Placebo. Das KKS Halle koordiniert die Ausgabe der Studienware an die klinischen Partnern über den Logistikpartner LOXXESS, der die Lagerung und Distribution der Studienware (70 Kapseln Verum oder Placebo pro Monat, verblistert in Faltschachteln, jeweils für einen Zeitraum von 4 Monaten abgepackt) durchführt. Die Prüfärzte stellen sicher, dass die Studiensubstanz rechtzeitig und in ausreichender Menge an die Probanden ausgehändigt wird und dass gleichzeitig die Dokumentation über die vergangenen 4 Monate durchgeführt und an das KKS Halle weitergeleitet wird. Dies geschieht in Form eines CRFs und eines Probandentagebuchs, das jeweils über den Zeitraum von 4 Monaten geführt wird. Die Informationen des Probandentagebuchs werden von den Prüfzentren erfasst und fließen in den CRF mit ein. Die Probandentagebücher werden in Sammelversendungen alle 4 Monate von den Prüfzentren an das KKS Halle gesendet.

2.5 Randomisierungsprinzip

Die Studienteilnehmer werden in die beiden Gruppen (Grünteeextrakt und Placebo) durch das Institut für Biometrie der Universität Ulm randomisiert (stratifizierte Blockrandomisierung, Software ROM). Es wird nach Studienzentrum und Einnahme von niedrigdosiertem ASS (≤100 mg/d ja/nein) stratifiziert. Eine weitere Stratifizierung erfolgt nicht, da angenommen wird, dass in den Studienarmen mit jeweils 1203 Probanden im ITT-Kollektiv weitere systematische Einflussfaktoren, wie Grüntee-Konsum, Alter, Geschlecht, Familienanamnese bezüglich Darmkrebs, und andere potentiell präventiv wirkende Medikation und Lebensweise gleich verteilt auftreten. Es wird erwartet, dass die

teilnehmenden Zentren mindestens 30 Probanden pro Jahr rekrutieren. Die Rekrutierung ist kompetitiv.

2.6 Zeitplan

	2011/12	2012	2013	2014	2015	2016	2017
Aktivierung und Vorbereitung							
Rekrutierung							
Studienvisits							
3-Jahres-Follow-up							
Auswertung							
Überprüfung von Probandensicherheit, Datenqualität und Rekrutierung							

Beginn der Studie: 2011.

Ende der Rekrutierungsphase (voraussichtlich): 3 Jahre nach Rekrutierungsbeginn.

Ende der Nachbeobachtungsphase (voraussichtlich): 6 Jahre nach Studienbeginn.

Dauer der Studie pro Proband: 3 Jahre.

2.7 Zeitliches Studienablaufschema

Prüfung	Screening	Run-In-Phase		Studienphase								
	Monat -6 - 0	Baseline	Ende Monat 1	Ende Monat 4	Ende Monat 8	Ende Monat 12	Ende Monat 16	Ende Monat 20	Ende Monat 24	Ende Monat 28	Ende Monat 32	Ende Monat 36
Koloskopie mit Polypektomie (Adenom)	x											
Eignung zur Teilnahme (Eligibility)	x											
Einwilligungserklärung	x											
Registrierung		x										
Vorerkrankungen, Dauermedikation incl. ASS, COX2-Hemmer, Statine		x										
Fragebogen: „Alternative Heilmethoden“		x										
Basisdaten (Größe, Gewicht, etc.)		x										
Ausgabe der Studienmedikation		x	x	x	x	x	x	x	x	x	x	
Ausgabe/Tausch der Probandentagebücher		x	x	x	x	x	x	x	x	x	x	
Unerwünschte Nebenwirkungen			x	x	x	x	x	x	x	x	x	x
Compliance-Screening			x	x	x	x	x	x	x	x	x	x
Medikamentenanamese (ASS, COX2-Hemmer, Statine)		x	x	x	x	x	x	x	x	x	x	x
Leberwerte (Bilirubin, AST, ALT)		x	x	x		x		x				x
Kontrollkoloskopie												x

Prüfung	Screening	Run-In-Phase		Studienphase								
	Monat -6 - 0	Baseline	Ende Monat 1	Ende Monat 4	Ende Monat 8	Ende Monat 12	Ende Monat 16	Ende Monat 20	Ende Monat 24	Ende Monat 28	Ende Monat 32	Ende Monat 36
Zusatzprojekt „Histologische Gewebebank“: Anforderung/Verschickung der Gewebeproben[b]			x									x
Zusatzprojekt „Biomarker und Nutrigenetik”: Blutentnahme[a]			x	x				x				x

a Nur bei Teilnahme der Probandin bzw. des Probanden am Zusatzprojekt „Biomarker und Nutrigenetik”

b Nur bei Teilnahme der Probandin bzw. des Probanden am Zusatzprojekt „Histologische Gewebebank“ und dem Vorliegen von fortgeschrittenen Polypen (high-grade Dysplasie, serratierte Adenome)

Die Studie endet regulär mit dem Vorliegen des zweiten Koloskopiebefundes nach 3 Jahren und ggf. des histopathologischen Befundes von entfernten Kolonadenomen 3 Jahre nach Einschluss des Teilnehmers.

3 Studienzentren

3.1 Teilnehmende Zentren

Bundesweit 20-30 gastroenterologische Schwerpunktpraxen und zertifizierte Darmkrebszentren, Klinik für Innere Medizin I, Universitätsklinikum der MLU Halle (Saale).

3.2 Bedingungen für die Teilnahme an der Studie

Die Studienzentren erfüllen die Voraussetzungen für die Durchführung von Koloskopien, z.B. im Rahmen der Darmkrebsvorsorge und führen diese bereits regelmäßig durch. Sie besitzen eine ausreichende Qualifikation für die Durchführung von klinischen Studien. Bei Rekrutierung von weniger als 10 Probanden/Jahr kann in einem Zentrum die weitere Rekrutierung neuer Probanden beendet werden. Zentren ohne aktive Probanden können von der Studienleitung geschlossen und durch neue Zentren ersetzt werden. Zentren mit mangelhafter Qualität der erhobenen Daten können von der Rekrutierung neuer Probanden ausgeschlossen werden. Die jeweilige Entscheidung trifft die Studienleitung.

Es muss eine histologische Aufarbeitung der bei der Koloskopie entnommenen Adenome erfolgen, die es erlaubt, die Zuordnung zu Adenom bzw. Karzinom eindeutig zu machen.

Die Prüfzentren müssen über eine ausreichende Datendokumentation verfügen, die es erlaubt On-site Monitoring-Besuche zur Source-Data Verifikation aus den Originaldaten (Probandenakten etc.) durchzuführen.

4 Auswahl der Probanden

Es werden Teilnehmer in die Studie eingeschlossen, bei denen im Rahmen einer Vorsorgekoloskopie innerhalb der letzten 6 Monate Kolonadenome oder serratierte Läsionen entdeckt und vollständig entfernt wurden.

4.1 Einschlusskriterien

- Teilnehmer/Innen im Alter von 50-80 Jahren mit Koloskopie innerhalb der letzten 6 Monate und nach Entfernung histologisch gesicherter kolorektaler Adenome oder serratierter Läsionen
- Allgemeinzustand nach ECOG-Skala < 2
- Schriftliche Einwilligung in die Studie

4.2 Ausschlusskriterien

- Bekannte Familienanamnese für hereditäres kolorektale Karzinome ohne Polyposis (HNPCC) oder familiäre adenomatöse Polyposis (FAP)
- Anamnestisch oder gegenwärtiges Bestehen eines kolorektalen Karzinoms
- gegenwärtiges Bestehen anderer Tumorerkrankungen mit Ausnahme von kurativ erfolgreich behandelten Tumoren ohne aktuelle Medikation sowie Basaliomen
- Malabsoptionssyndrom, Kurzdarmsyndrom und verkürzende chirurgische Interventionen (Ileostomie), die die intestinale Resorption beeinträchtigen können.
- Schwere Lebererkrankung (Hepatitis, Leberzirrhose > Child A, Erhöhung der Transaminasen (ALT, AST) > 2,5-fache der Norm, Erhöhung von Bilirubin > 2,5 -fache der Norm)
- Chronisch entzündliche Darmerkrankungen
- Regelmäßige Einnahme von NSAR oder COX-2 Hemmern über mehr als drei Monate pro Jahr (Ausnahme: Einnahme von niedrigdosiertem ASS bis 100 mg/d)
- Regelmäßige Einnahme immunsuppressiver Medikation
- Teilnehmer, die die Kapseln nicht schlucken können
- Teilnehmer, deren Fähigkeit zur Einwilligung in die Studie eingeschränkt ist
- Bekannte Allergie auf Grünen Tee
- Regelmäßiger Konsum von Grünteeextrakt (mit EGCG Gehalt von >100 mg) über ein halbes Jahr oder mehr in den letzten 2 Jahren

5 Aufnahme/Registrierung und Randomisation

5.1 Enrolment und Run-In

Die Aufklärung jedes Probanden erfolgt durch ein Gespräch zwischen dem Studienarzt des Studienzentrums und dem Probanden vor Einschluss in die Studie. Der Proband erhält ausreichend Bedenkzeit und Gelegenheit für Rückfragen. Nach Beantwortung aller Fragen des Probanden und nach schriftlicher Einwilligungserklärung kann eine Aufnahme in die Studie erfolgen. Die Probanden müssen innerhalb des Zeitraumes von 6 Monaten nach Koloskopie und Adenomentfernung in die Studie eingeschlossen werden.

Alle Teilnehmer durchlaufen eine einmonatige Run-In Phase mit Gabe von Verum. Vor Beginn der Run-In Periode werden bei den Probanden Basisparameter (Geschlecht, ethnische Zugehörigkeit, Größe, Gewicht, Taillenumfang, Vorerkrankungen, Familienanamnese (hinsichtlich HNPCC, FAP, Kolerektales Karzinom)), Vormedikation (insbesondere Statine, ASS, NSAID), Nikotinabusus (in Zigaretten pro Tag), Konsum von grünem Tee (in Tassen pro Tag), regelmäßige sportliche Betätigung (in Einheiten von 30 Minuten pro Woche) und Alkoholkonsum erhoben. Zusätzlich erfolgt vor Beginn und am Ende der Run-In Periode bei allen Teilnehmern die Kontrolle laborchemischer Leberparameter (AST, ALT, Bilirubin). Probanden können ihre Studienteilnahme nur fortsetzen, wenn folgende Parameter erfüllt sind:

- Pill-count: Von den 70 Kapseln der Studienmedikation für die Run-In Periode sind weniger als 31 Kapseln übrig
- Die Lebertransaminasen (AST, ALT) und das Bilirubin sind innerhalb des Bereiches des 2,5-fachen Normwertes

5.2 Randomisation

Nach Einschluss in die Studie wird das KKS Halle informiert und es werden das Studienzentrum, der Prüfer, Alter des Probanden, Geschlecht des Probanden und Datum der Koloskopie, Angaben zu Ein- und Ausschlusskriterien sowie Angabe zum Stratum Aspirin-Einnahme an das KKS Halle gefaxt. Der Proband erhält daraufhin eine Probanden-ID-Nummer und beginnt die Run-In Phase.

Nach Durchlaufen der Run-In Phase wird über die weitere Studienteilnahme des Probanden entschieden (siehe 5.1). Bei weiterer Teilnahme erfolgt die Randomisation über das Institut für Biometrie der Universität Ulm. Über die erfolgreiche Randomisation werden das KKSH und die Prüfzentren informiert, das Ergebnis der Randomisation wird an ein unabhängiges

Logistikunternehmen (in entblindeter Form) zur Distribution des Studienpräparates weitergeleitet. Das Logistikunternehmen übernimmt die Lagerung und Versendung des Prüfpräparates an die Prüfzentren. Sie ist zur Verschwiegenheit bezüglich Zuordnung von Studiensubstanz und Probanden-ID verpflichtet.

5.3 Probenentnahme für Biomaterialbanken

5.3.1 Im Rahmen des Zusatzprojektes „Biomarker und Nutrigenetik“

Es werden Vollblutproben zur DNA-Gewinnung sowie Serum- und Plasmaproben zur Bestimmung von biochemischen Verlaufsparametern gelagert und in pseudonymisierter Weise ausgewertet (jeder Proband wird nur anhand der Probanden-ID in der Datenbank geführt). Für die begleitende wissenschaftliche Untersuchung zu Biomarkern für die Adenomentstehung und die Nutrigenetik wird ein Übereignungsvertrag abgeschlossen, durch den die Verwendung des vom Probanden zur Verfügung gestellten Körpermaterials in pseudonymisierter Weise festgelegt wird.

Der Proband wird mündlich und schriftlich aufgeklärt über

- die Menge der Blutentnahme, den Modus der Gewinnung und damit verbundene Risiken
- die Art der Verwendung (biochemische und genetische Untersuchungen)
- Zweck, Dauer und Ort der Verwendung. Eine kommerzielle Verwendung ist ausgeschlossen. Die Proben werden für maximal 20 Jahre aufgehoben und anschließend vernichtet.
- Art und Ausmaß der Pseudonymisierung der Proben. Die Pseudonymisierung erfolgt mit der Randomisierungsnummer, die einen Rückschluss auf die Identität des Spenders nicht ermöglicht.
- absehbare Bedeutung der gewonnenen Erkenntnisse. Es werden explorativ populationsbezogene Biomarker untersucht. Eine direkte Bedeutung für den Betroffenen und /oder seine Familie ist daraus nicht abzuleiten.
- Informationen über Untersuchungsergebnisse werden nur in Form der Veröffentlichung der Ergebnisse vorgenommen, da für den einzelnen Studienteilnehmer eine Information über das Biomarkerprofil keinen Aussagewert hätte.

Der Proband hat die Möglichkeit, an der klinischen Prüfung selbst teilzunehmen, jedoch die Datennutzung und Probenahme für eine Biomaterialbank abzulehnen.

Die Biomaterial- und zugehörige Datenbank wir am Institut für Naturheilkunde und Klinische Pharmakologie der Universität Ulm angelegt.

Die Biomaterialbanken und Datenbanken werden getrennt von der eigentlichen Studienauswertung geführt. Die Proben mit Angabe von Studiencode (MIRACLE), Randomisierungsnummer, Datum der Blutentnahme, Studienvisit-Nummer und Studienzentrum werden direkt an die zuständigen Personen für die Biomaterialbanken gesandt.

Vollblutproben für die Untersuchung genetischer Variabilität können direkt per Post und bei Raumtemperatur an die Biomaterialbank gesandt werden. Die Proben zur Untersuchung von Serum bzw. Plasma im Rahmen des Zusatzprojektes „Biomarker und Nutrigenetik“ werden, wenn entsprechende Infrastruktur dafür vorhanden ist, direkt an den Studienzentren zentrifugiert (Serum/Plasma) und bei -20 ^{0}C tiefgefroren gelagert und gesammelt. Falls keine entsprechende Infrastruktur am Prüfzentrum vorhanden ist, entfallen die entsprechenden Blutabnahmen für Plasma/Serum im Rahmen des Zusatzprojektes.

Die tiefgefrorenen Blutproben werden anschließend halbjährlich auf Trockeneis, welches den Zentren bei Anforderung in geeigneten Versandbehältnissen zur Verfügung gestellt wird, an folgende Adresse verschickt und dort gelagert. Die Lagerung der Proben erfolgt analog den von der Deutschen Krebshilfe für Biobanken aufgestellten Kriterien mit definierten SOPs.

Sektion Klinische Pharmakologie
Institut für Naturheilkunde und Klinische Pharmakologie
Universität Ulm
Helmholtzstr. 20
D-89081 Ulm

5.3.2 Im Rahmen des Zusatzprojektes „Histologische Gewebebank“

Es werden Gewebeproben der entfernten Colonadenome hinsichtlich des Vorliegens von Veränderungen an intrazellulären Signalwegen oder Oberflächenmarkern untersucht (K-ras, B-raf, micro-RNAs). Dafür sollen Gewebeproben von fortgeschrittenen Adenomen (hochgradige Dysplasie, serratierte Adenome) in einer histologischen Gewebebank zentral gesammelt und untersucht werden. Hierfür erfolgt eine separate Aufklärung des Probanden. Zusätzlich wird ein Übereignungsvertrag abgeschlossen, durch den die Verwendung des vom Probanden zur Verfügung gestellten Körpermaterials in pseudonymisierter Weise festgelegt wird.

Der Proband wird mündlich und schriftlich aufgeklärt über

- die Art und Weise des verwendeten Materials
- die Art der Verwendung (immunhistochemische und genetische Untersuchungen)
- Zweck, Dauer und Ort der Verwendung (Eine kommerzielle Verwendung ist ausgeschlossen. Die Proben werden für maximal 20 Jahre aufgehoben und anschließend vernichtet).
- Art und Ausmaß der Pseudonymisierung der Proben (Pseudonymisierung mit der Randomisierungsnummer, die einen Rückschluss auf die Identität des Spenders nicht ermöglicht).
- absehbare Bedeutung der gewonnenen Erkenntnisse
- Informationen über Untersuchungsergebnisse werden nur in Form der Veröffentlichung der Ergebnisse vorgenommen, da für den einzelnen Studienteilnehmer eine Information keinen Aussagewert hätte.

Der Proband hat die Möglichkeit, an der klinischen Prüfung selbst teilzunehmen, jedoch die Gewebenutzung für die Gewebebank abzulehnen.

Die Gewebebank und die zugehörige Datenbank werden an der Universitätsklinik und Poliklinik für Innere Medizin I der Martin-Luther-Universität Halle-Wittenberg angelegt.
Die Gewebebank und Datenbank werden getrennt von der eigentlichen Studienauswertung geführt. Die Gewebeproben (in Paraffin eingebettet) von Probanden, die am Zusatzprojekt „Histologische Gewebebank" teilnehmen, werden bei Nachweis von fortgeschrittenen Adenomen (hochgradige Dysplasie, serratierte Adenome) auf Aufforderung des Studienzentrums von der entsprechenden Pathologie an folgende Adresse versandt (inklusive folgender Angaben: Studiencode (MIRACLE), Randomisierungsnummer, Datum der Koloskopie, Histologischer Befund und Studienzentrum).

Klinisches Studienzentrum
Universitätsklinik und Poliklinik für Innere Medizin I
Universitätsklinikum Halle (Saale)
Ernst-Grube-Straße 40
06120 Halle (Saale)

5.4 Logistik des Versands der Studiensubstanz

Als agierendes Koordinationszentrum der Studie fungiert das Koordinierungszentrum für Klinische Studien Halle (KKSH).

Zur Durchführung der Run-In-Phase erhalten die Studienzentren vorab eine Starterpackung des Prüfpräparates (1 Monats Packung mit Verum). Das KKSH steuert und initiiert die Versendung der Studiensubstanz über die distribuierende Firma an das Studienzentrum. Es werden Pakete mit jeweils mehreren Monatspackungen Studiensubstanz à 70 Kapseln (entsprechend der Intervalle der Studienvisits) pro Proband versandt.

Das KKSH koordiniert außerdem die Versendung der Folgesubstanzen für die Probanden. Dazu werden an die Firma Listen mit den Studienteilnehmernummern versandt, für die jeweils mehrere Monatspakete (à Schachteln mit je 70 Kapseln) versandt werden sollen.

Eine Rückverfolgung auf die Personendaten des Probanden ist nur den behandelnden Ärzten der Prüfzentren sowie der Studienzentrale möglich. Für die Auswertung der Studie werden die Daten bereits unter der Probanden-ID-Nummer verwaltet und die Zuordnungsliste wird getrennt von der Studiendatenbank aufbewahrt.

6 Behandlungsplan

6.1 Beschreibung des Grünteeextraktes:

Hauptwirkstoff: Epigallocatechingallat (ECGC)

Entkoffeinierter Grünteeextrakt in Puderform wird aus jungen, unfermentierten Teeblättern und Knospen der Teepflanze Camellia Sinensis gewonnen. Geeignete Lösemittel sind Alkohol, Methanol, Ethylacetat, Aceton oder Wasser oder Mischungen aus diesen Lösungsmitteln. Koffein muss entfernt werden. Das Verhältnis zwischen Ausgangsmaterial aus der Pflanze und Extraktpuder liegt zwischen 6:1 und 10:1. Grünteeextrakt darf nicht weniger als 60,0% Polyphenole enthalten, sowie nicht weniger als >40,0% (-)-Epigallocatechin-3-O-gallat, EGCG, den wirksamen Hauptbestandteil von Grünteeextrakt. Es darf nicht mehr als 0,5 % Koffein enthalten sein, alles bezogen auf den wasserfreien Extrakt.

Tabelle 1: Charakteristika von Grünteeextrakt Kapseln: Hauptinhaltsstoff >40,0% (-)-Epigallocatechin-3-O-gallat, EGCG

Chemischer Name	(-)-Epigallocatechin-3-O-gallat, EGCG
Summenformel	$C_{22}H_{18}O_{11}$
Handelsname	-
Kapselgröße	Kapseln Größe 0
Menge	150 EGCG
Hersteller	Dr. Loges + Co. GmbH, Winsen
Aktive Inhaltsstoffe	EGCG
Inaktive Inhaltsstoffe	Cellulose, Mg-Stearat
Beschreibung	Pulver

Die Grünteeextrakt-Kapseln sollen aus Hartgelatine (Größe 1) bestehen und enthalten je Kapsel 150mg EGCG sowie Cellulose und Magnesium-Stearat als Fließmittel (keine Lactose wegen möglicher Lactoseintoleranz bei Probanden) als Füllsubstanz. Die Placebokapseln enthalten 100% Füllsubstanz.

Lagerung, Haltbarkeit:

Die Kapseln werden sukzessive vom Hersteller bereitgestellt, so dass über den jeweiligen Studienzeitraum die Haltbarkeitsfrist nicht überschritten wird. Die vorliegenden Daten besagen eine Haltbarkeit unter Raumtemperatur (25°C) von mindestens 24 Monaten. Die Stabilitätstestung wird über den gesamten Verlauf der Studie fortgeführt.

6.2 Kennzeichnung, Beschriftung

Grünteeextrakt sowie Placebo werden durch eine dafür beauftragte Firma gleichartig verkapselt und als MIRACLE Studienpräparat beschriftet (Etikett siehe Anhang). Dadurch ist gewährleistet, dass sowohl Hersteller des Grünteeextraktproduktes, die Studienzentren sowie die Studienzentrale gegenüber der Verpackung verblindet sind. Die Mitarbeiter der Firma, die an der Dispensierung nach Randomisierungsliste beteiligt sind, sind zur Verschwiegenheit verpflichtet. Die Beschriftung auf den Behältnissen und den äußeren Umhüllungen enthält in deutscher Sprache, in gut lesbarer Schrift und auf dauerhafte Weise:

- Bezeichnung: MIRACLE Studienpräparat

- Studien-Registrierungsnummer (www.clinicaltrials.gov): NCT01360320
- Randomisierungsnummer
- LOT-Nummer
- Inhalt und Verzehrempfehlung: 70 Kapseln zur Einnahme: morgens und abends jeweils eine Kapsel mit einer Mahlzeit
- Mindesthaltbarkeitsdatum (MHD)
- Lagerungshinweis: Bei Raumtemperatur lagern
- Kontaktadresse: KKS Halle mit Servicetelefonnummer und Homepage der Studie

6.3 Dokumentation über den Verbleib der Prüfpräparate

Der Prüfarzt des jeweiligen Studienzentrums ist verantwortlich für die sichere und ordnungsgemäße Handhabung und Lagerung der Studiensubstanz am jeweiligen Studienzentrum. Der Prüfarzt stellt sicher, dass die Studiensubstanz nur an Studienteilnehmer verabreicht wird, die gemäß diesem Studienplan in die Studie eingeschlossen worden sind und dass die Studienteilnehmer nur die mit ihrer Randomisierungsnummer gekennzeichnete Substanz erhalten. Die Studiensubstanz wird unter den auf dem Etikett der Prüfpräparate angegebenen Bedingungen gelagert. Unangebrochene, vollständige Packungen (z.B. von ausgeschiedenen Probanden) des Prüfpräparates können von den Prüfzentren zum Logistikunternehmen zur erneuten Ausgabe zurückgesandt werden solange sie unter den entsprechenden Lagerbedingungen in den Prüfzentren aufbewahrt wurden und nie an Probanden ausgehändigt waren.

6.4 Nebenwirkungen, Kontraindikationen

Grünteeextrakt und dessen Hauptinhaltsstoff EGCG ist als gut verträgliches Produkt beschrieben. In Einzelfällen wurde jedoch über das Auftreten von Leberenzymveränderungen im Zusammenhang mit der Einnahme von grünteehaltigen Nahrungsmitteln und Nahrungsergänzungsmitteln berichtet[42]. Von insgesamt 216 Berichten über unerwünschte Ereignisse im zeitlichen Zusammenhang mit der Einnahme von grünteehaltigen Nahrungsmitteln wurde in 7 Fällen ein kausaler Zusammenhang zwischen einer Transaminasenerhöhung (AST, ALT) und der Einnahme von Grünteeextrakt hergestellt. Im Tierversuch wurde eine Aufnahme von EGCG in die Leber beobachtet [43] und in Experimenten an isolierten Rattenleberzellen wurde eine 10-fach höhere Zelltoxizität von EGCG als von anderen Katechinen beobachtet [44].

In der Studie werden die laborchemischen Leberparameter (AST, ALT, Bilirubin) im Blut vor Studieneinschluss, nach einem Monate Einnahme während Run-In, sowie nach 4, 12, 20 und 36 Monaten kontrolliert. Ein Anstieg der Werte über das 2,5-fache des Normwertes führt zum Pausieren der Studienmedikation. Eine Weiterführung der Einnahme der Studiensubstanz darf nur nach Rücksprache mit der Studienleitung erfolgen.

Beim Menschen wurde gezeigt, dass eine Einnahme von Grünteeextrakt auf nüchternen Magen zu fünffach höheren Blutkonzentrationen von EGCG führen kann als eine Einnahme mit dem Essen[36,37]. Deshalb wird in dieser Studie darauf hingewiesen, die Studiensubstanz mit einer Mahlzeit einzunehmen. Außerdem werden die Probanden, die an dieser Studie teilnehmen, darüber aufgeklärt, bei Zeichen einer Lebererkrankung, Bauchschmerzen, Gelbfärbung der Skleren oder Dunkelfärbung des Urins, sofort Kontakt mit dem jeweiligen Arzt des Studienzentrums aufzunehmen.

Es gibt keine bekannten Kontraindikationen für Grüntee bzw. Grünteeextrakt. In einer Studie zur Kinetik von EGCG von Chow wurde über 4 Wochen eine Dosis von 800 mg EGCG an gesunde Probanden verabreicht. Die in diesem Zeitraum berichteten Beschwerden (Kopfschmerz, Magenbeschwerden, Bauchschmerz und Übelkeit) waren leicht und nicht unterschiedlich zu der Gruppe, die Placebo erhielt[37]. In anderen Studien zu Grünteeextrakt an Probanden mit soliden Tumoren wurden Beschwerden, wie Blähungen, Flatulenz, Dyspepsie, Übelkeit, sowie Unruhe, Schwindel und Schlaflosigkeit beschrieben, was jedoch wahrscheinlich dem 7%igen Koffeingehalt der in dieser Studie verwendeten Extrakte zuzuschreiben ist[45]. In einer Studie zur Sekundärprävention von Prostatakarzinomen wurden bei Einnahme von 600 mg Grünteekatechinen / Tag gelegentlich Durchfälle berichtet[24].

6.5 Wechselwirkung mit anderen Substanzen

Wechselwirkungen sind denkbar, aber nicht bekannt. Zu erwarten wären beispielsweise ein verminderter Auswärtstransport durch Wechselwirkung mit Inhibitoren von Multidrug Resistance Protein (MRP) - Transportern, wie z.B. Indometacin, was zu einer verstärkten Wirkung führen könnte. Auch ist eine klinische Wechselwirkung zwischen Grünteeextrakt und Medikamenten, welche über p-Glykoprotein transportiert werden, denkbar. Dies ist aber klinisch bislang nicht untersucht.

6.6 Pharmakokinetik

Grünteekatechine werden als monomere Flavan-3-ole über den Dünndarm resorbiert. Katechine erreichen Maximalkonzentrationen im Plasma nach ca. 1-4 Stunden und sind bei einer Plasmahalbwertszeit von ca. 3 Stunden nach 24 Stunden wieder ausgeschieden[46,47].

Es besteht ein aktiver Efflux-Transport über MRP-1 und -2 über das Darmepithel ins Lumen und aus den Leberzellen hinaus. P-Glykoprotein wird durch Grünteekatechine (EGCG und weitere) gehemmt [48]. Anschließend werden Katechine in der Leber vorwiegend über Phase II Enzyme metabolisiert (glucuronidiert, methyliert und sulfatiert). Die Elimination erfolgt innerhalb der ersten 6 Stunden nach Einnahme biliär (EGCG) und renal (weitere Katechine).

Im Rahmen des Zusatzprojektes „Nutrikinetik von Epigallokatechingallat (EGCG) in oraler Formulierung: Populationsvariabilität und Nutrigenomik“ dieser Studie soll an einer unabhängigen Kohorte von n=100 gesunden Probanden eine Untersuchung zur multiple dose Kinetik von EGCG 2x150 mg/d durchführt werden. Dafür werden gesondert ein Studienplan, Einwilligungserklärungen und Probandeninformation verfasst.

6.7 Pharmakodynamik

EGCG und andere Katechine binden an den 67 kDa Laminin-Rezeptor und lösen eine Reihe von intrazellulären Signalmechanismen aus, die das Krebswachstum hemmen: Induktion von Apoptose in Krebszellen über Hemmung des Proteasoms, Hemmung der Aktivierung von Tyrosinkinasen (EGFR, HER2 und -3), sowie Angiogeneseinhibition, Hemmung der Aktivität der Topoisomerase I und Reduktion der COX-2-Expression in Tumorzellmodellen. Letzteres wird auch für die chemoprotektive Wirkung von Aspirin oder selektiven COX-2 Hemmstoffen verantwortlich gemacht.

6.8 Notfallmaßnahmen

Es sind bisher unter Einnahme von Grünteeextrakt keine Notfallsituationen wie z.B. eine Überempfindlichkeitsreaktion beschrieben worden. Bei Intoxikationen in großen Mengen müssen die üblichen Notfallmaßnahmen (Ruf eines Notarztes, Primärversorgung am Ort, Transport in eine Klinik etc.) getroffen werden. Im Falle eines Notfalls muss die Studienleitung umgehend (zumindest innerhalb von 24 h) über den Notfall per Faxvordruck informiert werden.

Bei Auftreten von klinischen Situationen, die Kenntnis der Behandlung (mit nachfolgendem Studienausschluss) erfordern, z.B. schwere Interaktionen, kann eine Entblindung erfolgen. Dafür wird eine Entblindungsliste (beim Randomisierungsverfahren automatisch erstellt durch das Institut für Biometrie in Ulm) bei der Studienleitung gelagert.

6.9 Behandlungsschema

Einnahme je einer Kapsel Studiensubstanz (Grünteeextrakt oder Placebo) zusammen mit einer Mahlzeit über 3 Jahre (entsprechend maximal 300 mg EGCG täglich im Verumarm). Falls die morgendliche Einnahme vergessen wurde, kann sie auch später am Tag, aber stets mit einer Mahlzeit, erfolgen.

6.10 Begleittherapie

Die vollständige Medikation der Probanden wird während des Beobachtungszeitraumes von 3 Jahren bei den Studienvisits alle 4 Monate erfasst. Probanden, die wegen anderer Erkrankungen regelmäßig nichtsteroidale Antirheumatika, COX-2 Inhibitoren oder mehr als 100 mg/d Acetylsalicylsäure über einen Zeitraum, der länger als 3 Monate pro Jahr beträgt, einnehmen, werden von der weiteren Teilnahme an der Studie ausgeschlossen (Dropout).

7 Klinische Untersuchungen

7.1 Eingangs-/Einschlussuntersuchung

Potentielle Studienteilnehmer sind Probanden, die in den Prüfzentren eine Koloskopie mit Entfernung von Adenomen, z.B. im Rahmen der Darmkrebsvorsorge erhalten haben. Ziel ist es, in den Studienzentren die Studie bei sämtlichen Patienten, die im Rahmen der Darmkrebsvorsorge eine Koloskopie erhalten, bekannt zu machen und sämtliche Patienten, bei denen eine Adenomentfernung vorgenommen wurde, über die Studie zu informieren und bezüglich Teilnahme aufzuklären. Nach Prüfung der Ein- und Ausschlusskriterien werden die für die Studienauswertung notwendigen Basisdaten erhoben. Der zeitliche Abstand zwischen Polypektomie und Beginn der Studiensubstanzeinnahme darf maximal 6 Monate betragen.

Die Daten, die bei der Eingangsuntersuchung erhoben werden und für die Studienauswertung dokumentiert werden, sind:

- Gewicht, Größe, Taillenumfang in cm, Hüftumfang in cm
- Ethnische Zugehörigkeit (Nachfrage Herkunft und Nationalität Eltern, Großeltern)
- Raucherstatus
- Alkoholkonsum (in Flaschen Bier pro Tag, Gläsern Wein pro Tag, 2cL Gläsern Spirituosen pro Tag)
- Ernährungsgewohnheiten/Grünteekonsum als Tee oder in Form von Grünteeextrakt
- Bewegungsgewohnheiten/Sport pro Woche (1 Einheit = 30 min)
- Medikation (vollständige zum Zeitpunkt des Studieneinschlusses eingenommene Medikamente unter Angabe der Tagesdosis und der Dauer der Einnahme), Vitaminpräparate
- Begleiterkrankungen
- Familienanamnese hinsichtlich Darmkrebserkrankungen (hinsichtlich HNPCC, FAP, Kolorektales Karzinom)
- Bestimmung von laborchemischen Leberparametern (AST, ALT, Bilirubin)
- Ausfüllen des Fragebogens „Alternative Heilmethoden“

Die Erhebung klinischer Risikofaktoren für die Entstehung von Kolonkarzinomen ist notwendig, um mögliche Einflussfaktoren, die in den Vergleichsgruppen eventuell nicht gleich verteilt vorliegen könnten, zu erkennen.

7.2 Verlaufsuntersuchungen

Es erfolgen alle 4 Monate Verlaufsuntersuchungen zur Verträglichkeitsbeurteilung, zur Erhebung von etwaigen unerwünschten Ereignissen, zur Dokumentation etwaiger Änderungen der Komedikation (z.B. ASS, NSAID) und zur Überprüfung der Einnahmefrequenz im jeweiligen Studienzentrum. Die Probanden erhalten möglichst bereits am Tag des Zentrumsbesuches einen Termin für den nächsten Zentrumsbesuch in 4 Monaten. Nach 4, 12, 20 und 36 Monaten wird jeweils eine Kontrolle laborchemischer Leberparameter (AST, ALT, Bilirubin) sowie nach 4, 20 und 36 Monaten eine Blutentnahme für spätere Biomarkeruntersuchungen durchgeführt (Einwilligung in das Zusatzprojekt „Biomarker und Nutrigenetik vorausgesetzt). Dazu ist es notwendig, dass der Proband beim behandelnden Prüfzentrum vorstellig wird. Es wird jeweils eine Befragung mit Hilfe des Probandentagebuchs zu Beschwerden und zur Einnahme des Studienpräparates durchgeführt sowie Änderungen des Komedikation (speziell hinsichtlich NSAID, ASS) erfragt. Anschließend wird dem Probanden die Studiensubstanz für die nächsten 4 Monate ausgehändigt. Bei festgestellter

Non-Compliance, aber auch bei nicht vom Probanden zu verantwortenden Unterbrechungen der Einnahme (z.B. bei medizinischen Eingriffen) entscheidet die Studienleitung über die weitere Studienteilnahme.

7.3 Abschlussuntersuchung

Die Erhebung der primären und sekundären Zielparameter der Studie erfolgt bei der Kontrollkoloskopie nach 3 Jahren. Falls bei Probanden bereits nach einem Jahr oder vor Ablauf der 3 Jahre eine Kontrollkoloskopie durchgeführt wird, werden die Daten bei diesen Untersuchungen ebenfalls dokumentiert. Falls in diesem Fall nach 3 Jahren eine weitere Koloskopie erfolgt, sollen die Teilnehmer bis zu dieser Untersuchung die Studienmedikation weiter einnehmen. Bei der Kontrollkoloskopie werden makroskopische und histopathologische Befunde der endoskopischen Untersuchungen dokumentiert und wiederum Daten zum Gesundheitszustand des Probanden erhoben, zur Medikation, zur Einnahme der Prüfkapseln in den letzten Monaten und zu etwaigen Beschwerden. Danach endet die Einnahme von Grünteeextrakt.

Unabhängig von dieser Studie sind Follow-ups in dieser Kohorte nach weiteren drei Jahren geplant. Eine längere Nachverfolgung der Kohorte auch nach Abschluss der Einnahmephase, - bis zu einer zweiten Kontroll-Koloskopie nach 6 Jahren - ist sinnvoll, da die Entwicklung von Karzinomen teilweise längere Zeit dauert und in klinischen Studien gezeigt wurde, dass die protektive Wirkung von chemopräventiven Substanzen wie z.B. Acetylsalicylsäure teilweise erst nach ca. 10 Jahren nachweisbar ist [32]. Bezüglich der Follow-ups wird zu gegebener Zeit die separate Einwilligung der Probanden eingeholt.

7.4 Studienbedingte Blutabnahmen

Blutentnahmen werden zu Beginn der Studie (Einschlussuntersuchung), sowie bei den Verlaufsuntersuchungen und zum Zeitpunkt der Abschlussuntersuchung durchgeführt.

Es werden folgende Blutentnahmen gemacht:

1) Bei Einschluss, sowie nach Monat 1, 4, 12, 20 und 36 jeweils Kontrolle der Leberwerte AST, ALT und Bilirubin.
2) Nach Monat 1 (wenn eine Randomisation erfolgt), sowie nach Monat 4, 20 und 36: Serum (7ml) und Plasma (7ml) zur Lagerung in der Biobank zur Bestimmung von Biomarkern.
3) Einmalig nach Monat 1 (wenn eine Randomisation erfolgt): 7 ml EDTA-Vollblut für genetische Untersuchungen: Geplant ist, zumindest an einem Teil der Probandenproben

genomweite Untersuchungen zu machen, um Suszeptibilitätsloci für ein schnelles Wiederauftreten von Adenomen zu untersuchen. Daneben sollen Kandidatengenanalysen - etwa zu genetischen Polymorphismen in der Pharmakokinetik von Epigallokatechingallat - durchgeführt werden.

Insgesamt beträgt die Menge des studienbedingt abgenommenen Blutes 63 ml für die Biobank und ca. 18 ml für die Bestimmung der laborchemischen Leberparameter (AST, ALT, Bilirubin).

7.5 Referenzinstitutionen

Die histologischen Untersuchungen bei der Kontrollkoloskopie werden von den lokalen Pathologien der jeweiligen Prüfzentren durchgeführt. Die Bestimmung der laborchemischen Leberparameter (AST, ALT, Bilirubin) erfolgt durch das jeweilige Studienzentrum.

Die Proben des Zusatzprojektes „Biomarker und Nutrigenetik“ werden am Institut für Naturheilkunde und Klinische Pharmakologie der Universität Ulm gesammelt.

Die Gewebeproben (in Paraffin eingebettet) von Probanden, die am Zusatzprojekt „Histologische Gewebebank“ teilnehmen werden an das Klinische Studienzentrum der Universitätsklinik und Poliklinik für Innere Medizin I der Universitätsklinik Halle (Saale) verschickt und dort zentral gesammelt.

7.6 Späterer Ausschluss von Probanden

Patienten können nach der Run-In Periode, die Teilnahme an der Studie nicht fortsetzen, wenn:

- Der Pill-count ergibt, dass von den 70 Kapseln pro Packung des Prüfpräparates für die Run-In Periode mehr als 31 Kapseln übrig sind
- Die laborchemischen Leberparameter (AST, ALT, Bilirubin) über das 2,5-fache des Normwertes erhöht sind.

die Teilnahme wird beendet, wenn:

- nicht zu tolerierende Beschwerden auftreten

- die Compliance des Studienteilnehmers mehrfach unzuverlässig ist (z.B. Medikamenteneinnahme im 4-Monatszeitraum unter 65%)
- wenn aus anderen, nicht vom Probanden zu verantwortenden Gründen die regelmäßige Einnahme der Studiensubstanz nicht gewährleistet ist
- nachträglich festgestellt wird, dass die Einschlusskriterien nicht erfüllt sind
- eine Situation eintritt, die aus medizinischen Gründen die kontinuierliche Einnahme von nichtsteroidalen Antirheumatika, COX-2 Hemmern oder hochdosierter Acetysalicylsäure (> 100 mg) > 3 Monate pro Jahr erforderlich macht
- der Proband es ausdrücklich wünscht
- bei Erhöhung der laborchemischen Leberparameter (AST, ALT, Bilirubin) um mehr als das 2,5-fache des Normwertes

Im Falle eines späteren Ausschlusses werden die entsprechenden Gründe mit Angabe des Datums und der letzten Medikation im Studienbogen notiert. Die Daten werden wie die restlichen Daten in pseudonymisierter Form gespeichert. Falls bei diesen Probanden eine Follow Up-Koloskopie durchgeführt wird, soll das Ergebnis dieser Untersuchung möglichst trotzdem im Rahmen der Studie dokumentiert werden (ITT-Kollektiv). Hiervon auszunehmen ist der Ausschluss aus der Studie bei Entzug der Probandeneinwilligung und damit einhergehendem Erlöschen der Einwilligung zur Datenweitergabe. Es werden etwa 15 % Dropouts nach Abschluss der Run-In-Phase und 5 % Dropouts nach Randomisation erwartet.

8 Methoden zur Erfassung der Wirksamkeit und der chemoprotektiven Effektivität

8.1 Zeitplan

Jeder Proband wird nach Polypektomie über 3 Jahren beobachtet und erhält über diese Zeit verblindet täglich zwei Kapseln mit Grünteeextrakt oder Placebo zur Einnahme.

8.2 Wirksamkeit

Die prophylaktische Wirkung wird anhand der Inzidenz metachroner Adenome oder serratierter Läsionen bei der Kontrollkoloskopie, ihrer Größe, Lokalisation und histologischen Klassifikation

(tubuläre, villöse, tubulovillöse, serratierte Adenome) sowie der Inzidenz schwerer Dysplasien und Karzinome beurteilt.

Es wird auf die histologischen Befunde der Kontrollkoloskopien zurückgegriffen. Eine Beurteilung durch einen Referenzpathologen ist nicht vorgesehen.

8.3 Individuelle und allgemeine Abbruchkriterien

Einer oder die Kombination mehrerer der folgenden Umstände (siehe auch 7.6) können zu einem Abbruch der Studie bei einem einzelnen Probanden führen:

- Auftreten eines Abbruchkriteriums (siehe 7.6)
- Protokollverletzung durch den Studienteilnehmer (z. B. Non-Compliance, siehe 7.6)
- Widerrufen der Einwilligungserklärung

Der verantwortliche Arzt ist befugt, die Prüfung zu jedem beliebigen Zeitpunkt abzubrechen, wenn dies im Interesse des Probanden angebracht erscheint; diese Gründe gelten als Abbruchkriterien.

9 Methoden zur Erfassung der Sicherheit – unerwünschte Ereignisse

9.1 Definitionen unerwünschter Ereignisse

Unerwünschtes Ereignis

Ein unerwünschtes Ereignis (adverse event = AE) ist jedes nachteilige Vorkommnis, das einer betroffenen Person widerfährt, der ein Prüfpräparat verabreicht wurde und das nicht notwendigerweise in ursächlichem Zusammenhang mit dieser Behandlung steht.

Schwerwiegendes unerwünschtes Ereignis

Ein schwerwiegendes unerwünschtes Ereignis (serious adverse event = SAE)

- ist lebensbedrohlich oder tödlich
- führt zu einer signifikanten oder andauernden Behinderung
- führt zur Krankenhauseinweisung oder Verlängerung eines Krankenhausaufent-haltes
- führt zu einer schwerwiegenden Behinderung oder Invalidität
- führt zu einer kongenitalen Anomalie oder einem Geburtsfehler

- ist darüber hinaus jedes medizinische bedeutsame Ereignis, das den Studienteilnehmer vorübergehend schädigen kann (z. B. Bronchospasmus, Krampfanfall), ohne eines der obengenannten Kriterien zu bedienen.

Nicht schwerwiegende unerwünschte Ereignisse sind alle Ereignisse, auf die keine der o. g. Kategorien zutrifft.

9.2 Einteilung unerwünschter Ereignisse

9.2.1 Schweregrade unerwünschter Ereignisse

Grad 1 leichtes AE:	Ereignis/Symptom beeinträchtigt nicht alltägliche Aktivitäten
Grad 2 mäßiges AE:	Ereignis/Symptom beeinträchtigt alltägliche Aktivitäten
Grad 3. Schweres AE:	Ereignis/Symptom verhindert alltägliche Aktivitäten

9.2.2 Kausalzusammenhang unerwünschter Ereignisse

Sicher: Ein klinisches Ereignis, einschließlich eines abnormen Laborbefundes, das in plausiblem zeitlichen Bezug zum Zeitpunkt der Medikationseinnahme steht und das nicht durch eine Begleiterkrankung oder andere Arzneimittel erklärbar ist. Die Antwort auf das Absetzen des Arzneimittels muss klinisch plausibel sein. Das Ereignis muss pharmakologisch oder phänomenologisch eindeutig sein, wobei – falls erforderlich – eine wegweisende Rechallenge durchgeführt werden sollte.

Wahrscheinlich: Ein klinisches Ereignis, einschließlich eines abnormen Laborbefundes, das in plausiblem zeitlichen Bezug zum Zeitpunkt der Medikationseinnahme steht und dessen Zusammenhang mit einer Begleiterkrankung oder anderen Arzneimitteln unwahrscheinlich ist. Die Antwort auf das Absetzen des Arzneimittels sollte klinisch plausibel sein. Angaben zur Reexposition sind nicht erforderlich, um diese Definition zu erfüllen.

Unwahrscheinlich: Ein klinisches Ereignis, einschließlich eines abnormen Laborbefundes, mit möglichem zeitlichem Bezug zur Medikationseinnahme, das jedoch eher durch eine Begleiterkrankung oder andere Arzneimittel erklärt werden könnte. Angaben zum Absetzen dürfen fehlen oder unklar sein.

Kein Zusammenhang: Der zeitliche Bezug zwischen dem klinischen Ereignis (einschließlich eines abnormen Laborbefundes) und der Medikamenteneinnahme macht einen

Kausalzusammenhang unwahrscheinlich. Das Ereignis ist durch andere Erkrankungen, Umstände oder Arzneimittel erklärbar.

9.2.3 Gegenmaßnahmen

Gegenmaßnahmen werden wie folgt klassifiziert:

- keine (d. h. keine Änderung der Studienmedikation)
- Absetzen der Studienmedikation
- medikamentöse Behandlung
- Hospitalisierung.

9.2.4 Ausgang unerwünschter Ereignisse

Der Ausgang ("outcome") unerwünschter Ereignisse wird wie folgt klassifiziert:

- Wiederhergestellt
- anhaltend
- nicht wiederhergestellt
- wiederhergestellt mit Folgesymptomen
- tödlich
- unbekannt.

9.3 Erfassung und Dokumentation

AEs können spontan vom Probanden berichtet werden, durch allgemeine Fragen des Prüfarztes erfragt werden oder durch eine körperliche Untersuchung bzw. Laborbefunde zu jeder Zeit während des gesamten klinischen Studienverlaufs entdeckt werden. Fragen an den Probanden müssen offen, d. h. nicht-suggestiv, gestellt werden.

Im CRF werden folgende Merkmale von AEs dokumentiert:

Art des AE (Diagnose, Symptom), Dauer (Beginn, Ende), SAE ja/nein, Schweregrad, Kausalzusammenhang mit Prüfmedikation, Gegenmaßnahmen, Fortführung der Studienmedikation, Ausgang.

Pathologische Laborbefunde, die als AEs klassifiziert werden, sind in Tabelle 2 zusammengefasst.

Fortbestehende SAEs müssen vom zuständigen Prüfarzt solange nachbeobachtet werden, bis sie abgeklungen sind bzw. bis sich der Zustand des Probanden stabilisiert hat.

Tabelle 2: AE-Kriterien für pathologische Laborbefunde

Parameter	Kriterien für ein AE
AST (GOT)	>2,5 x obere Normgrenze
ALT (GPT)	>2,5 x obere Normgrenze
Bilirubin (gesamt)	>2,5 x obere Normgrenze

9.4 Berichterstattung

9.4.1 Berichterstattung durch den Prüfarzt

Der Prüfer hat jedes schwerwiegende unerwünschte Ereignis (SAE) unverzüglich (innerhalb von 24h nach Bekanntwerden) an folgende Adresse per Fax zu melden:

Prof. Dr. Thomas Seufferlein
Universitätsklinik und Poliklinik für Innere Medizin I
Martin-Luther-Universität Halle-Wittenberg
Ernst-Grube-Straße 40
06120 Halle (Saale)
Tel: 0345 / 5 57 26 63
Fax: 03 45 / 5 57 22 53
E-Mail: thomas.seufferlein@uk-halle.de

Für jedes dieser Ereignisse ist ein Berichtsbogen (Vordruck) auszufüllen und umgehend an die angegebene Adresse (siehe oben Prof. Seufferlein) weiterzuleiten. Wenn die erforderlichen Informationen zu diesem Zeitpunkt nicht komplett verfügbar sind, müssen Folgeberichte abgefasst werden. Bei Todesfällen sollte nach Möglichkeit eine Kopie des Autopsieberichts beigelegt werden.
Ausnahmeregelungen betreffen schwerwiegende Ereignisse, die nach der Registrierung, jedoch vor Behandlungsbeginn eintreten. Sowie eine geplante Hospitalisierung aufgrund von therapeutischen Maßnahmen, die nicht im Zusammenhang mit der Studie stehen.

Die an der Studie beteiligten Prüfer/Prüfzentren werden vom Studienleiter/Leiter der klinischen Prüfung über alle gemeldeten Ereignisse (SAEs) jährlich informiert. Ereignisse, für die ein kausaler Zusammenhang mit dem Studienpräparat nicht auszuschließen ist, werden den Prüfzentren unverzüglich mitgeteilt.
Ein vollständiger Bericht (ggf. einschließlich Kopien der Krankenakten mit geschwärzten Patientennamen und mit der Probandennummer versehen) sowie eine Zusammenfassung des Ausgangs und eine Stellungnahme des Prüfarztes zu einem möglichen Kausalzusammenhang mit einem Prüfpräparat sollte innerhalb von 14 Tagen an die Studienleitung übermittelt werden.

10 Dauer der Studie / Studienabbruch

10.1 Reguläres Studienende

Die Rekrutierung wird beendet, wenn 2534 Probanden nach Run-In in die Studie eingeschlossen und randomisiert wurden. Die Auswertung beginnt erst dann, wenn alle zum Zeitpunkt des Rekrutierungsstopps eingeschlossenen Probanden die Studie regulär oder als Drop-Out beendet haben. Unter Annahme einer Fehlerrate von 5% bei den 3-jährigen Follow-Up Koloskopien ist insgesamt eine Zahl von 1203 Probanden pro Studienarm zu erwarten (Intention to treat).

10.2 Vorzeitiges Studienende / Studienabbruch

Die Studie kann vorzeitig abgebrochen werden, wenn während der Laufzeit der Studie neue wissenschaftliche Erkenntnisse eine eindeutige Unterlegenheit der Prophylaxe zeigen, wenn aufgrund einer in der Studienpopulation auftretenden Häufung von Nebenwirkungen, Adenomen oder Kolonkarzinomen eine vorzeitige Entblindung und Auswertung erfolgt, sowie wenn die Rekrutierungsrate nicht adäquat sein sollte. Die Entscheidung über den Abbruch der Studie wird von der Studienleitung getroffen.

11 Biometrische Planung und Auswertung

11.1 Zielgrößen

11.1.1 Primäres Studienziel

Untersuchung des Effektes von Grünteeextrakt im Vergleich zu Placebo auf die Inzidenz metachroner kolorektaler Adenome (tubulovillöse, tubuläre, villöse Adenome, serratierte Läsionen) bei einer 3-Jahres Follow-up Koloskopie nach initialer kompletter Adenomentfernung.

11.1.2 Sekundäre Ziele

- Zahl, Lokalisation, Größe und histologischer Subtyp der kolorektalen Adenome oder serratierten Läsionen (tubulär, villös, tubulovillös, serratiert, hyperplastisch, Grad der Dysplasie, invasives Wachstum) sowie die Zeitspanne bis zum Wiederauftreten von Adenomen bei Koloskopiefrequenz < 3 Jahren.
- Frequenz schwerer Dysplasien und Karzinome in beiden Gruppen.
- Explorative Auswertungen zur genetischen, biochemischen und metabolomischen Suszeptibilität für Adenomentstehung (Zusatzprojekt „Biomarker und Nutrigenetik“).
- Immunhistochemische und genetische Untersuchungen der histologischen Gewebeproben von Probanden mit fortgeschrittenen Polypen (hochgradige Dysplasie oder serratierte Adenome) hinsichtlich häufig in der Adenom/Karzinomentstehung vorkommender Gewebemerkmale (K-ras-Mutation, B-raf-Mutation, spezifische micro-RNAs) (Zusatzprojekt „Histologische Gewebebank“)
- Untersuchung der Konzentration und Kinetik von EGCG und dessen Metabolite in Plasma bei einer Stichprobe (n=100 gesunde Probanden, Zusatzprojekt „Nutrikinetik“).
- Einfluss der Meinung zu „alternativen Heilmethoden“ der Probanden (Fragebogen) auf die primären und sekundären Zielgrößen und die Compliance

11.2 Definition von Auswertungskollektiven

11.2.1 Intention-to-treat Population

Die Intention-to-treat Population wird für die Auswertung der Hauptfragestellung (Koloskopie nach 3 Jahren) bzgl. der Wirksamkeit ausgewertet. Es werden die vorliegenden Informationen aller

randomisierten Teilnehmer berücksichtigt. Dabei wird analog zu Baron (2006) eine modifizierte ITT-Analyse herangezogen, nach der alle Probanden, von denen nach 3 Jahren eine Koloskopie vorliegt, die ITT-Population bilden. Es wird davon ausgegangen, dass bei 5% der Probanden dies nicht der Fall ist. Ausgegangen wird weiterhin davon, dass von diesen Probanden 20% Drop-outs bezüglich der Einhaltung des Studienprotokolls darstellen. Die fehlenden Einflussparameter werden mit Methoden der Ersetzung fehlender Werte (hier multiple Imputation mit MCMC-Methodik, 10 Ersetzungen) abgeschätzt.

11.2.2 Per-protocol-Population

Die per-Protocol-Population umfasst die Daten der Probanden mit komplettem Datensatz, die protokollgerecht 3 Jahre behandelt und untersucht werden konnten. Sicherheitsparameter werden bei allen Probanden, die zumindest eine Kapsel Prüfmedikation eingenommen haben, vergleichend zwischen Placebo und Grünteeextrakt ausgewertet.

11.3 Planung des Studienumfangs

Die Fallzahlschätzung wird basierend auf dem primären Zielparameter durchgeführt. Es ist bekannt, dass etwa 45 % der Probanden mit Polypen in der Vorgeschichte bei der Kontroll-Untersuchung nach 3 Jahren wiederum Adenome aufweisen[30]. In einer Studie zur Chemoprävention mit Aspirin betrug die Häufigkeit des Wiederauftretens eines oder mehrerer Adenome 47%. In der Gruppe Probanden, die Aspirin erhielten (bestehend aus Low-dose und High dose), traten nur bei 41 % ein erneutes Adenom auf[30]. Dies entsprach einer relativen Risiko-Reduktion von 12.7 %. Wir würden einen Effekt in ähnlicher Größenordnung für die Wirkung des Grünteeextraktes zugrunde legen.

Unter Annahme, dass in der gesamten ITT-Population aufgrund von mäßigem Grüntee-Genuss in beiden Gruppen die Häufigkeit des Wiederauftretens insgesamt etwas niedriger liegen wird, wird die Fallzahlschätzung auf der Basis einer angenommenen Inzidenz von 45 % in der Placebogruppe und 40 % in der Grünteegruppe, resultierend in einer relativen Risikoreduktion von 11 %, durchgeführt. Dies entspricht einer Odds Ratio von 0.815.

Die Fallzahlberechnung wird unter Einbeziehung des Stratums Low-dose Aspirin-Einnahme basierend auf den Daten aus Baron et al. mit dem stratifizierten Mantel-Haenszel-Test durchgeführt.[30] Es wird angenommen, dass 20 % der Studienteilnehmer Low-Dose 100 mg Aspirin dauerhaft einnehmen werden. Für die Berechnung der Häufigkeiten in den Schichten 20 % Aspirin

100 mg und 80% ohne Aspirin ≤100 mg werden die Häufigkeiten des Wiederauftretens in der Grüntee / Low-Dose Aspirin-Gruppe aus Baron et al. (38,3%) und in der Placebogruppe ohne Aspirineinnahme (47,1%) verwendet.[30]

Mit alpha = 5 %, Power = 80 % sowie einseitigem stratifiziertem Mantel-Haenszel-Test ergibt sich hieraus im Fallzahlprogramm NQUERY 7.0 eine notwendige Fallzahl von n=1203 pro Gruppe (ITT-Kollektiv). Bei 5% fehlenden Koloskopien nach 3 Jahren benötigt man dann jeweils 1267 randomisierte Probanden pro Gruppe. Das wären insgesamt 2534 Probanden. Im Falle von weiteren 20% Drop-out verbleiben dann jeweils 1014 Probanden in jeder Gruppe für die Per Protocol-Analyse.

Für explorative multivariate Auswertungen zur Kontrolle von Confoundern ist diese Fallzahl ausreichend, um eine Anzahl von etwa 30-40 Variablen inklusive Dummy-Codierung bei einer Vorgabe von etwa 20 Events pro Variable (EPV) in die Modellierung einschließen zu können.

11.4 Planung der statistischen Auswertung der Hauptzielgröße

Konfirmatorisch: Mit der Studie wird eine statistische Absicherung der Hypothese „die regelmäßige Einnahme von Grünteeextrakt (mindestens 300 mg EGCG täglich) über drei Jahre führt zu einer Verminderung der Inzidenz von metachronen Kolonadenomen" angestrebt. Diese Hypothese wird konfirmatorisch mit dem Chi-Quadrat-Test einseitig auf dem 5%-Signifikanzniveau auf Basis der ITT-Population geprüft.

Zusätzliche (explorative) Analysen: Der Einfluss weiterer Variablen, speziell zur Kontrolle der beiden Strata-Variablen aus der Randomisierung (Studienzentrum und niedrigdosiertes ASS ≤100mg/d) wird in Bezug auf das Auftreten von Adenomen anschließend in diesem ITT-Kollektiv explorativ als sekundäre Analyse mit dem logistischen Regressionsmodell und in Bezug auf die Zeit bis zum Auftreten von Adenomen mit dem Cox Proportional Hazard Modell kontrolliert. Mittels dieser multivariater Analysen werden die relativen Risiken und die 95% Konfidenzintervalle errechnet.

11.5 Explorative Auswertungen der sekundären Zielgrößen

Explorativ soll ausgewertet werden, inwieweit andere Einflussfaktoren das Risiko für die Inzidenz von metachronen Adenomen beeinflussen.

Es ist geplant, für die Subgruppen mit der höchsten Adenominzidenz und einer Kontrollgruppe aus Probanden, die kein Adenom entwickelten, genomweite explorative Untersuchungen

durchzuführen. Für den Fall, dass genetische Polymorphismen von den genomweiten Untersuchungsmethoden nicht detektiert werden (diese detektieren ausschließlich SNPs), werden diese in einem Kandidatengen-Ansatz untersucht. Dies wird insbesondere für Polymorphismen, die in früheren Studien mit der Adenomentstehung assoziiert waren, durchgeführt werden, z.B. für Polymorphismen in CYP2E1, im Dopaminrezeptor D2, sowie in Glutathiontransferasegenen.

Es sollen außerdem explorativ Biomarker im Plasma und Serum für das Wiederauftreten von Adenomen bei Probanden nach Polypenentfernung untersucht werden.

11.6 Sicherheitsanalyse

Die Sicherheitsanalyse erfolgt getrennt für jeden Therapiearm. Es wird erfasst und ausgewertet:

- Anzahl der unerwünschten Ereignisse
- Laborwertveränderungen

12 Datenmanagement

12.1 Datenerfassung, -auswertung und -weitergabe

Alle im Rahmen der Studie anfallenden Daten unterliegen dem Datenschutz. Personengebundene Daten werden mit Ausnahme von Alter und Geschlecht weder vom Leiter der Studie noch von den beteiligten Prüfern weitergegeben. Die Weitergabe von klinischen Daten und die Datenspeicherung zur Auswertung erfolgt nur unter Pseudonym (Probanden-ID). Nach Abschluss der Studie werden die Studienunterlagen gemäß den Bestimmungen des Datenschutzes archiviert. Personen, denen ein Zugriff auf die Daten ermöglicht wird, unterliegen der Schweigepflicht. Eine Weitergabe der Daten ist ihnen untersagt.

Alle Daten werden in Papier-CRFs erfasst. Datenmanagement, Monitoring und Querymanagement werden vom KKS Halle durchgeführt. Die Dateneingabe von den Papier-CRFs in eine validierte, GCP-konforme Datenbank erfolgt mit den üblichen Verfahren zur Qualitätskontrolle bei klinischen Studien durch das KKS Halle (Konsistenzchecks, „second look“). Pflege der Datenbank, Datensäuberung und -export, sowie die Auflistung unerwünschter Ereignisse werden ebenfalls durch das KKS Halle durchgeführt, das über entsprechende SOPs und Working Instructions verfügt.

Die Datenauswertung wird im Institut für Biometrie der Universität Ulm durchgeführt.

13 Qualitätssicherung

13.1 Advisory Board

Es wird ein beratendes Advisory Board eingerichtet, welches in regelmäßigen Abständen über den Studienverlauf unterrichtet und konsultiert wird.

13.2 Standardisierung, Validierung und Monitoring

Das Monitoring wird durch das Koordinierungszentrum für Klinische Studien der Martin-Luther-Universität Halle-Wittenberg (KKSH) übernommen und im Rahmen von Vor-Ort-Monitoringbesuchen vorgenommen. Die Besuchsfrequenz während der laufenden Studie hängt von der Rekrutierung am Prüfzentrum ab.

Es werden u. a. die folgenden Eckdaten überprüft: Demographische Daten, Ein- und Ausschlusskriterien, Einwilligungserklärungen und Übereignungsverträge, AEs, reguläres Ende bzw. ggf. Abbruch der klinischen Prüfung und Abbruchgrund, initialer Koloskopiebefund, histologische Dokumentation der initialen und ggf. folgenden Adenomentfernung und Befund der Abschlußkoloskopie nach 3 Jahren, Probandentagebücher. In der Prüfervereinbarung wird der Zugang des Monitors zu den Studienunterlagen zugesichert. Der genaue Umfang des Monitoring und des Originaldatenabgleichs wird im studienspezifischen Monitoringplan festgelegt.

Außerdem werden 1x/Jahr Prüfertreffen durchgeführt. Da die Studienzentren in der Durchführung von klinischen Studien erfahrene Zentren sind, werden für diese Studie keine eigenen Prüfarztschulungen durchgeführt, sondern jedes Studienzentrum trägt für die Schulung der eigenen Mitarbeiter Sorge.

Folgende Qualitätsindikatoren werden für den Studienablauf festgelegt:

- Einhaltung der Rekrutierungsrate
- Einhaltung der Auswahlkriterien
- Einhaltung des Randomisationsprinzips / der Maskierung
- Einhaltung der protokollgemäßen Behandlung
- Einhaltung der Untersuchungs- und Bewertungstermine

Der Stichprobenumfang der zu überprüfenden Daten enthält:

- die formale Prüfung aller in den Dokumentationsbögen erfassten Merkmale und Messgrößen
- die korrekte Übertragung von Krankenblattdaten in die Dokumentationsbögen (Source Data Verification)

Die Studienlogistik erfolgt durch das KKSH in Zusammenarbeit mit dem Hersteller des Studienpräparates und dem beauftragten Logistikunternehmen. Das KKS steht für etwaige Fragen der Prüfzentren während der gängigen Geschäftszeiten telefonisch zur Verfügung.

13.3 Maßnahmen zur Gewährleistung der Adhärenz und Kohortenpflege

Zur Gewährleistung der Adhärenz wird ein Pill-count durchgeführt. Die Probanden werden aufgefordert, die Medikamentenblister in den entsprechenden Schachteln aufzubewahren und zum nächsten geplanten Studientermin mitzubringen. Daneben führen die Probanden ein Tagebuch, in dem die Einnahme der Studiensubstanz durch Ankreuzen dokumentiert wird. Die Tagebücher werden alle 4 Monate ausgetauscht und auf die Probandencompliance im Prüfzentrum überprüft.

Zur Motivation der Studienteilnehmer und der teilnehmenden Zentren sollen folgende Maßnahmen ergriffen werden:

- Probanden-ID-Karte mit Namen, Probanden-ID, Kontaktdaten des KKSH
- Einrichtung einer kostenlosen 0800-Servicenummer für Rückfragen im KKSH während der üblichen Geschäftszeiten
- Poster und Infobroschüren für die Praxen und teilnehmende Prüfzentren
- Einrichtung einer Website mit Links zu den teilnehmenden Partnern und interaktivem Teil (Probanden können Fragen stellen)
- regelmäßiger Newsletter, in dem Informationen zum Studienverlauf, Zahl der rekrutierten Probanden etc. enthalten sind. Der Newsletter wird, bei entsprechender Einverständniserklärung der Probanden, per E-Mail verteilt.
- Einmal pro Jahr Aussendung eines Incentives (Fahrradflickzeug, Nähzeug, Kugelschreiber o.ä.) mit MIRACLE Logo
- Einmal pro Jahr Verlosung eines Preises (Fahrrad o.ä.).

14 Ethische Belange

14.1 Begutachtung des Prüfplans durch die zuständige Ethik-Kommission

Die Durchführung der Studie geschieht in Übereinstimmung mit der letzten Revision der Deklaration von Helsinki [49]. Die zustimmende Bewertung der Ethik-Kommissionen der Martin-Luther-Universität Halle und der Universität Ulm wurde von der Studienleitung eingeholt. Die weiteren Studienzentren legen die Unterlagen in Zusammenarbeit mit dem KKS Halle den jeweils zuständigen Ethik-Kommissionen vor. Die zustimmende Bewertung bezieht sich auf die Eignung des Prüfplans und der Unterlagen, die benutzt werden sollen, um die Einwilligung nach Aufklärung der Probanden zu erhalten und zu dokumentieren. Der Beginn und die Durchführung der Studie sind an die zustimmende Bewertung der Ethik-Kommission gebunden. Bevor die zuständigen Ethik-Kommissionen ihre zustimmende Bewertung der Abläufe und der Dokumentation dargestellt hat, werden keine Probanden in die Studie aufgenommen.
Die beteiligten Prüfer bestätigen mit ihrer Unterschrift, dass sie den Prüfplan gelesen und verstanden haben und gemäß diesem die Studie durchführen werden.

Gemäß GCP-V§10 sind bei Protokolländerungen die Ethikkommissionen zu informieren, ggf. ist ein erneutes Votum/Genehmigung einzuholen. Votumspflichtige Änderungen dürfen nicht vor der Entscheidung der Ethikkommission umgesetzt werden. Eine Ausnahme besteht hiervon bei Änderungen mit Gefahr im Verzug.

14.2 Aufklärung und Einwilligung der Probanden

Vor Aufnahme in die Studie wird jeder Proband vom behandelnden Arzt über Wesen, Ziele, erwartete Vorteile und mögliche Risiken der Studie informiert. Jeder Proband muss seine schriftliche Einwilligung zur Teilnahme an der Studie erklären. Dem Probanden wird dabei ausreichend Zeit und Gelegenheit gegeben, um vor der Einleitung von Studienmaßnahmen über seine Teilnahme zu entscheiden und offene Fragen zu klären. Die Einwilligungserklärung wird vom Probanden und vom behandelnden Arzt unterzeichnet. Ist der Proband nicht in der Lage, eigenhändig zu unterschreiben, muss ein Zeuge die erfolgte mündliche Aufklärung durch Unterschrift bestätigen.

Ein Muster der Probandeninformation und Einwilligungserklärung sind als Anhang beigefügt. Probandeninformation und Einwilligungserklärung liegen in zweifacher Ausfertigung vor. Ein Exemplar verbleibt beim Prüfer, das andere ist dem Probanden auszuhändigen.

14.3 Verwendung, Speicherung und Weitergabe von Daten

Die Probanden werden darüber informiert, dass ihre krankheitsbezogenen Daten in pseudonymer Form gespeichert und für wissenschaftliche Auswertungen (Publikationen) verwendet werden. Die Probanden haben das Recht, über die gespeicherten Daten informiert zu werden. Für die Probanden ist ersichtlich, wer mit welchem Fokus Einsicht in seine Daten erhält.

15 Gesetzliche und administrative Regelungen

In dieser Studie werden die Empfehlungen der Guten Klinischen Praxis (s. ICH-GCP: International Conference on Harmonisation - Good Clinical Practice), gültig seit dem 17.1.1997, berücksichtigt.

15.1 Versicherung

Es wurde eine freiwillige Probandenversicherung und Wegeversicherung bei der Chubb Insurance Company of Europe abgeschlossen. Die entsprechenden Versicherungsscheine liegen dem Anhang bei.

15.2 Finanzierung

Die Studie wird durch Bereitstellung von Mitteln durch die Deutsche Krebshilfe finanziert. Die Studiensubstanz wird von der Firma Dr. Loges + Co. GmbH, Winsen zur Verfügung gestellt.

15.3 Publikation

Nach Auswertung der Daten ist eine zeitnahe und zügige Publikation der Ergebnisse vorgesehen. Es wird eine Publikation zur Beantwortung der Hauptfragestellung geben (unabhängig davon, wie die Ergebnisse ausfallen). Als Autoren sind beteiligt: Die Studienleitung/Studienkoordination gleichberechtigt, Biometrie, Epidemiologie sowie die Hauptprüfer der Studienzentren soweit sie an der Rekrutierung maßgeblich mitgewirkt haben. Weitere Autoren werden nach ihrem

wissenschaftlichen Input zur Auswertung bzw. bei der Manuskripterstellung aufgenommen. Neben der Hauptfragestellung sind eine Reihe weiterer Publikationen vorgesehen, darunter die Publikationen der explorativen Auswertungen zu Biomarkern, zur Kinetik des Grünteeextraktes und zur histologischen Gewebebank. Die Publikationen werden in Absprache mit der Studienleitung erstellt.

15.4 Einhaltung des Protokolls und Protokolländerungen

Das Studienprotokoll ist genau einzuhalten. Jede vom Prüfer zu vertretende Abweichung von den vorgesehenen Untersuchungs- und Behandlungsmaßnahmen oder -Zeitpunkten ist zu dokumentieren und zu begründen (z. B. Notfallmaßnahmen).

Änderungen oder Ergänzungen des Studienprotokolls können nur vom Studienleiter und Studienkoordinator gemeinsam veranlasst und autorisiert werden.

16 Verzeichnis der Mitwirkenden / Unterschriften

Name	Unterschrift
**Sponsor:**<br><br>Martin-Luther-Universität Halle-Wittenberg vertreten durch den Prodekan für Forschung der Medizinischen Fakultät<br>Prof. Dr. med. Thomas Seufferlein<br>D-06099 Halle (Saale)<br>Tel: +49 (0) 345 557 5420, Fax: +49 (0) 345 557 5424	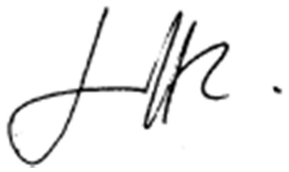
**Studienleiter:**<br><br>Prof. Dr. med. Thomas Seufferlein<br>Direktor der Universitätsklinik und Poliklinik für Innere Medizin I, Martin-Luther-Universität Halle-Wittenberg<br>Ernst-Grube-Straße 40, 06120 Halle (Saale)<br>Tel: +49 (0) 345 557 2661, Fax: +49 (0) 345 557 2253<br>E-Mail: thomas.seufferlein@uk-halle.de	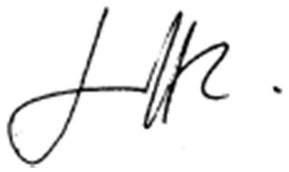
**Studienkoordinatorin:**<br><br>Prof. Dr. med. Julia Stingl<br>Sektion Klinische Pharmakologie, Institut für Naturheilkunde und Klinische Pharmakologie, Universität Ulm<br>Helmholtzstr. 20, 89081 Ulm<br>Tel: +49 (0) 731 500 65603, Fax: +49 (0) 731 500 65605<br>E-mail: julia.stingl@uni-ulm.de	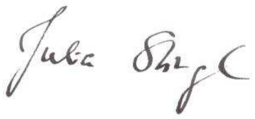
**Biometrie:**<br><br>Prof. Dr. biol. hum. Dipl.-Stat. Rainer Muche<br>Institut für Biometrie, Universität Ulm<br>Schwabstrasse 13, 89075 Ulm<br>Tel: +49 (0) 731 26903, Fax: +49 (0) 731 26902<br>E-mail: rainer.muche@uni-ulm.de	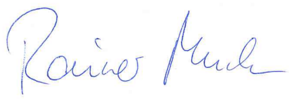
**Epidemiologische Beratung:**<br><br>Prof. Dr. med. Andreas Stang, MPH<br>Institut für Klinische Epidemiologie (IKE)<br>Martin-Luther-Universität Halle-Wittenberg<br>Magdeburger Str. 8, , 06097 Halle (Saale)<br>Tel: +49-(0)345-557-3567, Fax: +49-(0)345-557-3565<br>E-mail: andreas.stang@medizin.uni-halle.de	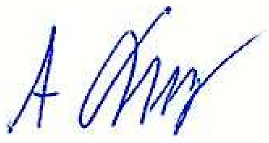
**Studienorganisation / Histologische Gewebebank**<br><br>Dr. med. Thomas J. Ettrich<br>Universitätsklinik und Poliklinik<br>für Innere Medizin I, Universitätsklinikum Halle (Saale)<br>Ernst-Grube-Straße 40, 06120 Halle (Saale)<br>E-mail: thomas.ettrich@medizin.uni-halle.de	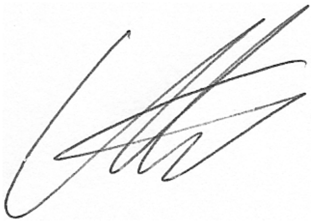

<table>
<tr><th>Name</th><th>Unterschrift</th></tr>
<tr><td>Chemische Analytik / Biomarker Screen
Sektion Klinische Pharmakologie, Institut für Naturheilkunde und Klinische Pharmakologie, Universität Ulm
Helmholtzstr. 20, 89081 Ulm</td><td>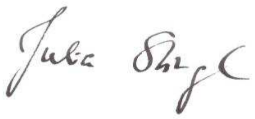</td></tr>
<tr><td>Datenmanagement/Monitoring
Dr. rer. med. Clarissa Dägelmann
Koordinierungszentrum für Klinische Studien der Medizinischen Fakultät der Martin-Luther-Universität Halle-Wittenberg
06097 Halle (Saale)
Tel: +49 (0) 345 557 4908
Fax: +49 (0) 345 557 5210
E-Mail: daegelmann.clarissa@kks-halle.de</td><td>Finanzierung
Deutsche Krebshilfe e.V.
Buschstr. 32, 53113 Bonn
Tel: +49 (0) 228 72990 0
Fax: +49 (0) 228 72990 11
E-Mail: deutsche@krebshilfe.de</td></tr>
<tr><td>Ethik-Kommissionen der Studienleitung
Ethikkommission
der Medizinischen Fakultät
der Martin-Luther-Universität
Halle-Wittenberg
Magdeburger Straße 8,
06112 Halle (Saale)
Tel: +49 (0) 345 557 4476
Fax: +49 (0) 345 557 4477
E-mail: ethik-kommission@medizin.uni-halle.de</td><td>Ethikkommission der Universität Ulm
Helmholtzstraße 20, 89081 Ulm
Tel: +49 (0) 731 500 22052
Fax: +49 (0) 731 500 22036
E-mail: ethik-kommission@uni-ulm.de</td></tr>
</table>

17 Literaturverzeichnis

1. Widlansky ME, Hamburg NM, Anter E, et al. Acute EGCG supplementation reverses endothelial dysfunction in patients with coronary artery disease. J Am Coll Nutr 2007;26:95-102.
2. Kuriyama S, Shimazu T, Ohmori K, et al. Green tea consumption and mortality due to cardiovascular disease, cancer, and all causes in Japan: the Ohsaki study. Jama 2006;296:1255-65.
3. Cabrera C, Artacho R, Gimenez R. Beneficial effects of green tea--a review. J Am Coll Nutr 2006;25:79-99.
4. Thomasset SC, Berry DP, Garcea G, Marczylo T, Steward WP, Gescher AJ. Dietary polyphenolic phytochemicals--promising cancer chemopreventive agents in humans? A review of their clinical properties. Int J Cancer 2007;120:451-8.
5. Shanafelt TD, Call TG, Zent CS, et al. Phase I trial of daily oral Polyphenon E in patients with asymptomatic Rai stage 0 to II chronic lymphocytic leukemia. J Clin Oncol 2009;27:3808-14.
6. Graham HN. Green tea composition, consumption, and polyphenol chemistry. Prev Med 1992;21:334-50.
7. McKay DL, Blumberg JB. The role of tea in human health: an update. J Am Coll Nutr 2002;21:1-13.
8. Nam S, Smith DM, Dou QP. Ester bond-containing tea polyphenols potently inhibit proteasome activity in vitro and in vivo. J Biol Chem 2001;276:13322-30.
9. Baek SJ, Kim JS, Jackson FR, Eling TE, McEntee MF, Lee SH. Epicatechin gallate-induced expression of NAG-1 is associated with growth inhibition and apoptosis in colon cancer cells. Carcinogenesis 2004;25:2425-32.
10. Shimizu M, Deguchi A, Lim JT, Moriwaki H, Kopelovich L, Weinstein IB. (-)-Epigallocatechin gallate and polyphenon E inhibit growth and activation of the epidermal growth factor receptor and human epidermal growth factor receptor-2 signaling pathways in human colon cancer cells. Clin Cancer Res 2005;11:2735-46.
11. Berger SJ, Gupta S, Belfi CA, Gosky DM, Mukhtar H. Green tea constituent (--)-epigallocatechin-3-gallate inhibits topoisomerase I activity in human colon carcinoma cells. Biochem Biophys Res Commun 2001;288:101-5.
12. Jung YD, Kim MS, Shin BA, et al. EGCG, a major component of green tea, inhibits tumour growth by inhibiting VEGF induction in human colon carcinoma cells. Br J Cancer 2001;84:844-50.
13. Peng G, Dixon DA, Muga SJ, Smith TJ, Wargovich MJ. Green tea polyphenol (-)-epigallocatechin-3-gallate inhibits cyclooxygenase-2 expression in colon carcinogenesis. Mol Carcinog 2006;45:309-19.
14. Umeda D, Yano S, Yamada K, Tachibana H. Involvement of 67-kDa laminin receptor-mediated myosin phosphatase activation in antiproliferative effect of epigallocatechin-3-O-gallate at a physiological concentration on Caco-2 colon cancer cells. Biochem Biophys Res Commun 2008;371:172-6.
15. Shammas MA, Neri P, Koley H, et al. Specific killing of multiple myeloma cells by (-)-epigallocatechin-3-gallate extracted from green tea: biologic activity and therapeutic implications. Blood 2006;108:2804-10.
16. Xiao H, Hao X, Simi B, et al. Green tea polyphenols inhibit colorectal aberrant crypt foci (ACF) formation and prevent oncogenic changes in dysplastic ACF in azoxymethane-treated F344 rats. Carcinogenesis 2008;29:113-9.
17. Metz N, Lobstein A, Schneider Y, et al. Suppression of azoxymethane-induced preneoplastic lesions and inhibition of cyclooxygenase-2 activity in the colonic mucosa of rats drinking a crude green tea extract. Nutr Cancer 2000;38:60-4.

18. Ju J, Hong J, Zhou JN, et al. Inhibition of intestinal tumorigenesis in Apcmin/+ mice by (-)-epigallocatechin-3-gallate, the major catechin in green tea. Cancer Res 2005;65:10623-31.

19. Issa AY, Volate SR, Muga SJ, Nitcheva D, Smith T, Wargovich MJ. Green tea selectively targets initial stages of intestinal carcinogenesis in the AOM-ApcMin mouse model. Carcinogenesis 2007;28:1978-84.

20. Yang CS, Maliakal P, Meng X. Inhibition of carcinogenesis by tea. Annu Rev Pharmacol Toxicol 2002;42:25-54.

21. Michels KB, Willett WC, Fuchs CS, Giovannucci E. Coffee, tea, and caffeine consumption and incidence of colon and rectal cancer. J Natl Cancer Inst 2005;97:282-92.

22. Tavani A, La Vecchia C. Coffee, decaffeinated coffee, tea and cancer of the colon and rectum: a review of epidemiological studies, 1990-2003. Cancer Causes Control 2004;15:743-57.

23. Sun CL, Yuan JM, Koh WP, Yu MC. Green tea, black tea and breast cancer risk: a meta-analysis of epidemiological studies. Carcinogenesis 2006;27:1310-5.

24. Bettuzzi S, Brausi M, Rizzi F, Castagnetti G, Peracchia G, Corti A. Chemoprevention of human prostate cancer by oral administration of green tea catechins in volunteers with high-grade prostate intraepithelial neoplasia: a preliminary report from a one-year proof-of-principle study. Cancer Res 2006;66:1234-40.

25. Linden KG, Carpenter PM, McLaren CE, et al. Chemoprevention of nonmelanoma skin cancer: experience with a polyphenol from green tea. Recent Results Cancer Res 2003;163:165-71; discussion 264-6.

26. Li N, Sun Z, Han C, Chen J. The chemopreventive effects of tea on human oral precancerous mucosa lesions. Proc Soc Exp Biol Med 1999;220:218-24.

27. Luo H, Tang L, Tang M, et al. Phase IIa chemoprevention trial of green tea polyphenols in high-risk individuals of liver cancer: modulation of urinary excretion of green tea polyphenols and 8-hydroxydeoxyguanosine. Carcinogenesis 2006;27:262-8.

28. Ahn WS, Yoo J, Huh SW, et al. Protective effects of green tea extracts (polyphenon E and EGCG) on human cervical lesions. Eur J Cancer Prev 2003;12:383-90.

29. Chan AT, Ogino S, Fuchs CS. Aspirin and the risk of colorectal cancer in relation to the expression of COX-2. N Engl J Med 2007;356:2131-42.

30. Baron JA, Cole BF, Sandler RS, et al. A randomized trial of aspirin to prevent colorectal adenomas. N Engl J Med 2003;348:891-9.

31. Baron JA, Sandler RS, Bresalier RS, et al. A randomized trial of rofecoxib for the chemoprevention of colorectal adenomas. Gastroenterology 2006;131:1674-82.

32. Flossmann E, Rothwell PM. Effect of aspirin on long-term risk of colorectal cancer: consistent evidence from randomised and observational studies. Lancet 2007;369:1603-13.

33. Arber N, Eagle CJ, Spicak J, et al. Celecoxib for the prevention of colorectal adenomatous polyps. N Engl J Med 2006;355:885-95.

34. Bertagnolli MM, Eagle CJ, Zauber AG, et al. Celecoxib for the prevention of sporadic colorectal adenomas. N Engl J Med 2006;355:873-84.

35. Shimizu M, Fukutomi Y, Ninomiya M, et al. Green tea extracts for the prevention of metachronous colorectal adenomas: a pilot study. Cancer Epidemiol Biomarkers Prev 2008;17:3020-5.

36. Chow HH, Cai Y, Alberts DS, et al. Phase I pharmacokinetic study of tea polyphenols following single-dose administration of epigallocatechin gallate and polyphenon E. Cancer Epidemiol Biomarkers Prev 2001;10:53-8.

37. Chow HH, Cai Y, Hakim IA, et al. Pharmacokinetics and safety of green tea polyphenols after multiple-dose administration of epigallocatechin gallate and polyphenon E in healthy individuals. Clin Cancer Res 2003;9:3312-9.

38. Ullmann U, Haller J, Decourt JD, Girault J, Spitzer V, Weber P. Plasma-kinetic characteristics of purified and isolated green tea catechin epigallocatechin gallate (EGCG) after 10 days repeated dosing in healthy volunteers. Int J Vitam Nutr Res 2004;74:269-78.
39. Ullmann U, Haller J, Decourt JP, et al. A single ascending dose study of epigallocatechin gallate in healthy volunteers. J Int Med Res 2003;31:88-101.
40. Schmiegel W, Reinacher-Schick A, Arnold D, et al. [Update S3-guideline "colorectal cancer" 2008]. Z Gastroenterol 2008;46:799-840.
41. Schoen RE, Pinsky PF, Weissfeld JL, et al. Results of repeat sigmoidoscopy 3 years after a negative examination. Jama 2003;290:41-8.
42. Sarma DN, Barrett ML, Chavez ML, et al. Safety of green tea extracts : a systematic review by the US Pharmacopeia. Drug Saf 2008;31:469-84.
43. Swezey RR, Aldridge DE, LeValley SE, Crowell JA, Hara Y, Green CE. Absorption, tissue distribution and elimination of 4-[(3)h]-epigallocatechin gallate in beagle dogs. Int J Toxicol 2003;22:187-93.
44. Galati G, Lin A, Sultan AM, O'Brien PJ. Cellular and in vivo hepatotoxicity caused by green tea phenolic acids and catechins. Free Radic Biol Med 2006;40:570-80.
45. Pisters KM, Newman RA, Coldman B, et al. Phase I trial of oral green tea extract in adult patients with solid tumors. J Clin Oncol 2001;19:1830-8.
46. Lee MJ, Maliakal P, Chen L, et al. Pharmacokinetics of tea catechins after ingestion of green tea and (-)-epigallocatechin-3-gallate by humans: formation of different metabolites and individual variability. Cancer Epidemiol Biomarkers Prev 2002;11:1025-32.
47. Yang CS, Chen L, Lee MJ, Balentine D, Kuo MC, Schantz SP. Blood and urine levels of tea catechins after ingestion of different amounts of green tea by human volunteers. Cancer Epidemiol Biomarkers Prev 1998;7:351-4.
48. Jodoin J, Demeule M, Beliveau R. Inhibition of the multidrug resistance P-glycoprotein activity by green tea polyphenols. Biochim Biophys Acta 2002;1542:149-59.
49. World Medical Association Declaration of Helsinki: ethical principles for medical research involving human subjects. JAMA : the journal of the American Medical Association 2000;284:3043-5.
50. Oken MM, Creech RH, Tormey DC, et al. Toxicity and response criteria of the Eastern Cooperative Oncology Group. Am J Clin Oncol 1982;5:649-55.

18 Appendices

18.1 Probandeninformation MIRACLE-Studie

UKH Universitätsklinikum Halle (Saale)

gefördert durch Deutsche Krebshilfe HELFEN. FORSCHEN. INFORMIEREN.

ulm university universität ulm

Department für Innere Medizin
Universitätsklinik und Poliklinik
für Innere Medizin I
Ernst-Grube-Straße 40
06120 Halle (Saale)
Tel. 0345/5570
Direktor:
Prof. Dr. med. Th. Seufferlein

Institut für Naturheilkunde und
Klinische Pharmakologie der
Universität Ulm
Helmholtzstr. 20
89081 Ulm
Prof. Dr. med. J. Stingl

Probandeninformation

MIRACLE-Studie

Minimizing the Risk of Metachronous Adenomas of the Colorectum with Green Tea Extract

Präventionsstudie zu Grünteeextrakt als Nahrungsergänzungsmittel zur Vermeidung metachroner kolorektaler Adenome

Sehr geehrte Dame, sehr geehrter Herr,

bei Ihnen ist in den letzten sechs Monaten eine Dickdarmspiegelung durchgeführt worden, bei der Adenome entfernt wurden. Wir wissen, dass Patienten, bei denen Dickdarmadenome festgestellt wurden, auch nach deren Entfernung ein höheres Risiko haben, wieder Polypen zu entwickeln. Dickdarmadenome können über die Zeit hin zu bösartigem Zellwachstum führen und sich zu Darmkrebs entwickeln. Da diese Entwicklung langsam vonstatten geht und auch nur bei einem kleinen Teil der Polypen beobachtet wird, erscheint es nach der heutigen Studienlage ausreichend, nach einigen Jahren (in der Regel 3 Jahren, manchmal auch früher oder später) wiederum eine Dickdarmspiegelung durchzuführen und gegebenenfalls wiederum Polypen zu entfernen.

Grüner Tee gehört zu den ältesten Getränken der Welt und bereits im antiken China schrieb man den Inhaltsstoffen der grünen Teeblätter heilende Eigenschaften zu. Inzwischen gibt es eine Vielzahl von Forschungsarbeiten, die die Wirkung der Inhaltsstoffe von Grüntee und seinem Extrakt auf unterschiedliche Erkrankungen untersucht haben. So hat Grüntee nachgewiesenermaßen blutzuckersenkende Eigenschaften, es wirkt gegen Entzündungen und jüngst wurde auch eine Wirkung auf krankheitsbedingte fibrillenartige Ablagerungen im Gehirn beschrieben. Besonders gut untersucht aber sind die das Krebswachstum hemmenden Eigenschaften von Grüntee. Es gibt große Bevölkerungsstudien, besonders aus asiatischen Ländern wo viel Grüntee getrunken wird, die ein niedrigeres Vorkommen bestimmter Krebsarten bei Personen, die viel Grüntee trinken, zeigen. Besser untersucht sind die krebshemmenden Eigenschaften allerdings in gezielten klinischen Studien an Tieren oder auch bereits bei Patienten. Dabei scheint Grüntee insbesondere vorbeugende und krebswachstumshemmende Eigenschaften zu besitzen. So wurde ein selteneres Auftreten von Prostatakrebs bei Patienten mir hohem Risiko für diese Erkrankung gezeigt. Bei Frauen wurde ein Rückgang der Zellveränderungen bei Vorstufen von Gebärmutterhalskrebs verzeichnet und zwar sowohl durch lokale Wirkung von Grünteeextrakt, als auch nach Einnahme von Grünteeextraktkapseln.

Grünteeextrakt zeichnet sich dadurch aus, dass er die wichtigen und wirksamen Inhaltsstoffe des Grüntees, die Katechine (eine Art Gerbstoffe), in festgelegter und gleichbleibender Konzentration enthält und dass im Extrakt etwa 6-10-fach soviel an Inhaltsstoffen enthalten ist. Außerdem muss das im grünen Tee enthaltene Koffein entfernt werden, so dass Grünteeextrakt keine nennenswerten Mengen von Koffein enthält.

Aufgrund dieser Eigenschaften sind die meisten Studien beim Menschen mit Grünteeextrakt Kapseln und nicht mit Grünteegetränk durchgeführt worden. Insbesondere die Menge an Grüntee, die man trinken müsste, um ausreichend hohe Wirkspiegel zu erreichen, macht Studien mit Teegetränk schwer, entspricht doch eine Grünteekapsel mit 300 mg des wirksamen Hauptinhaltsstoffes Epigallokatechingallat etwa 7-9 Tassen Tee.
In dieser Studie soll untersucht werden, inwieweit Grünteeextrakt in Kapselform täglich zu sich genommen, das Wiederauftreten von Darmpolypen verhindern kann. An einem bestimmten Mäusestamm, bei dem aufgrund einer genetischen Veränderung in hohem Maße Darmpolypen und Tumore wachsen, wurde unter Grünteeextrakt ein dramatisches Absinken der Tumorbildung beobachtet. Molekulare Untersuchungen haben dann gezeigt, dass die Inhaltsstoffe des Grüntees genau die Signalwege angreifen und verbessern, die bei der Entstehung von Darmkrebs gestört oder verändert sind.
In dieser Studie erhalten Sie über drei Jahre hinweg, also in der Regel bis zur nächsten geplanten Darmspiegelung, täglich zwei Kapseln, die jeweils 150 mg Katechine aus Grünteeextrakt enthalten oder ein sogenanntes Placebo. Da es bisher keine wirksame - und gleichzeitig nebenwirkungsarme - vorbeugende Therapie von Dickdarmpolypen gibt, haben Sie, falls Sie in die Placebogruppe gelangen, keinen Nachteil, sondern erfahren lediglich die derzeit üblichen Maßnahmen, nämlich eine Kontrolle der Dickdarmspiegelung nach in der Regel 3 Jahren.
Sie sollten, wenn Sie an dieser Studie teilnehmen, auf das regelmäßige Trinken von grünem Tee verzichten, da bei Dosierungen von über 300 mg Grünteeinhaltsstoffen pro Tag, d.h. über der Dosis des hier verwendeten Studienpräparates, Nebenwirkungen wie z.B. Leberwerterhöhungen auftreten können. Wir bitten Sie, die Einnahme von Medikamenten, die eine vorbeugende Wirkung gegenüber dem Wiederauftreten von Dickdarmpolympen haben, wie Aspirin und ähnliche Schmerzmedikamente oder Statine (Cholesterinsenker) zu dokumentieren.
Alle vier Monate, während der Dauer der Studie, müssten Sie in der Praxis erscheinen und es werden Befragungen zur regelmäßigen Einnahme der Studienmedikation, sowie zu weiterer Medikamenteneinnahme, zu Grüntee-Konsum und ärztlichen Behandlungen durchgeführt. Zu Studienbeginn, 1 Monat nach Studienbeginn, nach 4, 12, 20 und 36 Monaten wird eine Blutentnahme zur Kontrolle von Leberwerten durchgeführt.
Es wird außerdem nach 1, 4, 20 und 36 Monaten eine Blutentnahme vorgenommen, um Blut für genetische und biochemische Untersuchungen zu Faktoren, die eine Bedeutung für das Wiederauftreten von Dickdarmpolypen haben können, zu gewinnen (nur bei Teilnahme am

Zusatzprojekt „Biomarker und Nutrigenetik“). Weiterhin wird in einem Zusatzprojekt das entnommene Gewebe ihres Darmpolypen, sofern es sich dabei um fortgeschrittene Polypen (d. h. Adenome mit hochgradiger Dysplasie oder serratierte Adenome) handelt, zentral in einer Biomaterialbank gesammelt und auf Veränderungen des Gewebes hinsichtlich Veränderungen zu bösartigem Tumorgewebe untersucht (Mutationen, Veränderungen der intrazellulären Signalwege, Eigenschaften der Zellen). Diese Materialien werden pseudonymisiert ohne Rückverfolgung auf Ihre Person analysiert und für alle an dieser Studie teilnehmenden Probanden ausgewertet. Sie können an der eigentlichen Studie zu Grünteeextrakt auch dann teilnehmen, wenn Sie an diesen Zusatzprojekten zu Biomarkern, Pharmakogenetik und histologischen Untersuchungen des entnommenen Gewebes des Dickdarmpolypen nicht teilnehmen möchten. Sie helfen aber durch Ihre Teilnahme, wichtige Erkenntnisse zu individuellen Risikofaktoren für das Wiederauftreten von Dickdarmadenomen zu gewinnen, die in Zukunft anderen Patienten zugute kommen können.

Kontrolluntersuchungen des Dickdarms (Koloskopien) werden wie von Ihrem behandelnden Arzt empfohlen durchgeführt, bei manchen Patienten ist dies jährlich, in der Regel jedoch erst im Abstand von 3 Jahren erforderlich. Es wird keine zusätzliche Dickdarmspiegelung durch die Studie veranlasst, sondern bei den routinemäßig durchgeführten Kontrolluntersuchungen wird das eventuelle Wiederauftreten von Dickdarmpolypen erhoben. Bei den Kontrolluntersuchungen des Dickdarms wird die Größe, die genaue Lage aller Adenome oder sonstiger Schleimhautläsionen im Darm erhoben und die Befunde von Gewebeproben und Polypenabtragungen dokumentiert. Zur Auswertung wird das Wiederauftreten von Dickdarm-Polypen innerhalb des Beobachtungszeitraumes von 3 Jahren verwendet.

Zeitplan der studienbedingten Aktionen:

Studienbeginn:

Die Studie beginnt mit Ihrem schriftlichen Einverständnis maximal 6 Monate nach der erstmaligen Dickdarmspiegelung mit Entfernung von Adenomen. Zu Beginn werden einige Fragen zu ihrem Gesundheitszustand, ihrer derzeitigen Medikation und zu Lebensgewohnheiten gestellt. Zu diesem Zeitpunkt erfolgt eine Blutentnahme zur Bestimmung der Leberwerte. Ab diesem Zeitpunkt erhalten Sie zunächst für einen Monat als sogenannte Run-In Periode die Studiensubstanz zur täglichen Einnahme von jeweils zwei Kapseln. Diese Run-In Periode dient dazu, Erfahrung zu gewinnen, wie Sie mit der täglichen Einnahme zurecht kommen, aber auch,

um eventuelle Unverträglichkeitsreaktionen auf Grünteeextrakt rechtzeitig erkennen zu können. Am Ende des Monats vereinbaren Sie ein Treffen mit Ihrem Prüfarzt und bringen dazu den leeren Kapselbehälter bzw. alle noch übriggebliebenen Kapseln mit. Es werden nochmals die Leberwerte kontrolliert und wenn Sie mit der Einnahme der Kapseln gut zurecht kamen und Ihre Leberwerte im Normbereich liegen, erhalten Sie von da ab die Studiensubstanz, die individuell für Ihre Teilnehmernummer abgefüllt wurde. Die Teilnehmernummer bleibt über den gesamten Studienzeitraum gleich. Zur Vereinfachung bekommen Sie einen Probandenausweis mit Ihrem Namen und Ihrer persönlichen Teilnehmernummer. Sie bekommen die Kapseln im voraus für den Zeitraum von vier Monaten mit nach Hause. Zusammen mit den Kapseln erhalten Sie auch ein Probandentagebuch, in das Sie die regelmäßige Einnahme, sowie aufgetretene Beschwerden und etwaige Besonderheiten eintragen sollten. Bitte kontrollieren Sie jedes Mal bei Erhalt von neuer Studiensubstanz, dass Ihre Teilnehmernummer auf den Behältnissen die gleiche wie auf Ihrem Tagebuch ist. Dieses Tagebuch ermöglicht Ihnen und den Studienmitarbeitern, einen genauen Überblick über Ihr Befinden während des Studienzeitraumes und gibt einen Überblick über die Anzahl an Kapseln, die Sie eingenommen haben. Bitte geben Sie nie Kapseln an andere Personen weiter oder nehmen Kapseln ein, die für einen anderen Studienteilnehmer bestimmt sind.

Termine im Rahmen der Studie:

Der erste Termin findet einen Monat nach Beginn der Einnahme der Studiensubstanz während der Run-In Phase (s.o.) statt. Hier erfolgt eine Laborkontrolle der Leberwerte. 4 Monate nach Studienbeginn ist der nächste Termin, bei dem Laborwerte kontrolliert werden, die u.U. eine Unverträglichkeit gegenüber Grünteeextrakt zeigen könnten (Leberwerte). Außerdem erhalten Sie die Studiensubstanz (Kapseln) für die nächsten 4 Monate und ein neues Probandentagebuch.

Nach diesem ersten Arztbesuch nach 4 Monaten vereinbaren sie bitte weitere Termine im Abstand von 4 Monaten zum Austausch des Probandentagebuchs und zum Erhalt neuer Studiensubstanz für die nächsten 4 Monate. Dies geschieht am einfachsten durch einen Besuch bei Ihrem behandelnden Gastroenterologen, der die Koloskopie durchgeführt hat. Bitte bringen sie die leeren Medikamentenblister sowie alle darin befindliche, nicht verbrauchte Studiensubstanz bei diesen Besuchen mit.

Blutentnahmen zur Kontrolle der Leberwerte (ca. 3ml) sind nach 1, 4, 12, 20 und 36 Monaten vorgesehen. Blutentnahmen (jeweils 14 ml) zur Einlagerung in die Biobank werden nach 4, 20 und 36 Monaten vorgenommen.

Studienabschluss:

Die Studie endet mit der Kontrollkoloskopie 3 Jahre nach der ersten Koloskopie (36 Monate nach Einschluss). Dazu werden Sie im Vorfeld von Ihrem Arzt kontaktiert, um einen Termin zu vereinbaren. Bei diesem Termin bringen Sie das letzte Probandentagebuch sowie ggf. nicht verbrauchte Studiensubstanz (Kapseln) mit. Es wird zum Abschluss noch eine letzte Blutentnahme durchgeführt, um Leberwerte, sowie biochemische Parameter (nur bei Teilnahme am Zusatzprojekt „Biomarker und Nutrigenetik“), die für die Dickdarmpolypenentstehung wichtig sein könnten, zu bestimmen.

Sicherheit und Risiken:

Grünteeextrakt und dessen Hauptinhaltsstoff EGCG wird als gut verträgliches Produkt beschrieben, welches keine besonderen Nebenwirkungen hat. In Studien an Probanden oder Patienten traten insbesondere Beschwerden wie Blähungen, Dyspepsie, Übelkeit, sowie Unruhe, Schwindel und Schlaflosigkeit auf, die dem 7% Gehalt an Koffein der in diesen Studien verwendeten Extrakte zuzuschreiben sind. In dieser Studie wird entkoffeinierter Grüntee-Extrakt verwendet, weshalb mit diesen Beschwerden nicht zu rechnen ist.

In einer Studie zur Vorbeugung von Prostatakrebs bei Männern wurde in der Gruppe, die mit Grünteeextrakt behandelt wurde, etwas häufiger über Durchfälle berichtet, als in der Vergleichsgruppe. Die Dosis des Wirkstoffes EGCG lag allerdings in dieser Studie doppelt so hoch wie die Dosis, die hier in dieser Studie verwendet wird.

In Einzelfällen wurde über das Auftreten von Leberwerterhöhungen im Zusammenhang mit grünteehaltigen Nahrungsmitteln und Nahrungsergänzungsmitteln berichtet. Von insgesamt 216 Berichten über unerwünschte Ereignisse im zeitlichen Zusammenhang mit der Einnahme von grünteehaltigen Nahrungsmitteln wurde in 7 Fällen ein kausaler Zusammenhang zwischen einer Leberwerterhöhung und der Einnahme von Grünteeextrakt hergestellt. Beim Menschen wurde gezeigt, dass eine Einnahme von Grünteeextrakt auf nüchternen Magen zu fünffach höheren Blutkonzentrationen von EGCG führen kann, als eine Einnahme mit dem Essen. Daher sollen Sie die Studiensubstanz unbedingt zu einer Mahlzeit, am besten mit dem Frühstück und dem Abendessen einnehmen. Außerdem bitten wir Sie, bei möglichen Zeichen einer Lebererkrankung, wie Oberbauchschmerzen und einer Gelbfärbung der weißen Teile im Auge (der Skleren) oder

einer Dunkelfärbung des Urins, sofort Kontakt mit dem jeweiligen Arzt des Studienzentrums aufzunehmen.

Generell sollten Sie sich bei allen Fragen, sowie plötzlich auftretenden Beschwerden oder anderen Problemen, die das Fortführen der Studie beeinflussen können, mit Ihrem behandelnden Arzt in Verbindung setzen. Sie erhalten dafür eine Telefonnummer des betreuenden Arztes in Ihrem zuständigen Studienzentrum. Für den Fall, dass in Ihrem Studienzentrum niemand erreichbar sein sollte, erhalten sie eine Telefonnummer und eine E-Mail-Adresse über die Sie Mitarbeiter des Koordinierungszentrums für Klinische Studien in Halle (Saale) erreichen können.

Einschlusskriterien

An dieser Studie können Sie teilnehmen, wenn Sie zwischen 50 und 80 Jahre alt sind und erstmalig bei einer Dickdarmuntersuchung in den letzten 6 Monaten Dickdarmpolypen entfernt bekommen haben.

Bei dieser Studie dürfen Sie nicht teilnehmen, wenn Sie an einer Krebserkrankung des Dickdarms leiden, wenn Sie Erkrankungen haben, die die normale Aufnahme von Tabletten oder Nahrungsmittel beeinflussen (sogenannte Malabsorptionssyndrome oder Kurzdarmsyndrom nach chirurgischen Eingriffen), wenn sie Kapseln nicht schlucken können oder bekanntermaßen auf Grünen Tee allergisch sind, wenn bei Ihnen eine Erkrankung der Leber oder des Darmes besteht (aktive chronische oder akute Hepatitis, Leberzirrhose, sonstige Gelbsucht, chronisch entzündliche Darmerkrankungen) oder wenn eine längerfristige Einnahme von Schmerzmitteln aus der Gruppe der nichtsteroidalen Antirheumatika, einschließlich Acetylsalicylsäure (ASS) > 100 mg/d, oder der Coxibe notwendig ist. Sollte die Einnahme derartiger Medikamente während des Studienverlaufs bei ihnen notwendig werden, informieren sie bitte umgehend das Studienzentrum, da evtl. eine Beendigung der Studienteilnahme notwendig sein kann. Außerdem dürfen Sie nicht teilnehmen, wenn Sie bereits seit Jahren regelmäßig Kapseln mit Grünteeextrakt einnehmen.

Freiwilligkeit:

An diesem Forschungsprojekt nehmen Sie freiwillig teil. Ihr Einverständnis können Sie jederzeit und ohne Angabe von Gründen widerrufen. Dieser eventuelle Widerruf hat keine Auswirkungen auf Ihre medizinische Betreuung.

Versicherung:

Für die Teilnahme an diesem Forschungsprojekt genießen Sie Versicherungsschutz (Haftpflichtversicherung und Wegunfallversicherung). Es gelten die allgemeinen Haftungsbedingungen der jeweiligen Versicherung. Die Versicherungen wurde abgeschlossen bei der “Chubb Insurance Company of Europe SE”.

Schweigepflicht/Datenschutz:

Alle Personen, welche Sie im Rahmen dieses Projektes betreuen, unterliegen der Schweigepflicht und sind dem Datengeheimnis verpflichtet.

Die studienbezogenen Untersuchungsergebnisse werden nur in anonymisierter Form in wissenschaftlichen Veröffentlichungen verwendet. Die Speicherung und Auswertung der Untersuchungsergebnisse erfolgt in pseudonymisierter Form, dass heißt, dass personenbezogene Identifikationsmerkmale (z.B. Namen) durch einen Code ersetzt werden. Damit wird gewährleistet, dass die Identifikation oder Zuordnung einer Probe zu einer bestimmten Person unmöglich bzw. deutlich erschwert ist. Soweit es zur Kontrolle der korrekten Datenerhebung erforderlich ist, dürfen autorisierte Personen (z.B. autorisierte Mitarbeiter der Universität) Einsicht in die studienrelevanten Teile der Krankenakte nehmen. Die zur Einsichtnahme autorisierten Personen unterliegen ebenfalls der Schweigepflicht und dem Datengeheimnis.

18.2 Probandeninformation Zusatzprojekt „Biomarker und Nutrigenetik“

Zusatzprojekt „Biomarker und Nutrigenetik“

Sehr geehrte Dame, sehr geehrter Herr,

im Rahmen der M.I.R.A.C.L.E-Studie werden zusätzlich Faktoren untersucht, die eine Bedeutung für die Entwicklung und das Wiederauftreten von Dickdarmpolypen haben. Des Weiteren sollen weiterführende Untersuchungen über das Verhalten und die Wirkung von Grünteeextrakt im menschlichen Körper durchgeführt werden. Bei den routinemäßigen Blutabnahmen im Rahmen der Studienteilnahme wird die Verträglichkeit der Studienmedikation überwacht. Im Rahmen dieser Blutabnahmen werden zusätzliche Blutproben entnommen. Diese zusätzlichen Blutproben werden unter anderem dazu benutzt um menschliche Erbinformation (DNA-Proben) sowie Blutserum und Blutplasma zu gewinnen. An diesem Material sollen biochemische Verlaufsparameter sowie populationsbezogene Biomarker untersucht werden, z.B. spezielle genetische Besonderheiten, welche die Entstehung von Dickdarmpolypen begünstigen und beeinflussen. Des Weiteren werden pharmakogenetische Untersuchungen durchgeführt, dabei werden z.B. genetische Einflüsse auf den Metabolismus und die Wirkung von Grüntee-Extrakt im menschlichen Körper untersucht.

Diese Biomaterialproben werden in eine Biomaterialprobenbank eingefügt und für in Zukunft zu Verfügung stehende Untersuchungsmethoden und krankheitsbezogene Fragestellungen, welche aktuell noch nicht spezifiziert werden können, aufbewahrt. Alle zukünftigen Forschungsprojekte werden jedoch von der zuständigen Ethikkommission auf ihre ethische Unbedenklichkeit eingehend geprüft.

Die Aufbewahrungszeit der Proben beträgt maximal 20 Jahre, anschließend werden die Proben vernichtet. Die Proben gehen in das Eigentum der Universität Halle über. Die Lagerung und Untersuchung der Proben erfolgt in pseudonymisierter Form, dass heißt, dass personenbezogene Identifikationsmerkmale (z.B. Namen) durch einen Code ersetzt werden. Damit wird gewährleistet, dass die Identifikation und Zuordnung einer bestimmten Probe zu einer bestimmten Person unmöglich bzw. deutlich erschwert ist. Eine kommerzielle Verwendung oder eine Weitergabe der Proben an Dritte ist ausgeschlossen.

Bei einem Rücktritt des Probanden von der Studienteilnahme werden die gewonnenen Proben des Probanden vernichtet.

Sie haben die Möglichkeit, an der klinischen Prüfung teilzunehmen. Sie können jedoch die Datennutzung und Probenahme für eine Biomaterialbank ablehnen.

18.3 Probandeninformation Zusatzprojekt „Histologische Gewebebank“

Zusatzprojekt „Histologische Gewebebank“

Sehr geehrte Dame, sehr geehrter Herr,

Dickdarmpolypen kommen in sehr unterschiedlichen Formen vor. Diese Formen unterscheiden sich hinsichtlich ihres Gewebes und dem Grad ihrer Entartung und Entwicklung von der normalen Dickdarmschleimhaut. Ein Teil dieser Dickdarmpolypen gelten als nahe Vorstufen von bösartigem Dickdarmkrebs und werden als fortgeschrittene Polypen bezeichnet. Insbesondere sind dies die Adenome mit bereits hochgradigen Gewebeveränderungen (hochgradige Dysplasie) und sogenannte serratierte Adenome. Diese Arten von Dickdarmpolypen entwickeln erwartungsgemäß nur wenige Studienteilnehmerinnen und Studienteilnehmer. Bei diesen Personen soll im Rahmen dieses Zusatzprojektes im Rahmen der MIRACLE-Studie das Gewebe dieser fortgeschrittenen Dickdarmpolypen, die Ihnen im Rahmen der Koloskopien (Darmspiegelung) entfernt wurden, zentral gesammelt und speziell histologisch und immunhistochemisch untersucht werden. Dabei sollen unter anderem Oberflächeneigenschaften der Zellmembran und das Vorliegen von Mutationen im Erbgut der Zellen des Polypen untersucht werden, die für die Zellteilung und Übertragung von Informationen innerhalb der Zelle verantwortlich sind (intrazelluläre Signalwege). Häufig finden sich an diesen Stellen bei Dickdarmpolypen Veränderungen die auch bei Zellen von bösartigen Dickdarmtumoren (Darmkrebs) auftreten. Für Sie entsteht bei dem Zusatzprojekt kein Aufwand, da das entsprechende Gewebe bereits bei ihrer Dickdarmpolypen-Entfernung im Rahmen der Darmspiegelung routinemäßig entnommen und histologisch begutachtet wird.

Ihre Gewebeproben werden in eine Biomaterialbank eingefügt und für in Zukunft zu Verfügung stehende Untersuchungsmethoden und krankheitsbezogene Fragestellungen, welche aktuell noch nicht spezifiziert werden können, aufbewahrt. Alle zukünftigen Forschungsprojekte werden jedoch von der zuständigen Ethikkommission auf ihre ethische Unbedenklichkeit eingehend geprüft.

Die Gewebeproben gehen in das Eigentum des Universitätsklinikums Halle über und werden grundsätzlich dort gelagert. Die Lagerung und Untersuchung der Gewebeproben erfolgt in pseudonymisierter Form, dass heißt, dass die personenbezogenen Identifikationsmerkmale (z.B. Namen) durch einen Code ersetzt werden. Damit wird gewährleistet, dass die Identifikation und

Zuordnung einer bestimmten Probe zu einer bestimmten Person unmöglich bzw. deutlich erschwert ist. Eine kommerzielle Verwendung oder eine Weitergabe der Proben an Dritte ist ausgeschlossen.

Bei einem Rücktritt des Probanden von der Studienteilnahme werden die gewonnenen Proben des Probanden an den ursprünglich begutachtenden Pathologen zurückversandt. Sie haben die Möglichkeit, an der klinischen Prüfung teilzunehmen. Sie können jedoch die Nutzung Ihrer Gewebeproben für die histologische Gewebebank ablehnen.

18.4 Einwilligungserklärung

Einwilligungserklärung

MIRACLE-Studie

Inhalt, Vorgehensweise, Risiken und Ziel des obengenannten Forschungsprojektes sowie die Befugnis zur Einsichtnahme in die erhobenen Daten hat mir

Dr. med. ________________________________ ausreichend erklärt.

Ich hatte Gelegenheit Fragen zu stellen und habe hierauf Antworten erhalten. Ich hatte ausreichend Zeit, mich für oder gegen die Teilnahme am Projekt zu entscheiden. An diesem Forschungsprojekt nehme ich freiwillig teil. Mein Einwilligung kann ich jederzeit und ohne Angabe von Gründen widerrufen. Es werden dann sämtliche Proben und Daten vernichtet. Dieser eventuelle Widerruf hat keine Auswirkungen auf meine medizinische Betreuung.

Eine Kopie der Probandeninformation und Einwilligungserklärung habe ich erhalten. Ich willige in die Teilnahme am Forschungsprojekt ein.

Ich willige ebenfalls in die Teilnahme an den Zusatzprojekten:
„Biomarker und Pharmakogenetik“ und „Histologische Gewebebank“ ein.
(Bei Nichtzutreffen bitte entsprechendes durchstreichen)

____

Name der Probandin / des Probanden

______________________________	______________________________
Ort, Datum	Ort, Datum
______________________________	______________________________
Unterschrift des Prüfarztes	Unterschrift Probandin / Proband

18.5 Information und Einwilligungsserklärung zum Datenschutz

Information und Einwilligungserklärung zum Datenschutz

Bei wissenschaftlichen Studien werden persönliche Daten und medizinische Befunde über Sie erhoben. Die Weitergabe, Speicherung und Auswertung dieser studienbezogenen Daten erfolgt nach gesetzlichen Bestimmungen und setzt vor Teilnahme an der Studie folgende freiwillige Einwilligung voraus:

Ich erkläre mich damit einverstanden, dass im Rahmen dieser Studie erhobene Daten/Krankheitsdaten auf Fragebögen und elektronischen Datenträgern aufgezeichnet und ohne Namensnennung verarbeitet werden.

Außerdem erkläre ich mich damit einverstanden, dass eine autorisierte und zur Verschwiegenheit verpflichtete Person (z.B.: des Auftraggebers, der Universität) in meine erhobenen personenbezogenen Daten Einsicht nimmt, soweit dies für die Überprüfung des Projektes notwendig ist. Für diese Maßnahme entbinde ich den Arzt von der ärztlichen Schweigepflicht.

________________________________ ________________________________

Ort, Datum Unterschrift Probandin / Proband

Ich erkläre mich damit einverstanden, dass meine E-Mail-Adresse für Informationszwecke (z.B. Erinnerungen an den nächsten Termin im Prüfzentrum) an das Koordinierungszentrum für Klinische Studien der Universität Halle-Wittenberg weitergegeben wird. Eine Weitergabe meiner E-Mail-Adresse oder die Verwendung für kommerzielle Zwecke ist ausgeschlossen.

________________________________ ________________________________

Ort, Datum Unterschrift Probandin / Proband

18.6 Übereignungsvertrag Zusatzprojekt „Biomarker und Nutrigenetik“

Übereignungsvertrag
Zusatzprojekt „Biomarker und Nutrigenetik“

Zwischen der Martin-Luther-Universität Halle-Wittenberg,
handelnd für die Universitätsklinik und Poliklinik für Innere Medizin I
vertreten durch den Ärztlichen Direktor Herrn Prof. Dr. med. Th. Seufferlein
und

____
Name der Probandin / des Probanden

wird folgendes vereinbart:

Das Eigentum an folgendem Körpermaterial wird der Martin-Luther-Universität Halle Wittenberg unentgeltlich übertragen:

63 ml Vollblut (14 ml zu den Zeitpunkten Ende Monat 1, 4, 20, 36 Monate zur Gewinnung von Plasma und Serum und 7 ml zum Zeitpunkt Ende Monat 1 für die Gewinnung von DNA).

- Die Universität wird das Körpermaterial lediglich zu krankheitsbezogenen, medizinisch-wissenschaftlichen Zwecken, und zur biochemischen Verlaufsparameteruntersuchung sowie zur pharmakogenetischen Untersuchung im Rahmen der Studie *Mi*nimizing the *R*isk of Metachronous Adenomas of the *Co*lorectum with Green Tea *E*xtract- die *M.I.R.A.C.L.E.* Studie verwenden.

- Eine kommerzielle Verwendung oder die Weitergabe an Dritte ist ausgeschlossen.

- Die studienbezogenen Untersuchungsergebnisse werden nur in anonymisierter Form in wissenschaftlichen Veröffentlichungen verwendet. Die Speicherung und Auswertung der Untersuchungsergebnisse erfolgt in pseudonymisierter bzw. anonymisierter Form. Mit der EDV-mäßigen Speicherung und Verarbeitung, der bei mir im Rahmen des obengenannten Vertrages erhobenen Daten, bin ich einverstanden.

- Die Proben werden für maximal 20 Jahre aufgehoben und anschließend vernichtet.

______________________________ Ort, Datum	______________________________ Ort, Datum
______________________________ Unterschrift des Prüfarztes	______________________________ Unterschrift Probandin / Proband

18.7 Übereignungsvertrag Zusatzprojekt „Histologische Gewebebank“

Übereignungsvertrag
Zusatzprojekt „Histologische Gewebebank“

Zwischen der Martin-Luther-Universität Halle-Wittenberg,
handelnd für die Universitätsklinik und Poliklinik für Innere Medizin I
vertreten durch den Ärztlichen Direktor Herrn Prof. Dr. med. Th. Seufferlein
und

____
Name der Probandin / des Probanden

wird folgendes vereinbart:

Das Eigentum an folgendem Körpermaterial wird der Martin-Luther-Universität Halle Wittenberg unentgeltlich übertragen:

Histologische Gewebe (in Paraffin konserviert) der im Rahmen der Koloskopien (Darmspiegelungen) entfernten fortgeschrittenen Dickdarmpolypen

- Die Universität wird das Körpermaterial lediglich zu krankheitsbezogenen, medizinisch-wissenschaftlichen Zwecken (immunhistochemischen Untersuchungen, genetischen Untersuchungen) im Rahmen der Studie *Mi*nimizing the *R*isk of Metachronous Adenomas of the *Col*orectum with Green Tea *E*xtract- die *M.I.R.A.C.L.E.* Studie verwenden.

- Eine kommerzielle Verwendung oder die Weitergabe an Dritte ist ausgeschlossen.

- Die studienbezogenen Untersuchungsergebnisse werden nur in anonymisierter Form in wissenschaftlichen Veröffentlichungen verwendet. Die Speicherung und Auswertung der Untersuchungsergebnisse erfolgt in pseudonymisierter bzw. anonymisierter Form. Mit der EDV-mäßigen Speicherung und Verarbeitung, der bei mir im Rahmen des obengenannten Vertrages erhobenen Daten, bin ich einverstanden.

- Die Proben werden für maximal 20 Jahre aufgehoben und anschließend vernichtet.

______________________________	______________________________
Ort, Datum	Ort, Datum
______________________________	______________________________
Unterschrift des Prüfarztes	Unterschrift Probandin / Proband

18.8 Etikett der Prüfsubstanz

MIRACLE Studienpräparat **70 Kapseln zur Einnahme**

Verzehrsempfehlung:
Täglich morgens und abends 1 Kapsel mit einer Mahlzeit einnehmen.

Bei Raumtemperatur lagern! Unzugänglich für Kinder aufbewahren!

Studien-Registrierungsnummer (www.clinicaltrials.gov): NCT01360320

LOT-Nr. : xxxxxxxxxxxxxxxx
Mindestens haltbar bis :xx.x.201x

Kontaktadresse:
Koordinierungszentrum für Klinische Studien
der Medizinischen Fakultät der
Martin-Luther-Universität Halle-Wittenberg
06097 Halle (Saale)
Kostenlose Servicenummer: 0800/xxxxxxxx
http://www.grünteestudie.de

18.9 Fragebogen „Alternative Heilmethoden”

UKH Universitätsklinikum Halle (Saale)

gefördert durch Deutsche Krebshilfe HELFEN. FORSCHEN. INFORMIEREN.

ulm university universität uulm

Department für Innere Medizin
Universitätsklinik und Poliklinik
für Innere Medizin I
Ernst-Grube-Straße 40
06120 Halle (Saale)
Tel. 0345/5570
Direktor:
Prof. Dr. med. Th. Seufferlein

Institut für Naturheilkunde und
Klinische Pharmakologie der
Universität Ulm
Helmholtzstr. 20
89081 Ulm
Prof. Dr. med. J. Stingl

Fragebogen „Alternative Heilmethoden“ für Probanden

MIRACLE-Studie

Bevor die Befragung beginnt, benötigen wir von Ihnen noch einige Angaben für die spätere Auswertung:

➔ **Probanden-ID:**

➔ Mein letzter Schulabschluss:

☐ Hauptschule ☐ Realschule ☐ Abitur ☐ abgeschl. Studium

Meine Nationalität: ___________________

- Wir wollen Sie über ihre persönlichen Ansichten über Medikamente und alternative Heilmethoden befragen.
- Unten sehen Sie Meinungen anderer Menschen über Medikamente und alternative Heilmethoden.
- Bitte kreuzen Sie an, wie sehr Sie diesen Meinungen zustimmen!

Es gibt keine richtigen oder falschen Antworten. Wir sind an Ihrer persönlichen Sicht interessiert.

**Meinung zu Medikamenten und Nahrungsergänzungsmitteln**	stimme gar nicht zu	stimme nicht zu	weiß nicht	stimme zu	stimme voll zu
1. Ärzte verschreiben zu viele Medikamente.					
2. Menschen, die Medikamente schlucken, sollten bei der Einnahme ab und zu eine Pause einlegen.					
3. Medikamente helfen vielen Menschen besser zu leben.					
4. Die meisten Medikamente machen süchtig.					
5. Naturheilmittel sind sicherer als Medikamente.					
6. In den meisten Fällen überwiegt bei Medikamenten der Nutzen gegenüber den Risiken.					
7. In Zukunft wird man Medikamente entwickeln, die die meisten Krankheiten heilen können.					
8. Medikamente schaden mehr als dass sie nützen.					
	stimme gar nicht zu	stimme nicht zu	weiß nicht	stimme zu	stimme voll zu
9. Fast alle Medikamente sind Gift.					
10. Medikamente verlängern das Leben vieler Menschen.					
11. Ärzte vertrauen Medikamenten zu sehr.					
12. Wenn Ärzte mehr Zeit für ihre Patienten hätten, würden sie weniger Medikamente verschreiben.					
13. Mein Körper reagiert sehr sensibel auf Medikamente.					

14. Ich reagiere normalerweise stärker auf Medikamente als die meisten Menschen.					
15. Früher habe ich Medikamente schon mal schlecht vertragen.					
16. Schon sehr kleine Mengen an Medikamenten können meinen Körper umhauen.					
	stimme gar nicht zu	stimme nicht zu	weiß nicht	stimme zu	stimme voll zu
17. Ich würde meine Gene testen lassen, wenn ich dadurch wissen könnte, welches Medikament am besten zu mir passt.					
18. Pflanzliche Medikamente sind verträglicher als chemische hergestellte.					
19. Pflanzliche Medikamente wirken besser als chemisch hergestellte.					
20. Wenn möglich, würde ich ein pflanzliches Medikament bevorzugen.					
21. Ich interessiere mich für alternative Heilmethoden. (z.B. Homöopathie, Akkupuktur, traditionelle chinesische Medizin, Ayurveda)					
22. Ich nehme regelmäßig alternative Heilmethoden in Anspruch (z.B. Akkupunktur, homöopathische Medikamente, spezielle Kuren).					
23. Ich sehe in alternativen Heilmethoden eine sinnvolle Ergänzung der „klassischen Schulmedizin“.					
24. Ich nehme regelmäßig Nahrungsmittel oder Nahrungszusätze zur Prävention ein (z.B. probiotische Lebensmittel).					
25. Alternative Heilmethoden lohnen den finanziellen Mehraufwand					

Wir möchten uns bei Ihnen herzlich bedanken, dass Sie sich für diesen Fragebogen Zeit genommen haben.

Die MIRACLE-Studienleitung

18.10 ECOG-Performance Status [50]

Grade	Description
0	Fully active, able to carry on all pre-diseases performance without restriction. (Karnofsky 90-100)
1	Restricted in physically strenuous activity but ambulatory and able to carry out work of a light or sedentary nature (e.g., light housework, office work). (Karnofsky 70-80)
2	Ambulatory and capable of all self-care but unable to carry out any work activities. Up and about more than 50% of waking hours. (Karnofsky 50-60)
3	Capable of only limited self-care, confined to bed or chair more than 50% of waking hours. (Karnofsky 30-40)
4	Completely disabled. Cannot carry on any self-care. Totally confined to bed or chair. (Karnofsky 10-20)
5	Death

18.11 World Medical Association: Declaration of Helsinki [49]

Recommendations guiding physicians in biomedical research involving human subjects.

Adopted by the 18th World Medical Assembly Helsinki, Finland, June 1964 and amended by the 29th World Medical Assembly, Tokyo, Japan, October 1975, 35th World Medical Assembly Venice, Italy, October 1983, 41st World Medical Assembly Hong Kong, September 1989 and the 48th General Assembly Somerset West, Republic of South Africa, October 1996

INTRODUCTION

It is the mission of the physician to safeguard the health of the people. His or her knowledge and conscience are dedicated to the fulfillment of this mission.

The Declaration of Geneva of the World Medical Association binds the physician with the words, "The health of my patient will be my first consideration," and the International Code of Medical Ethics declares that, "A physician shall act only in the patient's interest when providing medical care which might have the effect of weakening the physical and mental condition of the patient."

The purpose of biomedical research involving human subjects must be to improve diagnostic, therapeutic and prophylactic procedures and the understanding of the etiology and pathogenesis of disease.

In current medical practice most diagnostic, therapeutic or prophylactic procedures involve hazards. This applies especially to biomedical research.

Medical progress is based on research, which ultimately must rest in part on experimentation involving human subjects.

In the field of biomedical research a fundamental distinction must be recognized between medical research in which the aim is essentially diagnostic or therapeutic for a patient, and medical research, the essential object of which is purely scientific and without implying direct diagnostic or therapeutic value to the person subjected to the research.

Special caution must be exercised in the conduct of research, which may affect the environment, and the welfare of animals used for research must be respected.

Because it is essential that the results of laboratory experiments be applied to human beings to further scientific knowledge and to help suffering humanity, the World Medical Association has prepared the following recommendations as a guide to every physician in biomedical research involving human subjects. They should be kept under review in the future. It must be stressed that

the standards as drafted are only a guide to physicians all over the world. Physicians are not relieved from criminal, civil and ethical responsibilities under the laws of their own countries.

BASIC PRINCIPLES

1. Biomedical research involving human subjects must conform to generally accepted scientific principles and should be based on adequately performed laboratory and animal experimentation and on a thorough knowledge of the scientific literature.
2. The design and performance of each experimental procedure involving human subjects should be clearly formulated in an experimental protocol which should be transmitted for consideration, comment and guidance to a specially appointed committee independent of the investigator and the sponsor provided that this independent committee is in conformity with the laws and regulations of the country in which the research experiment is performed.
3. Biomedical research involving human subjects should be conducted only by scientifically qualified persons and under the supervision of a clinically competent medical person. The responsibility for the human subject must always rest with a medically qualified person and never rest on the subject of research, even though the subject has given his or her consent.
4. Biomedical research involving human subjects cannot legitimately be carried out unless the importance of the objective is in proportion to the inherent risk to the subject.
5. Every biomedical research project involving human subjects should be preceded by careful assessment of predictable risks in comparison with foreseeable benefits to the subject or to others. Concern for the interests of the subject must always prevail over the interests of science and society.
6. The right of the research subject to safeguard his or her integrity must always be respected. Every precaution should be taken to respect the privacy of the subject and to minimize the impact of the study on the subject's physical and mental integrity and on the personality of the subject.
7. Physicians should abstain from engaging in research projects involving human subjects unless they are satisfied that the hazards involved are believed to be predictable. Physicians should cease any investigation if the hazards are found to outweigh the potential benefits.

8. In publication of the results of his or her research, the physician is obliged to preserve the accuracy of the results. Reports of experimentation not in accordance with the principles laid down in this Declaration should not be accepted for publication.
9. In any research on human beings, each potential subject must be adequately informed of the aims, methods, anticipated benefits and potential hazards of the study and the discomfort it may entail. He or she should be informed that he or she is at liberty to abstain from participation in the study and that he or she is free to withdraw his or her consent to participation at any time. The physician should then obtain the subject's freely given informed consent, preferably in writing.
10. When obtaining informed consent for the research project the physician should be particularly cautious if the subject is in a dependent relationship to him or her or may consent under duress. In that case the informed consent should be obtained by a physician who is not engaged in the investigation and who is completely independent of this official relationship.
11. In case of legal incompetence, informed consent should be obtained from the legal guardian in accordance with national legislation. Where physical or mental incapacity makes it impossible to obtain informed consent, or when the subject is a minor, permission from the responsible relative replaces that of the subject in accordance with national legislation. Whenever the minor child is in fact able to give a consent, the minor's consent must be obtained in addition to the consent of the minor's legal guardian.
12. The research protocol should always contain a statement of the ethical considerations involved and should indicate that the principles enunciated in the present Declaration are complied with.

MEDICAL RESEARCH COMBINED WITH PROFESSIONAL CARE (Clinical research)

1. In the treatment of the sick person, the physician must be free to use a new diagnostic and therapeutic measure, if in his or her judgment it offers hope of saving life, re-establishing health or alleviating suffering.
2. The potential benefits, hazards and discomfort of a new method should be weighed against the advantages of the best current diagnostic and therapeutic methods.
3. In any medical study, every patient - including those of a control group, if any - should be

assured of the best proven diagnostic and therapeutic method. This does not exclude the use of inert placebo in studies where no proven diagnostic or therapeutic method exists.

4. The refusal of the patient to participate in a study must never interfere with the physician-patient relationship.
5. If the physician considers it essential not to obtain informed consent, the specific reasons for this proposal should be stated in the experimental protocol for transmission to the independent committee (I, 2).
6. The Physician can combine medical research with professional care, the objective being the acquisition of new medical knowledge, only to the extent that medical research is justified by its potential diagnostic or therapeutic value for the patient.

NON-THERAPEUTIC BIOMEDICAL RESEARCH INVOLVING HUMAN SUBJECTS (Non-clinical biomedical research)

1. In the purely scientific application of medical research carried out on a human being, it is the duty of the physician to remain the protector of the life and health of that person on whom biomedical research is being carried out.
2. The subjects should be volunteers - either healthy persons or patients for whom the experimental design is not related to the patient's illness.
3. The investigator or the investigating team should discontinue the research if in his/her or their judgment it may, if continued, be harmful to the individual.
4. In research on man, the interest of science and society should never take precedence over considerations related to the well being of the subject.

18.12 Versicherungsbescheinigungen

CHUBB

CHUBB INSURANCE COMPANY OF EUROPE SE

Direktion für Deutschland
Grafenberger Allee 295 · 40237 Düsseldorf
Telefon (0 211)87 73-0 · Fax (0211) 87 73-333

Versicherungsbestätigung / Certificate

**Versicherungsscheinnummer / Policy Number**	**35959225**
**Versicherungsnehmer / Insured**	Martin-Luther-Universität Halle-Wittenberg<br>Ernst-Grube-Strasse 40<br>06120 Halle
**Versicherer / Insurer**	CHUBB INSURANCE COMPANY OF EUROPE SE<br>Direktion für Deutschland<br>Grafenberger Allee 295 · 40237 Düsseldorf
**Versicherungsperiode / Policy Period**	Vom / from 01.08.2011 bis / to 31.08.2016
**Betreff / Subject**	Mimimizing the Risk of Metachronous Adenomas of the Colorectum with Green tea Extract.-M.I.R.A.C.L.E Studie.
**Protokoll Nummer / Protocol Number**	NCT01360320
**Anzahl der Probanden / Number of patients**	2914
**Geltungsbereich / Territory**	Deutschland / Germany

Für den Versicherungsnehmer besteht im Rahmen der Allgemeinen Versicherungsbedingungen für eine nicht der Versicherungspflicht unterliegende klinische Prüfung (AVB-Prob//NV-Objekt) Versicherungsschutz für die durch Gesundheitsschädigung eingetretenen materiellen Schäden mit nachfolgend aufgeführten Versicherungssummen:

Within the scope defined by the terms and conditions of this insurance our company grants the insured coverage against third-party-claims on the basis of legal liability provisions under private law for tangible damages because of a bodily injury within the following limits:

für die einzelne versicherte Person / for each insured person	€	250.000,00
für alle Versicherungsfälle der oben genannten klinischen Prüfung insgesamt / for all claims arising out of the above mentioned clinical trial	€	5.000.000,00

Diese Versicherungsbestätigung beeinflusst in keinem Fall den in der Police festgelegten Versicherungsschutz. Sie dient ausschließlich deklaratorischen Zwecken und überträgt keine Rechte an den Besitzer dieser Bestätigung. / This certificate of insurance neither affirmatively nor negatively amends, extends, or alters the coverage afforded by any insurance described herein and is only declarative and confers no rights upon the certificate holder.

Der Inhalt der Police 35959225 ist in jedem Fall maßgeblich und verbindlich. Der Versicherungsschutz wird durch diese Bestätigung weder ergänzt, noch erweitertet oder geändert. / The policy 35959225 only is legally binding. This certificate does not amend, extend or alters the coverage.

CHUBB INSURANCE COMPANY OF EUROPE SE
Direktion für Deutschland

Hamburg, 15. Juni 2011

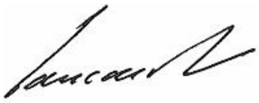

CHUBB INSURANCE COMPANY OF EUROPE SE
Eingetragener Sitz: 106 Fenchurch Street, London, EC3M 5NB, United Kingdom
Europäische Gesellschaft mit Sitz in England & Wales, eingetragen unter Company Number SE13.
Zugelassen durch die Financial Services Authority.
Niederlassung Deutschland: Grafenberger Allee 295, 40237 Düsseldorf, Bundesrepublik Deutschland
Hauptbevollmächtigter für die Bundesrepublik Deutschland: Thierry Daucourt, Amtsgericht Düsseldorf HRB 25138
The Royal Bank of Scotland N.V., Niederlassung Deutschland (BLZ 502 304 00) Kto.-Nr.:1470208024
IBAN: DE1 DE18502304001470208024, SWIFT: ABNADEFFFRA, USt-ID-Nr.: DE264642448
