# Supplementary material for: Population nutrikinetics of green tea extract
Source: PLoS One. 2018 Feb 21;13(2):e0193074. doi: 10.1371/journal.pone.0193074 (PMC5821365; doi:10.1371/journal.pone.0193074)
Supplement: S2 File — (PDF) [file pone.0193074.s003.pdf]

Nutrikinetik von Epigallokatechingallat (EGCG) in oraler Formulierung: Populationsvariabilität und Nutrigenomik

im Rahmen der

MIRACLE-Studie: Minimizing the Risk of Metachronous Adenomas of the Colorectum with Green Tea Extract

Studienplan

Version 2 vom 25.05.2011

STUDIENDURCHFÜHRUNG:
Institut für Naturheilkunde und Klinische Pharmakologie
Universität Ulm
Adresse Helmholtzstraße 20
D-89081 Ulm

Studienleitung:
Prof. Dr. Julia Stingl
Institut für Naturheilkunde und Klinische Pharmakologie
Telefon 0731- 500-65603
Fax 0731 – 500-65605

Es handelt sich um eine klinische Studie zu einem Nahrungsergänzungsmittel und nicht um eine Arzneimittelstudie nach §4, Absatz 23 des AMG.

Inhaltsverzeichnis

1. Zusammenfassung 4
2. Einleitung 5
2.1 Transport und Biotransformation von EGCG 5
2.2 Nutrikinetik von EGCG 5
2.3 Genetische Polymorphismen in COMT, OATP1A2 und 1B3 6
3. Studienziele 6
4. Studienpopulation 7
4.1. Einwilligungserklärung und Regularien 7
4.2. Einschlusskriterien 7
4.3. Ausschlusskriterien 7
4.4. Abbruchkriterien für den einzelnen Probanden 7
4.5. Abbruchkriterien für die Studie 8
4.6. Anzahl der Teilnehmer 8
5. Studiendauer 8
5.1. Studienbeginn und Studiendauer 8
5.2. Studiendauer für den einzelnen Probanden 8
6. Studiendurchführung 8
6.1. Voruntersuchung 8
6.2 Prüfsubstanz 8
6.3 Versuchsablauf 9
6.4 Ablaufschema 10
6.5 Studienbedingte Blutentnahmen 11
6.6. Geplante Laboruntersuchungen 11
7. Statistische Planung und Auswertung 13
7.1. Allgemeines 13
7.2 Primäre Zielgrösse und primäre Fragestellung 13
7.3 Sekundärvariablen 13
7.4 Fallzahlschätzung 13
7.5 Auswertungsmethodik: 14
8. Sicherheit - Risiken für Studienteilnehmer 14
8.1 Prüfsubstanz 14
8.2 Eingesetzte Menge 15
8.3 Mögliche Risiken und Nebenwirkungen 15
8.4 Blutabnahmen 15
8.5 Körperliche Aktivität während der Versuche 15
8.6 Nahrungs- und Flüssigkeitszufuhr 16
8.7 Probandenüberwachung 16
9. Unerwünschte Ereignisse 16
9.1 Definitionen 16
9.2. Handhabung und Berichtswesen von unerwünschten Ereignissen 17
9.3 Behandlung und Nachverfolgung von unerwünschten Ereignissen 18
10. Datenmanagement 18
10.1 Datenerfassung, -auswertung und -weitergabe 18
11. Verantwortlichkeiten und Unterschriften 19
Studienleitung, Durchführung, Auswertung: 19

Probenanalytik, Messungen von EGCG im Plasma, Pharmakokinetische Auswertung............19

1. Zusammenfassung

Titel: Nutrikinetik von Epigallokatechingallat (EGCG) in oraler Formulierung: Populationsvariabilität und Nutrigenomik

Leiterin der Studie
Prof. Dr. Julia Stingl
Institut für Naturheilkunde und Klinische Pharmakologie
Universität Ulm
Helmholtzstraße 20
D-89081 Ulm
Telefon: 0731 - 500-65603
Fax: 0731 - 500-65605

Zusammenfassung
Grüner Tee, aus den Blättern der Pflanze Camellia Sinensis hergestellt, gehört zu den ältesten und weltweit populärsten Getränken. Die Inhaltstoffe von Grünteeextrakt sind zu etwa 30%-70% Polyphenole, deren wichtigster Vertreter das (-)-Epigallocatechin-3-gallat (EGCG, hier auch einfach als Epigallokatechingallat bezeichnet) ist. Dieser Hauptinhaltsstoff von Grünem Tee (ca. 50% des Katechinanteils) zeigt neben positiven Wirkungen auf das kardiovaskuläre System auch präventive Wirkungen auf das Tumorwachstum bei Krebserkrankungen. Grüntee-Extrakt-Kapseln mit einem festen Gehalt an EGCG als Wirkstoff sind auf dem Markt frei als Nahrungsergänzungsmittel erhältlich und mittlerweile wird Grüntee-Extrakt auch vielen Nahrungsmitteln und Genussmitteln zugesetzt. In dieser Studie soll der Einfluss genetischer Varianten in Molekülen der Biotransformation und Arzneistofftransport auf die Kinetik von Epigallokatechingallat und anderen Grüntee-Katechinen nach einwöchiger Einnahme von Extrakt in Kapselform mit 300mg EGCG täglich bei gesunden Probanden untersucht werden.
Dazu soll der Konzentrationsverlauf im Plasma bei n=100 gesunden Probanden, die über 4 Tage eine Dosis von 300 mg EGCG in Kapselform zu je 2x150mg morgens und abends mit 12h Einnahmeabstand einnehmen untersucht werden. An Tag 5 wird nach einer kontrollierten Einnahme von einer Kapsel 150 mg EGCG die Blutkonzentrationen von EGCG im Plasma über 10 h gemessen. Ausgewertet werden soll die orale EGCG-Clearance, sowie Nutrikinetikdaten zu den weiteren Katechinen (gemessen anhand Dosis/AUC) nach Einnahme einer Kapsel mit 150 mg EGCG auf nüchternen Magen in Abhängigkeit von pharmakogenetischen Varianten in Transportergenen (z.B. OATP1A2 und OATP1B3) sowie Genen der Biotransformation von EGCG wie z.B. die Catecholamin-O-Methyltransferase COMT, Glutathiontransferasen und Sulfotransferasen.
Diese Untersuchung soll ein besseres Verständnis individueller Variabilität in der Kinetik von EGCG ermöglichen, was dann wiederum Auswirkungen auf individuelle Wirkung einer Einnahme von Grüntee-Extrakt haben kann.

Studienteilnehmer

N=100
Prüfdesign: offen, Nutrikinetikstudie
Geplante Probandenzahl 100
Studiendauer für den einzelnen Probanden Einnahme über 4 Tage je zweimal täglich 150 mg EGCG, anschließend Blutentnahmen in über einen Tag (10h)
Geplante Dauer der Studie: 18 Monate

2. Einleitung

2.1 Transport und Biotransformation von EGCG

Grünteekatechine werden als monomere Flavan-3-ole über den Dünndarm resorbiert. Katechine erreichen Maximalkonzentrationen im Plasma nach ca. 1-4 Stunden und sind bei einer Plasmahalbwertszeit von ca. 3 Stunden nach 24 Stunden zu mehr als 99% ($1/2^7$*100=0,78% noch im Körper) wieder ausgeschieden [1, 2].
Es besteht ein aktiver Transport von EGCG in Hepatozyten sowie in Darmepithelzellen über die transporter OATP1A2 und 1B3 [3]. Außerdem wird EGCG über einen Efflux-Transport über MRP-1 und -2 über das Darmepithel ins Lumen und aus den Leberzellen hinaustransportiert. P-Glykoprotein (MDR1) wird durch Grünteekatechine (EGCG und weitere) gehemmt, ohne jedoch die Katechine zu transportieren [4]. In der Leber werden Katechine vorwiegend über Phase II Enzyme metabolisiert (glucuronidiert, methyliert und sulfatiert). Methylierung über das genetisch polymorphe Enzym Catechol-O-Methyltransferase (COMT) wurde beschrieben, sowie auch eine Hemmung der COMT durch EGCG [5]. Die Elimination von EGCG erfolgt innerhalb der ersten 6 Stunden nach Einnahme vorwiegend biliär.

2.2 Nutrikinetik von EGCG

Epigallocatechin-3-Gallat wird nach oraler Einnahme (Kapsel) schnell absorbiert und erreicht maximale Konzentrationen im Blut nach etwa 1-2 Stunden. Nach Einmaldosis einer 400 mg EGCG Kapsel wurden maximale Plasmakonzentrationen von 112ng/ml und eine AUC von 623ng/ml*h gemessen, nach Einmaldosis von 800 mg EGCG wurden 438ng/ml und eine AUC von 2779 ng/ml*h gemessen [6]. Die Halbwertszeit im Plasma von EGCG liegt bei 2-4 h. Die Einnahme von 400 mg und 800 mg über 4 Wochen führte zu keiner meßbaren Akkumulation (die Predose Werte 24 h nach letzter Dosierung unter multipler Dosierung lagen unterhalb der Nachweisgrenze), da die Halbwertszeit zu kurz ist (siehe Abbildung1). Dennoch lag die AUC bei der 800 mg Dosis unter multipler Dosierung um 60% höher als bei Einzeldosierung, was möglicherweise an einer Hemmung des präsystemischen intestinalen Metabolismus liegt. Bei einer Aufteilung von 800 mg/d in mehrere Einzeldosen ist damit mehr EGCG im Blut nachweisbar als bei einer täglichen Einmaldosis [7-9]. EGCG liegt im Plasma vorwiegend ungebunden (92%) vor, wird vorwiegend über die Galle intestinal ausgeschieden und im Urin weder unkonjugiert noch in der glukuronidierten Form nachgewiesen [7-9].

In Abb. 3 ist der Zeitverlauf über 24 h nach Einnahme einer 800mg- bzw. 400mg-haltigen Kapsel Epigallocatechin-3-Gallat dargestellt. Die runden Markierungen zeigen den Verlauf nach einer

einmaligen Einnahme („before"), die dreieckigen Markierungen zeigen den Verlauf nach 4-wöchiger Einnahme („after").

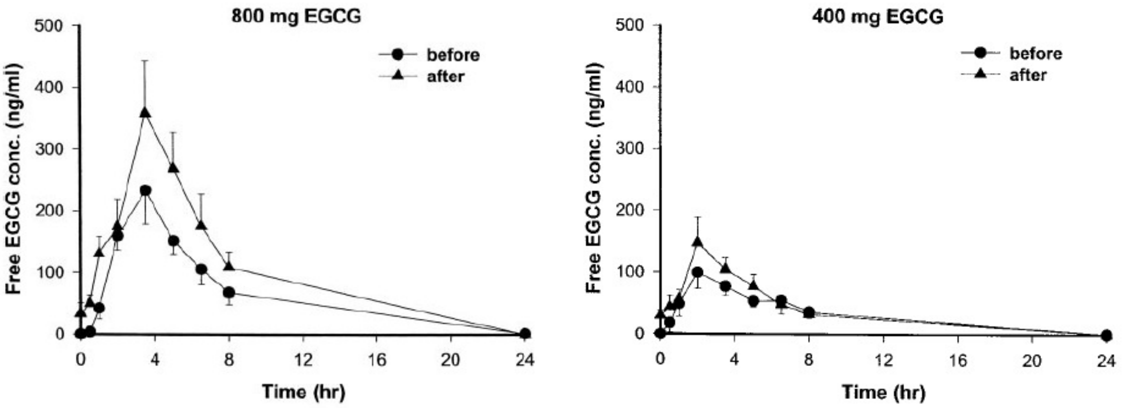

Abb. 1: Plasma-Konzentrationsverläufe von EGCG nach Einmaldosis von 800mg bzw. 400mg in Kapselform nach einmaliger (Kreise) bzw. nach vierwöchiger (Dreiecke) Einnahme. Abb. aus [7].

2.3 Genetische Polymorphismen in COMT, OATP1A2 und 1B3

Sowohl die am intestinalen und hepatischen Influx-Transport als auch die am (überwiegend hepatischen) Efflux-Transport von EGCG als auch an der Biotransformation beteiligten Transporter und Enzyme unterliegen genetischer Variabilität. Die Catecholamin-O-transferase ist ein in Leber, Niere und Gehirn vorkommendes Enzym, welches für die Methylierung von Neurotransmittern und Fremdstoffen von Bedeutung ist. EGCG wird ebenfalls über dieses Enzym methyliert, was prinzipiell interessanteWirkungen auf den Katecholaminstoffwechsel (wohl aber nur ausserhalb des Gehirns) erklären könnte. Es ist ein genetischer Polymorphismus beschrieben, welcher durch einen Valin zu Methionin Aminosäureaustausch im Enzym definiert ist und der zu einer trimodalen Verteilung der Enzymaktivität mit hoher Aktivität bei Trägern der Val/Val-Form, mittlerer bei Trägern von Val/Met sowie einer niedrigen Aktivität bei Trägern des Met/Met Genotyps führt [10].
Für die Transporter OATP1A2 und 1B3 sind eine Vielzahl z.T. auch funktionell bedeutsamer genetischer Varianten beschrieben, die bisher jedoch in ihrer Auswirkung auf den Transport von EGCG nicht untersucht sind [11, 12].

3. Studienziele

In dieser Studie soll die Kinetik nach oraler Mehrfachgabe von 300mg EGCG täglich nach 4-tägiger Einnahme in Abhängigkeit von pharmakogenetischen Varianten untersucht werden.

Prüfziel ist es, die orale Clarance (Dosis/AUC), sowie weitere Parameter der Pharmakokinetik wie Cmax und Tmax, sowie T1/2 von EGCG bei Trägern unterschiedlicher Genotyp-Varianten zu vergleichen.

4. Studienpopulation

4.1. Einwilligungserklärung und Regularien

Unbedingte Voraussetzung für die Teilnahme eines Probanden an der klinischen Studie ist seine schriftliche Einwilligung, nachdem er mündlich und schriftlich über Wesen, Bedeutung und Tragweite der klinischen Studie informiert wurde. Die Studie wird in Übereinstimmung mit der Deklaration von Helsinki (in der Revision von Edinburgh, Oktober 2000), den Prinzipien von Good Clinical Practice (GCP), der International Conference of Harmonisation (ICH) und den nationalen Verordnungen und Richtlinien durchgeführt. Die Versuchspersonen werden über alle Aspekte der Studie mit einem Informationsblatt aufgeklärt. Die erfolgte Probandeninformation wird zusätzlich zu der Einwilligungserklärung des Probanden durch den verantwortlichen Prüfarzt dokumentiert.

4.2. Einschlusskriterien

- Nach Voruntersuchung gesunde männliche oder weibliche Probanden
- Deutschebzw. nordeuropäische Abstammung (um eine genetisch einheitliche Population zu bekommen, keine rassistischen Intention)
- Vorliegen der schriftlichen Einverständniserklärung
- Mindestalter 18 Jahre
- BMI über 18 und unter 30

4.3. Ausschlusskriterien

- Bei Frauen Schwangerschaft oder Stillperiode
- schwere akute oder chronische Erkrankungen
- jede bekannte akute Infektionserkrankung (Studienbeginn kann dann ja verschoben werden)
- Jegliche Notwendigkeit einer regelmäßigen Medikamenteneinnahme außer orale Kontrazeptiva
- Notwendigkeit einer Medikamenteneinnahme während der Medikationsperiode und speziell am Studientag (z.B. Schmerzmittel oder Antibiotika).

4.4. Abbruchkriterien für den einzelnen Probanden

- Rücknahme der Einverständniserklärung.
- Auftreten eines Vorkommnisses oder unerwünschten Ereignisses, das nach Meinung des Prüfarztes ein Weiterführen der Studie nicht sinnvoll erscheinen lässt.

4.5. Abbruchkriterien für die Studie

Abbruchkriterien für die gesamte Studie werden nicht festgelegt. Bei Milliardenfacher Aufnahme der Prüfsubstanz in Form von grünem oder schwarzen Tee sind schwerwiegende Nebenwirkungen, die einen Abbruch der Studie erforderlich machen könnten, sehr unwahrscheinlich, aber in Hinblick auf die Herstellung der Prüfkapseln nicht vollkommen ausgeschlossen. Selbstverständlich wird bei jeder schwerwiegenden Nebenwirkung, die in Kausalzusammenhang mit dem Prüfpräparat stehen könnte, die Frage eines Abbruches der Studie erörtert.

4.6. Anzahl der Teilnehmer

100 Teilnehmer, die per Protokoll an der Studie komplett teilnehmen und auswertbar sind.

.

5. Studiendauer

5.1. Studienbeginn und Studiendauer

Der Beginn der Studie ist für September 2011 vorgesehen. Pro Proband wird ein Tag auf Probandenstation benötigt. Zum Einschluss von 100 Probanden werden voraussichtlich ca. 18 Monate benötigt.

5.2. Studiendauer für den einzelnen Probanden

Die Versuchsdauer für den einzelnen Probanden beläuft sich 4 Tage Einnahme (zuhause) sowie ein Tag Anwesenheit auf Probandenstation.

6. Studiendurchführung

6.1. Voruntersuchung

Die Interessenten werden mündlich und schriftlich über Ziel, Ablauf, Risiken, Art und Dauer der Studie informiert. Anschließend wird bei schriftlich erklärtem Einverständnis, an der Studie teilnehmen zu wollen, eine Blutentnahme zur Genotypisierung vorgenommen. Hierzu werden einmalig 9ml EDTA-Blut über eine Vene entnommen.
Wenn der Proband Träger eines der gesuchten Genotypen ist, wird ein Termin zur Voruntersuchung vereinbart, in welcher eine detaillierte Anamnese erhoben und eine körperliche Untersuchung vorgenommen wird. Es werden Routine-Laborparameter untersucht, die über die körperliche Untersuchung hinaus Auskunft über den Gesundheitszustand der Probanden geben. Es sollen folgende Parameter bestimmt werden: Blutbild, Kreatinin, Bilirubin, GOT, GPT, γGT.
Außerdem wird bei gebärfähigen Frauen ein Schwangerschaftstest durchgeführt. Nach Vorliegen aller Untersuchungsergebnisse wird über den Einschluss der Probanden in die Studie entschieden.

6.2 Prüfsubstanz

Es wird ein kommerziell erwerblicher Grüntee-Extrakt mit EGCG-Anteil von 50% verwendet. Dieser ist mit den Füllstoffen Cellulose und Mg-Stearat in Gelatine Hartkapseln enthalten. Die Kapseln werden von der Firma Dr. Loges, Hamburg hergestellt. Die Grüntee-Extraktkapseln sind die gleichen, die in einer multizentrischen Studie zur Untersuchung der Wirkung von Grünteeextrakt auf die Entwicklung von KolonAdenomen getestet werden (siehe Studie Ethikantrag 68/09).

Eine Kapsel enthält 150mg EGCG, sowie ca. 300mg Gesamtkatechine.

Es wird darauf Wert gelegt, dass die komplette Medikation aus einer Charge ist. Die Substanz wird trocken (Luftfeuchtigkeit unter 60%) und lichtgeschützt aufbewahrt und bei Raumtemperatur unter 25°C gelagert.

Die Einnahme am Kinetiktag erfolgt unter Aufsicht mit Dokumentation des genauen Einnahmezeitpunktes. Einnahme mit 200 ml Leitungswasser ohne Kohlensäure.

6.3 Versuchsablauf

Einnahmetage 1-4:
Die Probanden erhalten jeweils über 4 Tage morgens und mit 12h Abstand abends eine Kapsel mit 150mg EGCG entsprechend 300 mg EGCG pro Tag. Sie sollen diese zum Frühstück bzw. Abendessen mit einem Glas Leitungswasser einnehmen.

Studientage:
Am ersten Studientag müssen die Probanden nüchtern morgens auf der Probandenstation erscheinen. Nach kurzer Anamnese bezüglich des Gesundheitszustandes seit der Voruntersuchung wird ein venöser Zugang gelegt und eine Blutentnahme für den Basalwert der Kinetik durchgeführt.
Die Probanden erhalten nun nüchtern eine einmalige Gabe von 150 mg EGCG mit einem Glas Leitungswasser.
Anschließend werden über den Zeitraum von 10h einige Plasmaproben für die Bestimmung der Konzentrationen von EGCG entnommen. Die Probanden erhalten nach 4h und 10h je eine Mahlzeit sowie einen Snack. Sie sollen während der Zeit auf Probandenstation keine weiteren Mahlzeiten zu sich nehmen.

6.4 Ablaufschema

Tag	8.00	8:30	9.00	10:00	11:00	12:00	13:00	14:00	16:00	18:00	20:00
-x	**Voruntersuchung, BE: RNA, DNA, EDTA-Plasma für Analyse von Proteinen**										
-4	**1Kps. à 150mg EGCG**	← →<br>Die Einnahmen erfolgen zu Hause, wobei die Compliance dadurch erhöht werden soll, dass die ProbandInnen jeden Tag die Einnahmen in ein Probandentagebuch eintragen sollen, in das sie auch etwaige Nebenwirkungen eintragen sollen									**1Kps. à 150mg EGCG**
-3	**1Kps. à 150mg EGCG**										**1Kps. à 150mg EGCG**
-2	**1Kps. à 150mg EGCG**										**1Kps. à 150mg EGCG**
-1	**1Kps. à 150mg EGCG**										**1Kps. à 150mg EGCG**
1	**BE-0 (Predose): Einnahme 1 Kps. 150mg EGCG**	**BE-1: 10ml**	**BE-2: 10ml, UAW**	**BE-3: 40ml BE: RNA DNA, Plasma, CRP**	**BE-4: 10ml, UAW**	**BE-5: 10ml, UAW, Mittag-essen**	**BZ, BE-6: 10ml, UAW**	**BE-7: 10ml, UAW**	**BE-8: 10ml, UAW**	**BE-9: 10ml, UAW Abend-essen**	
Nahrung	Nüchtern ab 22:00 Vortrag	**	**	Kleiner Snack **nach** BE-3			Mittagessen **nach** BE-6			Abendessen **nach** BE-9	
	HF RR		HF RR	HF RR	HF RR	HF RR	HF RR	HF RR	HF RR	HF RR	

BE: Blutentnahme für Plasmakonz.-bestimmung UAW: Nebenwirkungsabfrage; grau schattiert: Anwesenheit auf Probandenstation, HF Herzfrequenz palpatorisch, RR Blutdruckmessung
****Sofern ProbandInnen in dieser Zeit unter Hypoglykämie leiden, können diese 10 g Traubenzuckerstücke oder ein zuverlässig nicht EGCG-haltiges Erfrischungsgetränk trinken (eher keine Fruchtsäfte).**

6.5 Studienbedingte Blutentnahmen

Bei der Voruntersuchung, zu denen die Probanden nüchtern (zumindest 3 h vorher nichts gegessen habend) kommen sollen, wird eine Blutentnahme für die Untersuchung von Routinelaborparametern entnommen: Blutbild, Kreatinin, Glukose, CRP, Bilirubin, GOT, GPT, γGT. Außerdem wird 10 ml Blut für Genotypisierung und für die Gewinnung von RNA und Proteinen aus Vollblut 30 ml Blut entnommen. Insgesamt würde bei der Voruntersuchung ein Volumen von 60 ml Blut abgenommen.

Am Studientag würde über 10 h Blut zur Bestimmung der EGCG Kinetik sowie 2 h nach Einnahme nochmals 30 ml Blut für RNA und Proteine und Entzündungsparameter (CRP). Für die EGCG-Pharmakokinetik ist folgendes Abnahmeschema vorgesehen: BE (jeweils 10 ml, EDTA-Monovette) zum Zeitpunkt vor Verabreichung (predose) sowie 30 min, 1, 2, 3, 4, 5, 6, 8, 10h nach Verabreichung (Siehe Ablaufschema). Sofern die Zeiten aus organisatorischen Gründen nicht mit einer Genauigkeit von +/- 5 Minuten (bei 30 min Abnahme +/- 2 Minuten) eingehalten werden könne, wird im CRF die wirkliche Entnahmezeit eingetragen. Letztere würde dann für die pharmakokinetischen Berechnungen berücksichtigt werden.

Insgesamt wäre das Blutvolumen, welches am Studientag entnommen würde 120ml.

6.6. Geplante Laboruntersuchungen

6.6.1 Genotypisierung

Die Blutproben zur Genotypisierung werden spätestens 24 h nach Abnahme bei -20°C eingefroren. Zur Bestimmung werden sie bei Raumtemperatur langsam aufgetaut. Die DNA-Extraktion erfolgt mittels standardisierter Verfahren (Qiagen-Kit zur DNA-Extraktion aus Vollblut). Es werden validierte Methoden zur Genotypisierung eingesetzt (DNA-Sequenzierung, Taqman, PCR-RFLP).

6.6.2 Genomweite Analysen

Die Studie ist primär nicht auf die Analyse mittels genomweiter DNA-Analysemethoden ausgerichtet. Es sind aber bestimmte Ergebnisse denkbar (z.B. sehr deutlich abweichende Nutrikinetik bei 6 der 100 Versuchsteilnehmer), die eine derartige genomweite Analyse aussichtsreich machen könnten. Insofern sollen die Probanden um ihr pinzipielles Einverständnis in genomweite Analysen gebeten werden. Diese Analysen würden sich auf das Ziel beschränken, unterschiede in der Nutrikinetik oder in den Wirkungen von EGCG aufzuklären (eng definierte Zweckbestimmung).

6.6.3 Kanditatengenanalysen

Es sollen Gene, für die ein Einfluß auf die Nutrikinetik oder die Wirkungen von EGCG nach existierender Literatur wahrscheinlich oder zumindest biologisch sehr plausibel ist, auf Genvarianten hin untersucht werden. Im ersten Schritt würden wir bekanntermassen funktionelle Varianten sowie Marker-Genvarianten für häufige Allele bzw. Haplotypen in diesen Genen untersuchen. Die Gene sind diejenigen, die für die Katechol-O-Methyltransferase (COMT), für Transportproteine wie OATP1A2 und -1B3 und weitere für EGCG relevante Transportproteine

kodieren. Ferner Gene, die für Glukuronyltransferasen kodieren und soweit wir tatsächlich signifikante Wirkungen von EGCG auf oxidativen Stress und Entzündung oder Kreislaufparamter sehen können, auch Gene, die damit in Zusammenhang stehen können.

Um in den pharmakogenetisch bislang noch weniger gut charakterisierten Genen wie OATP1B3neue Polymorphismen identifizieren zu können, erfolgt eine systematische Sequenzierung der kodierenden und angrenzenden nichtkodierenden Genbereiche einschließlich der 5'- und 3'-Region bei Untergruppen der Probanden, die durch Besonderheiten in der Kinetik oder in Wirkungen von EGCG auffällig sind. Sollten Mutationen detektiert werden, werden spezifische Genotypisierungsassays etabliert.

6.6.4 RNA-Isolierung und Bestimmung der Genexpression

Die Gesamt-RNA wird aus den Blutproben mittels Standardmethoden (Ficoll Zellseparation, Qiagen RNeasy Mini Kit, Pax-Gen) isoliert. Es ist geplant, in den Blutzellen zu untersuchen, ob sich ausgewählte Gene für Transportproteine, für Metabolismus, für Zellwachstum und für Bildung oder Detoxifikation reaktiver Sauerstoffverbindungen in ihrer Expression durch die 4tägige Behandlung mit EGCG verändern.
Im Falle von Einzelgenen erfolgt die Quantifizierung mittels Realtime-RT-PCR (Sybr Green, TaqMan). Auch hier soll wie bei den genetischen Analysen prinzipiell die Möglichkeit durch Information und Zustimmung der Probanden eröffnet werden, genomweite Expressionsanalysen durchzuführen. In diesem Falle würden die üblichen Affymetrix Expressions-Microarrays verwendet.
Neben der RNA sollen Genexpressionsunterschiede auch anhand Änderungen der Proteinmenge semiquantitativ im Western-Blot untersucht werden hierfür wird aus den isolierten Lymphozyten die Proteinfraktion extrahiert und tiefgefroren (-80°C) aufbewahrt.

6.6.5. Bioanalytik

Für die Bestimmung von EGCG im Blut werden 10 ml EDTA-Proben gewonnen, die vor Licht geschützt aufbewahrt werden und innerhalb von 30min nach Abnahme 10 min lang mit 2000 g Beschleunigung bei Raumtemperatur zentrifugiert werden.
Der Überstand (Plasma) wird gleichmäßig in Portionen zu je genau 500 µL Plasma aufgeteilt und jeweils mit 500 µL Stabilisierungspuffer gemischt werden. Der Stabilisierungspuffer besteht aus EDTA und Ascorbinsäure (Herstellung: 100 mg Ethylenediamin-Tetraessigäure (EDTA) Dinatriumsalz und 10 g Ascorbinsäure werden in 1000 mL Natriumazetatpuffer (35 mM: 2.38 g Na acetate trihydrate and 1.00 mL acetic acid dissolved in water and adjusted to 1000 mL) gelöst]. Die stabilisierten Plasmaproben werden dann bei -80°C gefroren aufbewahrt bis zur Versendung auf Trockeneis.

Die Arzneistoffanalytik erfolgt in der Abteilung Klinische Pharmakologie der Universität Göttingen, Direktor Prof. Dr. med. J. Brockmöller, mittels HPLC und elektrochemischer Detektion [13]. Dabei soll definitiv in Hinblick auf die Hauptzielgröße der Studie und in Hinblick auf die wichtigen Nebenzielgrößen die Plasmakonzentration von EGCG analysiert werden. Soweit möglich sollen auch Plasmakonzentrationen von methylierten Metaboliten (COMT), sowie von Glukuroniden oder Schwefelsäurekonjugaten des EGCG im Plasma oder im Urin

gemessen werden, um die Ursachen der vermuteten und sehr wahrscheinlichen Variabilität genauer charakterisieren zu können.

6.7. Aufwandsentschädigung und Nachuntersuchung

Die Nachuntersuchung umfasst dieselben Untersuchungen wie die Voruntersuchungen und wird zeitnah nach dem letzten Studientag durchgeführt. Die Probanden erhalten für die Studienteilnahme eine Aufwandsentschädigung von 80 €.

7. Statistische Planung und Auswertung

7.1. Allgemeines

Ziel der Studie ist es, den Einfluss genetischer Polymorphismen in COMT, OATP1A2 bzw. in OATP1B3 sowie weiteren an der Wirkung von EGCG beteiligten Molekülen auf die Kinetik von EGCG in vivo zu untersuchen. Es soll geprüft werden, ob sich die Plasmakonzentrationszeitverläufe (AUC, Cmax, orale Clearance) in Abhängigkeit der Genotypen unterscheidet.

7.2 Primäre Zielgrösse und primäre Fragestellung

Orale Clearance (Dosis/AUC) von 150mg EGCG bei Trägern von genetischen Polymorphismen im Vergleich zu Nichtträgern.

7.3 Sekundärvariablen

Maximals Blutkonzentration (C_{max}), Zeitpunkt der maximalen Blutkonzentration (T_{max}), Halbwertzeit ($T_{1/2}$), Unerwünschte Wirkungen, Genexpressionsänderungen, epigenetische Veränderungen in Blutzellen, Konzentrationsprofil der hier verwendeten Tabletten incl. Darstellung der Geschwindigkeit und Variabilität der Absorption, einer eventuellen Verteilungsphase (alpha-Halbwertzeit) und der terminalen Eliminationsphase (beta-Halbwertzeit).Plasmakonzentrationen der relevanten Grüntee-Katechine soweit diese bestimmbar sind.

7.4 Fallzahlschätzung

Eine Fallzahl von n=100 erlaubt es, genetische Varianten mit einer Allelfrequenz von 5% zumindest einmal homozygot zu finden mit einer Irrtumswahrscheinlichkeit von 5%.

Für den COMT Polymorphismus mit einer Häufigkeit von 20% Träger der Methionin-Variante (heterozygot plus homozygot), entsprechend einer Allelfrequenz von 11% wären folgende Einflüsse auf die Pharmakokinetik erkennbar: bei multipler Dosierung von 200mg EGCG wurde bei n=9 Probanden eine mittlere AUC von 1403 ng/ml h mit einer Standardabweichung von 632 beobachtet [8]. Bei einer angenommenen Zahl von n=20 Trägern entweder des Val/Met oder Met/Met Genotyps und n=80 Val/Val-Trägern wäre eine Erkennung von Unterschieden in der oralen Clearance von EGCG von 30% und mehr möglich mit einer Power von 80% und Signifikanzniveau 0.05 (Fallzahl gerechnet mit nQuery Advisor 4.0).

Über diese Darstellungen an einem Polymorphismus hinaus erlaubt die vorgesehene Fallzahl von 100 gut, die Verteilung von nutrikinetischen Parametern für EGCG in einer Stichprobe gesunder Personen zu erfassen und gibt uns für weitere Untersuchungen wichtige Hinweise, ob es vielleicht tatsächlich Untergruppen in der Bevölkerung gibt, die einerseits diese Substanz sehr schlecht resorbieren oder schnell metabolisieren, oder die andererseits diese Substanz sehr gut resorbieren oder nur sehr langsam ausscheiden.

7.5 Auswertungsmethodik:

Die Ergebnisse werden nichtparametrisch mit Hilfe des Jonckheere Terpstra Trend Tests basierend auf der Annahme eines Trends in der EGCG-Kinetik Wildtyp > heterozygote Träger > homozygote Träger ausgewertet.

Die beiden Extremgruppen Wildtyp und homozygote Träger werden außerdem mit Hilfe des parametrischen t-tests verglichen. Hinsichtlich der grundlegenden statistischen Probleme mit dem multiplen Testen ist die vorliegende Studien notwendigerweise exploratorischer Natur, eine bei monofaktorieller Testung signifikanter Zusammenhang wird als wichtiger Hinweis angesehen, auch wenn der Befund einer Adjustierung auf multiples Testen nicht standhält. Die vorliegende Studie wäre nach meiner Kenntnis die erste Studie, in der die genetische Variabilität des Tee-Inhaltsstoffes EGCG untersucht wird und hat insofern Pilotstudien-Charakter.

Die Berechnung der AUCs erfolgt zum einen in einer nichtkompartimentalen Auswertung, zum anderen mittels populationskinetischer Modellanalyse. Es wird eine experimentelle Datenanalyse mit Auswertung des Medians, Mittelwertes, Varianzparametern und eine graphische Darstellung durch Box-Plots durchgeführt.

Wertung von aus der Studie ausgeschiedenen Probanden:

Ein Proband, der die Studie vor Beendigung abbricht oder ausgeschlossen wird, wird als Dropout bezeichnet und geht nicht in die nutrikinetische Auswertung mit ein.

8. Sicherheit - Risiken für Studienteilnehmer

8.1 Prüfsubstanz

Für die Studie werden wird ein auf dem freien Markt erhältlicher Grüntee-Extrakt mit 50% Inhaltsstoff Epigallokatechingallat (EGCG) verabreicht. Es handelt sich um ein Nahrungsergänzungsmittel und um kein Medikament. EGCG ist der wirksame Hauptbestandteil von Grüntee-Extrakt. Es wird bei Genuss von grünem oder schwarzem Tee aufgenommen und ist auch als Nahrungsergänzungsmittel in Kapselform in Drogerien oder über das Internet frei erhältlich (z.B. Dr. Loges Prävent Kapseln oder BTC Vcaps).

8.2 Eingesetzte Menge

Für diese Studie wird eine Menge von 150 mg EGCG pro Kapsel und zweimal täglich eingesetzt (300 mg pro Tag und Proband). Dies entspricht in etwa der Menge, die in 3-5 Tassen grünem Tee täglich enthalten wäre.

8.3 Mögliche Risiken und Nebenwirkungen

Nebenwirkungen für Grüntee-Extrakte sind als selten für eine Dauereinnahme beschrieben, bei einer Einnahme von EGCG über 5 Tage sind im Allgemeinen keine Nebenwirkungen zu erwarten. In Einzelfällen wurde über das Auftreten von Leberenzymveränderungen im Zusammenhang mit der Einnahme von grünteehaltigen Nahrungsmitteln und Nahrungsergänzungsmitteln berichtet [14]. Insgesamt wurde aus insgesamt 216 Berichten über unerwünschte Ereignisse im zeitlichen Zusammenhang mit der Einnahme von grünteehaltigen Nahrungsmitteln, in 7 Fällen ein möglicher kausaler Zusammenhang zwischen einer Transaminasenerhöhung und der Einnahme von Grünteeextrakt hergestellt. Ursächlich scheint hier vor allem das als Hauptwirkstoff vorhandene EGCG verantwortlich zu sein, denn in Experimenten an isolierten Rattenleberzellen wurde eine 10-fach höhere Zelltoxizität von EGCG als von anderen Katechinen beobachtet [15].

Die in in dieser Untersuchung verwendete Dosis von 300mg EGCG entspricht der Aufnahme von ca. 4-7 Tassen grünem Tee täglich, was mit keinen bekannten Nebenwirkungen verbunden ist.

In einer Dosisfindungsstudie zu EGCG an 33 Patienten mit stabiler remittierter CLL wurden Dosierungen zwischen 400mg und 2000mg täglich über 6 Monate gegeben. In dieser Studie wurde bei 2 Patienten Toxizitäten Grad 3 (schwer) beobachtet, es handelte sich in einem Fall um Diarrhö, im anderen Fall um Bauchschmerz [16].

8.4 Blutabnahmen

Die Risiken von venösen Blutentnahmen bestehen in geringen bis mäßigen Schmerzen, Blutung oder Bildung eines Blutergusses. Außerordentlich seltene aber prinzipiell denkbare Komplikationen der Blutabnahme, über die die Probanden informiert werden, sind infektiöse Komplikationen an der Punktionsstelle, Nervenschädigungen, sowie schwerere Blutungen durch versehentliche arterielle Punktion. Wie bekannt, sind derartige Kompliationen aber außerordentlich selten und auch in unserer praktischen Erfahrung mit tausenden von Blutabnahmen sind derartigen schwere Komplikationen nie aufgetreten.

Prinzipiell können gegen alle verwendeten Materialien Allergien auftreten, relativ häufig werden Reizungen durch Pflaster beobachtet. Eine entsprechende Veranlagung wird anamnestisch erfragt und berücksichtigt.

8.5 Körperliche Aktivität während der Versuche

Während der Versuchszeit können sich die Probanden frei bewegen, sollen aber zu den Blutentnahmen liegen oder sitzen.

8.6 Nahrungs- und Flüssigkeitszufuhr

Die ProbandInnen sollen am Vortag vor dem Tag, an dem die nutrikinetische Analyse erfolge, keinen Tee trinken und insbesondere natürlich keinen grünen Tee. Eine Liste von Nahrungsstoffen, in denen eventuell Epigallocatechingallat oder ähnliche Verbindungen enthalten sein können, wird den Versuchsteilnehmern ausgehändigt.

Die ProbandInnen dürfen ab 22:00 vor dem Tag, an dem die nutrikinetische Studie stattfindet, bis 2 Stunden nach Einnahme der Prüfsubstanz keine Nahrung zu sich nehmen. Die Probanden erhalten dann ein Brötchen, sowie Mittagessen, Abendessen auf Station an den Versuchstagen, wobei in der Auswahl der Speisen darauf Wert gelegt wird, dass in diesen Speisen kein Epigallokatechingallat oder ähnliche Verbindungen enthalten sind.
Als Getränk erhalten sie Mineralwasser. Da die Nutrikinetik von EGCG bekanntermaßen von der gleichzeitigen Nahrungsaufnahme stark beeinflusst wird (über dreifach höhere Blutspiegel auf leeren Magen versus Einnahme mit Mahlzeit) [17], werden alle Probanden angehalten, nichts zusätzlich zu essen.
Die Flüssigkeitszufuhr pro Studientag soll nicht über 3 L liegen.

8.7 Probandenüberwachung

Zur Minimierung von Risiken bei der Studiendurchführung ist eine kontinuierliche Kontrolle durch geschultes medizinisches Personal gewährleistet. Ärztliches Personal ist während der gesamten Dauer eines Experimentes im Institut anwesend. Das gesamte an der Studie beteiligte Personal nimmt zu Studienbeginn an einer spezifischen studienbezogenen Schulung teil.

9. Unerwünschte Ereignisse

9.1 Definitionen

Ein **unerwünschtes Ereignis**_ist jedes nachteilige Vorkommnis, das einer betroffenen Person widerfährt, die an einer Studie teilnimmt, und das nicht notwendigerweise in ursächlichem Zusammenhang mit dieser Behandlung steht. Hierzu zählt jedes ungünstige gesundheitliche Ereignis bei einem Patienten bzw. Probanden, dem ein Arzneimittel oder in unserem Fall ein Nahrungsergänzungsmittel verabreicht wurde zwischen erster Einnahme des Prüfpräparates bis 24 Stunden nach letzter Einnahme.

Ein unerwünschtes Ereignis (UE) kann deshalb jedes ungünstige oder unbeabsichtigte Zeichen (einschließlich auffälliger Laborbefunde), Symptom oder jede Erkrankung sein, die in zeitlichem Zusammenhang mit der Anwendung einer Substanz oder einer medizinischen Intervention auftritt, unabhängig davon, ob ein Kausalzusammenhang angenommen wird.

Vorbestehende Erkrankungen, die sich im Verlauf einer Prüfung verschlimmern, sind ebenfalls als unerwünschte Ereignisse einzuordnen. Sie können zu schwerwiegenden unerwünschten Ereignissen werden, wenn sie eines der unten aufgeführten Kriterien erfüllen.

Ein **schwerwiegendes unerwünschtes Ereignis** ist jedes unerwünschte Ereignis oder jede Nebenwirkung, das unabhängig von einem ursächlichen Zusammenhang mit der Studienteilnahme zwischen erster Einnahme der Studienmedikation bis 24 Stunden nach letzter Einnahme eintritt und mindestens eines der folgenden Kriterien erfüllt

• letales (zum Tode führendes) Ereignis (***Hinweis***: der Tod ist die Folge, nicht das Ereignis selbst)
• lebensbedrohliches Ereignis (***Hinweis***: der Begriff "lebensbedrohlich" bezieht sich auf ein Ereignis, bei dem der Patient zum Zeitpunkt des Eintritts des Ereignisses in Lebensgefahr schwebte, nicht aber auf ein Ereignis, das zum Tode hätte führen können, wenn es schwerwiegender gewesen wäre)
• Notwendigkeit der außerplanmäßigen stationären Aufnahme oder der Verlängerung eines Krankenhausaufenthaltes eines Patienten
• zu bleibender oder erheblicher Behinderung oder Invalidität führendes Ereignis.
• zu einer angeborenen Anomalie oder zu einem Geburtsfehler führenden Ereignis
• medizinisch bedeutsames Ereignis oder jedes Ereignis, das eine Handlung erfordert, um eine der oben aufgeführten Folgeerscheinungen zu verhindern

Eine **Nebenwirkung** ist irgendein unerwünschtes Ereignis (letzteres oben definiert), für das ein ursächlicher Zusammenhang mit der Studie möglich, wahrscheinlich oder sicher ist.

Eine **schwerwiegende Nebenwirkung** ist ein schwerwiegendes unerwünschtes Ereignis (letzteres oben definiert), für das ein ursächlicher Zusammenhang mit der Studie möglich, wahrscheinlich oder sicher ist.

Eine **unerwartete schwerwiegende Nebenwirkung** ist ein schwerwiegendes unerwünschtes Ereignis, das nicht zu den typischen unterwünschten Ereignissen von Prüfsubstanzen oder Studieninterventionen gehört, die in der Probandeninformation aufgeführt sind.

9.2. Handhabung und Berichtswesen von unerwünschten Ereignissen

Alle nicht schwerwiegenden unerwünschten Ereignisse (UEs), die während der Studie auftreten, sind auf den vorgesehenen Seiten im CRF zu dokumentieren. Der Schweregrad bzw. die Intensität dieser unerwünschten Ereignisse ist anhand einer 4-Punkte Skala (leicht, mittelgradig, schwer, lebensbedrohlich) zu bewerten und entsprechend in den CRF einzutragen.

- Leicht: leichte Beschwerden, die die normalen täglichen Aktivitäten jedoch nicht beeinträchtigen
- Mittelgradig: die Beschwerden sind so ausgeprägt, dass die normalen täglichen Aktivitäten eingeschränkt oder beeinträchtigt werden
- Schwer: Unfähigkeit zur Arbeit oder zur Ausübung der normalen täglichen Aktivitäten
- Lebensbedrohlich: Stellt eine unmittelbare Lebensgefahr dar

Ferner ist der Zusammenhang von Behandlung und unerwünschtem Ereignis zu bewerten.

Die Ethik-Kommission wird über alle schwerwiegenden und alle unerwarteten schwerwiegenden Nebenwirkungen (wie in Absatz 9.1 definiert), die sich im Rahmen der Studie ereignen, so schnell wie möglich informiert.

9.3 Behandlung und Nachverfolgung von unerwünschten Ereignissen

Alle unerwünschten Ereignisse, insbesondere die, bei denen der Zusammenhang mit der Prüfmedikation vermutet wird, müssen so lange nachverfolgt werden, bis die Symptome entweder abgeklungen sind, der Basiszustand wieder erreicht ist oder sich der Zustand stabilisiert hat. Sollte eine eindeutige Erklärung für das unerwünschte Ereignis vorliegen, ist diese im CRF zu dokumentieren.

10. Datenmanagement

10.1 Datenerfassung, -auswertung und -weitergabe

Alle im Rahmen der Studie anfallenden Daten unterliegen dem Datenschutz. Personengebundene Daten werden mit Ausnahme von Geburtsdatum, Alter und Geschlecht in keiner Weise aus der Prüfstelle herausgegeben. Die Weitergabe von Prüfbögen und die Datenspeicherung zur Auswertung erfolgt nur mit pseudonymisierten Daten des Probanden. Eine Zuordnung der Probandencodes zu den persönlichen Daten des Probanden ist nur den Prüfern am jeweiligen Zentrum gestattet. Nach Abschluss der Studie werden die Studienunterlagen gemäß den Bestimmungen des Datenschutzes archiviert.

Alle Studiendaten werden in Prüfbogen (CRFs) erfasst. Folgende Prüfbögen werden durch das Prüfzentrum erstellt:

- Dokumentation der Probandeneinverständniserklärung
- Screening
- Ein- / Ausschlusskriterien
- Versuchsablauf
- Studienabschluss
- Dokumentation unerwünschter Ereignisse

Die DNA/RNA-Proben sowie Restblutproben werden mit dem Studiencode (EGCG_Kin) und der Probandennummer verschlüsselt und zeitlich unbegrenzt aufbewahrt, so lange eine ordnungsgemäße Lagerung möglich ist und so lange eine wissenschaftliche Nutzung zu den gegenüber den Versuchsteilnehmern dargestellten Versuchszielen möglich ist.
Der Grund für die lange Aufbewahrungsdauer liegt darin, dass mit dem vorliegenden Probenmaterial zahlreiche Targets (z. B. im Genom, Transkriptom, Proteom, Metabolom) untersucht werden sollen, die Analysen dafür aber in den meisten Fällen sehr aufwendig sind und sich deshalb über mehrere Jahre hinziehen können. Über die unbegrenzte Lagerung des Materials an der Universität Ulm und die Weitergabe der codierten Plasma- und Urinproben für die Konzentrationsanalysen an der Universität Göttingen wird der Proband bei der Aufklärung explizit hingewiesen und um seine Zustimmung gebeten. Für den Fall, dass eine vorzeitige Vernichtung des Probenmaterials von dem Probanden nach erfolgter Einwilligung später gewünscht wird, kann der Proband direkt mit den verantwortlichen Studienärzten Kontakt aufnehmen. Anhand der geführten Identifikationsliste wird dem Probanden seine Probe zugeordnet und anschließend vernichtet. Die Probanden werden im Informationsblatt explizit über diesen Sachverhalt hingewiesen. Die Verwendung der Probandenproben ist auf Untersuchungen zu Kinetik und Wirkung von EGCG begrenzt.

10.2. Pseudonymisierung der Daten und Wahrung der Schweigepflicht
Personen, denen ein Zugriff auf die Daten ermöglicht wird, werden über die Vertraulichkeit informiert und bestätigen diese Information über den Datenschutz und die Schweigepflicht mit ihre Unterschrift und unterliegen der ärztlichen Schweigepflicht. Eine Weitergabe der Daten ist ihnen untersagt.

11. Verantwortlichkeiten und Unterschriften

Studienleitung, Durchführung, Auswertung:

Prof. Dr. med. Julia Kirchheiner
Institut für Naturheilkunde und Klinische Pharmakologie
Universität Ulm
Helmholtzstr. 20
D-89081 Ulm
Tel: 0731-65603
Fax: 0731-65605
E-mail: julia.kirchheiner@uni-ulm.de

Pharmakogenetik/ Biomarker Screen:
Dr. med. Angela Seeringer
Sektion Klinische Pharmakologie
Institut für Naturheilkunde und Klinische Pharmakologie
Universität Ulm
Helmholtzstr. 20
89081 Ulm
Tel: 0731-65612
Fax: 0731-65605

Probenanalytik, Messungen von EGCG im Plasma, Pharmakokinetische Auswertung

Prof. Dr. med. Jürgen Brockmöller
Abteilung Klinische Pharmakologie der Universität Göttingen
Robert-Koch-Str. 40
D-37075 Göttingen, Germany
Telephone: +49 - 551 - 395770
Fax: +49 - 551 - 3912767
E-mail: jbrockm@gwdg.de

Förderung / Finanzierung:
Deutsche Krebshilfe

Zuständige Ethik-Kommission:

Ethik-Kommission der Medizinischen Fakultät der Universität Ulm

Prof. Dr. med. Uwe Brückner, Vorsitzender

Unterschrift Studienleiter:

Datum:<br><br><br>Prof. Dr. J. Stingl<br>(Name)

12. Literatur

1. Lee, M.J., et al. (2002). Pharmacokinetics of tea catechins after ingestion of green tea and (-)-epigallocatechin-3-gallate by humans: formation of different metabolites and individual variability. *Cancer Epidemiol Biomarkers Prev*. 11(10 Pt 1): p. 1025-32.
2. Yang, C.S., et al. (1998). Blood and urine levels of tea catechins after ingestion of different amounts of green tea by human volunteers. *Cancer Epidemiol Biomarkers Prev*. 7(4): p. 351-4.
3. Roth, M., B. Timmermann, and B. Hagenbuch (2011). Interactions of green tea catechins with organic anion transporting polypeptides. *Drug Metab Dispos*. epub ahead of print.
4. Jodoin, J., M. Demeule, and R. Beliveau (2002). Inhibition of the multidrug resistance P-glycoprotein activity by green tea polyphenols. *Biochim Biophys Acta*. 1542(1-3): p. 149-59.
5. Zhu, B.T., et al. (2000). O-Methylation of tea polyphenols catalyzed by human placental cytosolic catechol-O-methyltransferase. *Drug Metab Dispos*. 28(9): p. 1024-30.
6. Chow, H.H., et al. (2001). Phase I pharmacokinetic study of tea polyphenols following single-dose administration of epigallocatechin gallate and polyphenon E. *Cancer Epidemiol Biomarkers Prev*. 10(1): p. 53-8.
7. Chow, H.H., et al. (2003). Pharmacokinetics and safety of green tea polyphenols after multiple-dose administration of epigallocatechin gallate and polyphenon E in healthy individuals. *Clin Cancer Res*. 9(9): p. 3312-9.
8. Ullmann, U., et al. (2004). Plasma-kinetic characteristics of purified and isolated green tea catechin epigallocatechin gallate (EGCG) after 10 days repeated dosing in healthy volunteers. *Int J Vitam Nutr Res*. 74(4): p. 269-78.
9. Ullmann, U., et al. (2003). A single ascending dose study of epigallocatechin gallate in healthy volunteers. *J Int Med Res*. 31(2): p. 88-101.
10. Lachman, H.M. (2008). Does COMT val158met affect behavioral phenotypes: yes, no, maybe? *Neuropsychopharmacology*. 33(13): p. 3027-9.
11. Franke, R.M., L.A. Scherkenbach, and A. Sparreboom (2009). Pharmacogenetics of the organic anion transporting polypeptide 1A2. *Pharmacogenomics*. 10(3): p. 339-44.
12. Schwarz, U.I., et al. Identification of novel functional organic anion-transporting polypeptide 1B3 polymorphisms and assessment of substrate specificity. *Pharmacogenet Genomics*. 21(3): p. 103-14.
13. Lee, M.J., et al. (1995). Analysis of plasma and urinary tea polyphenols in human subjects. *Cancer Epidemiol Biomarkers Prev*. 4(4): p. 393-9.
14. Sarma, D.N., et al. (2008). Safety of green tea extracts : a systematic review by the US Pharmacopeia. *Drug Saf*. 31(6): p. 469-84.
15. Galati, G., et al. (2006). Cellular and in vivo hepatotoxicity caused by green tea phenolic acids and catechins. *Free Radic Biol Med*. 40(4): p. 570-80.
16. Shanafelt, T.D., et al. (2009). Phase I trial of daily oral Polyphenon E in patients with asymptomatic Rai stage 0 to II chronic lymphocytic leukemia. *J Clin Oncol*. 27(23): p. 3808-14.
17. Chow, H.H., et al. (2005). Effects of dosing condition on the oral bioavailability of green tea catechins after single-dose administration of Polyphenon E in healthy individuals. *Clin Cancer Res*. 11(12): p. 4627-33.
