# Supplementary material for: Population nutrikinetics of green tea extract
Source: PLoS One. 2018 Feb 21;13(2):e0193074. doi: 10.1371/journal.pone.0193074 (PMC5821365; doi:10.1371/journal.pone.0193074)
Supplement: S3 File — (PDF) [file pone.0193074.s004.pdf]

Nutrikinetics of Epigallocatechingallate (EGCG) in oral formulation: population variability and nutrigenomics

As part of

MIRACLE-Study : Minimizing the Risk of Metachronous Adenomas of the Colorectum with Green Tea Extract:

Study Protocol
Version 2 - 25.05.2011

Study Centre:
Institut für Naturheilkunde und Klinische Pharmakologie
Universität Ulm
Adresse Helmholtzstraße 20
D-89081 Ulm

Principle Investigator:
Prof. Dr. Julia Stingl
Institut für Naturheilkunde und Klinische Pharmakologie
Telefon 0731- 500-65603
Fax 0731 – 500-65605

This is a clinical trial to study a dietary supplement and not a drug study according to §4, Absatz 23 of AMG.

Table of content

1. Summary 3
2. Introduction 4
    2.1 Transport and Biotransformation of EGCG 4
    2.2 Nutrikinetics of EGCG 4
    2.3 Genetic polymorphisms in COMT, OATP1A2 and 1B3 5
3. Aim of the Study 5
4. Study population 6
    4.1. Informed consent and regulation 6
    4.2. Inclusion criteria 6
    4.3. Exclusion criteria 6
    4.4. Stop criteria for each participants 6
    4.5. Stop criteria for the study 6
    4.6. Number of participants 6
5. Duration of the study 7
    5.1. Start and duration of the study 7
    5.2. Duration for each participant 7
6. Execution of the study 7
    6.1. Preliminary check-up 7
    6.2. Study drug 7
    6.3. Study Procedure 8
    6.4 Schema: 9
    6.5 Blood sampling during study 10
    6.6. Experimental Procedure 10
7. Statistical analysis 11
    7.1. General 11
    7.2 Primary outcome 12
    7.3 Secondary outcome 12
    7.4 Sample size estimation 12
    7.5 Statistical methods: 12
8. Safety – Risk for Participants 13
    8.1 Study drug 13
    8.2 Amount of drug 13
    8.3 Possible risks and adverse events 13
    8.4 Blood sampling 13
    8.5 Physical activity during study day 14
    8.6 Eating and drinking during study. 14
    8.7 Monitoring of the participants 14
9. Adverse Events 14
    9.1 Definitions 14
    9.2. Handling and documentation of adverse events 15
    9.3 Handling and follow-up of adverse events. 16
10. Data management 16
    10.1 Data collection, data analysis and data transfer 16
11. Responsibilities and Signature 17

1. Summary

Title: Nutrikinetics of Epigallocatechingallate (EGCG) in oral formulation: population variability and nutrigenomics

Principle Investigator:
Prof. Dr. Julia Stingl
Institut für Naturheilkunde und Klinische Pharmakologie
University Ulm
Helmholtzstraße 20
D-89081 Ulm
Telefon: 0731 - 500-65603
Fax: 0731 - 500-65605

Summary
Green tea from the leaves of Camellia Sinensis is one of the oldest and most popular beverages worldwide. Green tea extract contains around 30-70% polyphenols of which (-)-Epigallocatechin-3-gallat (EGCG) is the most important compound. This major compound of green tea (approximately 50% of total catechins) shows positive effects on the cardiovascular system but also a preventive effect on tumor growth during cancer development. Green tea extract capsules normalized to a definite amount of EGCG are available on the market as dietary supplements, but green tea extract is also added to other food products and beverages. In this study we want to study the influence of genetic variants in genes of the biotransformation and drug transport on the kinetics of EGCG and other green tea catechins after one week of daily oral intake of green tea extract capsules with 300mg EGCG on healthy volunteers. Plasma-concentration-time profiles of n=100 healthy participants will be studied after 4 day twice daily (morning and evening) intake of 2x150mg (=300mg EGCG) with 12h gap in-between. On day 5 the blood concentrations of EGCG will be measured for 10h after the controlled intake of one capsule with 150mg EGCG. The oral EGCG clearance as well as nutrikinetic data of further catechins (measured as Dosage/AUC) after intake of 150mg EGCG under fasting conditions should be analyzed in relation to pharmacogenetics variants in transporter genes (e.g. OATP1A2 and OATP1B3) and in genes of the biotransformation of EGCG for example Catecholamin-O-Methyltransferase (COMT), Glutathiontransferase and Sulfotransferase.
This study should allow a better understanding of individual variability in the kinetic of EGCG, which has an influence on the individual efficacy of green tea intake.

Participants

N=100
Study Design: open, Nutrikinetic study
Planned number of participants 100
Study duration for each participant Intake of two times 150mg EGCG over 4 days, followed by blood sampling over 1d (10h)
Study duration: 18 month

2. Introduction

2.1 Transport and Biotransformation of EGCG

Green tea catechins are resorbed in the small intestine as monomer Flavan-3-ole. Catechins reach their maximal plasma concentration after 1-4 hours and to more than 99% they are excreted 24h with plasma half live of 3h. [1, 2]. EGCG is actively transported in hepatocytes and intestinal epithelium cells over the transporter OATP1A2 and 1B3 [3]. Additionally EGCG is transported over an efflux transport with MRP-1 and MRP-2 into the intestinal lumen or from liver cells. P-glycoprotein (MDR1) is inhibited by green tea catechins (EGCG and others) but is not transporting catechins [4]. In the liver catechins are mainly metabolized by phase II enzymes (glucuronidated, methylated and sulfated). Methylation was described over the genetic polymorph enzyme Catechol-O-Methyltransferase (COMT) and COMT is also inhibited by EGCG [5]. EGCG is mainly eliminated biliary within the first 6h after intake.

2.2 Nutrikinetics of EGCG

EGCG is quickly absorbed after oral administration (capsule) and reaches the maximal blood concentrations after 1-2 hours. After single dose administration of 400mg EGCG capsules maximal plasma concentration of 112ng/ml and an AUC of 623ng/ml*h was measured, after a single dose of 800 mg EGCG 438ng/ml and an AUC of 2779 ng/ml*h was determined [6]. The half-life time of EGCG in plasma is 2-4 h. The intake of 400 mg and 800 mg over 4 weeks does not lead to any accumulation (under multiple dosing values measured 24 h after the last administration were below limit of detection), because the half-life time is to short (see figure1).
Nevertheless AUC of a 800 mg doses was 60% under multiple dosing than single dosing. This might be due to an inhibition of presystemic intestinal metabolism. Splitting 800mg/day into several single doses leads therefore to higher blood levels than a single dose [7-9]. EGCG can be found mostly unbound in the plasma (92%), is biliary eliminated and can be neither detected in its conjugated nor glucuronidated form in urine [7-9]. In figure 3 the time-plasma concentration profiles after administration of 800mg and 400mg EGCG capsules are presented. Dots represent the profile after single dose (“before”), triangles represent the profile after 4 weeks of intake (“after”).

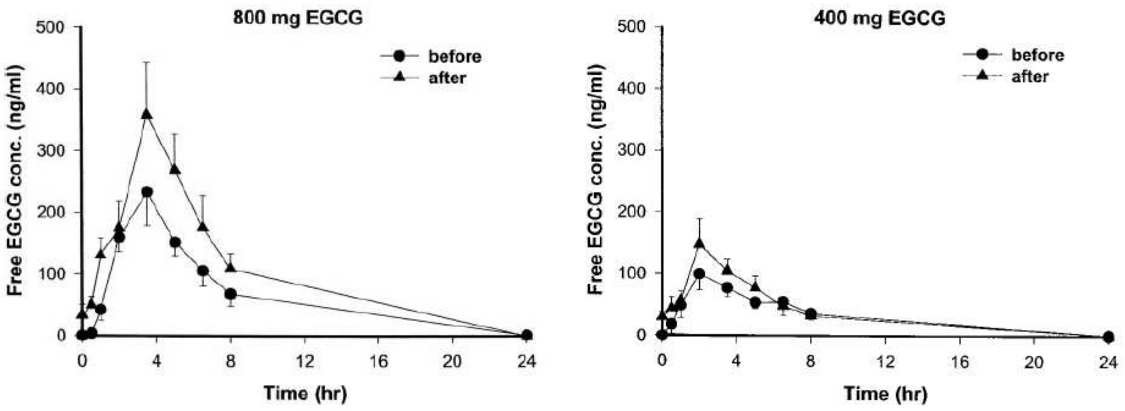

Fig. 1: Plasma concentration profiles after administration of 800mg and 400mg EGCG capsules after single doses (dots) and 4 week soft intake (triangles) Fig. from ref. [7].

2.3 Genetic polymorphisms in COMT, OATP1A2 and 1B3

Intestinal and hepatic influx transporter, as well as the (mainly hepatic) efflux transporter of EGCG and transporter and enzymes involved in biotransformation show genetic variability. Catecholamin-O-transferase (COMT) is an enzyme expressed in liver, kidney and brain and is involved in the methylation of neurotransmitters and xenobiotics. EGCG is methylated via COMT. This could in principle explain the interesting effect on the catecholamine metabolism (most probably outside the brain). A genetic polymorphism is described resulting in an amino acid exchange from valine to methionine. This change results in a trimodal distribution of enzyme activity with high activity in carriers with Val/Val form, medium activity in carriers with Val/Met and low activity in carriers of the Met/Met genotype. [10].
Many genetic variants are described in OATP1A2 and 1B3 transporter, but is not yet studied to which extent these variants effect the transport of EGCG.

3. Aim of the Study

In this study we will investigate the pharmacokinetic after multiple oral dosing of 300mg EGCG daily after 4-days of intake in relation to pharmacogenetics variants. It is the aim to compare the oral clearance (Dosage/AUC) as well as further pharmacokinetic parameters of EGCG as Cmax, Tmax, T1/2 in carriers of different genotype variants.

4. Study population

4.1. Informed consent and regulation

Essential requirement for the participation of a test person in this trial is a written consent after he was written and orally informed about the study. This study is conducted in agreement with the Declaration of Helsinki (in revision from Edinburgh, October 2000), the principle of Good Clinical Practice (GCP), of the International Conference of Harmonization (ICH) and other national regulations and guidelines. The participants are informed on all aspects of the study in an information leaflet. The information of the participant is documented by the clinical study investigator together with the written consent of the participant.

4.2. Inclusion criteria

- Healthy male and female participants after physical check up
- German or northern European origin (to obtain a genetic homogenous population, no intention to racism)
- Written informed consent
- Age > 18 years
- BMI between 18 and 30

4.3. Exclusion criteria

- For female pregnancy and breast feeding
- Acute and chronic disease
- Each acute infectious disease (start of the participation can be delayed)
- Regular drug intake with exception to oral contraceptives
- Need to take drugs during study period and especially at study day (e.g. analgesics or antibiotics).

4.4. Stop criteria for each participants

- Withdrawal of consent.
- Occurrence of an occasion or an adverse event which in opinion to the clinical study investigator makes it useless to continue the study

4.5. Stop criteria for the study

Stop criteria for the study are not defined. The occurrence of severe adverse events which relative unlikely due to millions using green and black tea every day, but it cannot be completely excluded for the extract capsules used in this trial. Certainly, we will discuss the stopping of the study whenever a severe adverse event happens which is in clear causality with the test drug.

4.6. Number of participants

100 participants, which completed the protocol and can be analyzed.

.

5. Duration of the study

5.1. Start and duration of the study

The start of the study is planned for September 2011. Each participant spends one day on the research ward. For 100 participants around 18 months are planned.
.

5.2. Duration for each participant

The study duration for each participant is four days intake of capsules at home plus one day on the research ward (study day).

6. Execution of the study

6.1. Preliminary check-up

Interested persons will be written and orally informed about the aim, procedure, risks and duration of the study. After written consent a blood sample for genotyping is taken. For this reason 9 ml of venous EDTA blood is taken. If the participant is a carrier of a wanted genotype an appointment for a preliminary check-up is arranged. At this preliminary check-up a physical check-up is made and laboratory parameters are analyzed from blood giving information on the health status of the participants. Following parameters will be determined: Blood cell count, creatinine, bilirubin, GOT, GPT, γGT and a pregnancy test for women. According to the results a decision on inclusion in the study is made.

6.2. Study drug

A commercially available green tea extract with an EGCG content of 50% is used. Green tea extract, cellulose and MG-stearate is capsuled in gelatin capsules. The capsules are produced by Dr. Loges, Hamburg. The green tea capsules are identical to the ones used in a multicenter trial to study the effect of green tea extract on the development of colon adenomas (see also ethics approval 68/09).

One capsule contains 150mg EGCG and around 300mg total catechins

Great importance is attached to the fact that the complete medication is from a single batch. The substance is stored under try (<60% humidity) and light-protected conditions at a room temperature of 25°C.

The drug intake at study day is supervised and documented with the exact time point. Drug should be taken with 200ml tab water without carbon dioxide.

6.3. Study Procedure

Day 1-4:
Participants get for four day in the morning and with a gap of 12h in the evening one capsule with 150mg EGCG respectively 300mg EGCG per day. They are asked to take the capsule with breakfast or dinner with one glass of tap water.

Study day:
At study day, participants appear in the morning under fasting conditions at the research ward. After a short physical check-up, a venous line is established and a blood sample for the basal level of kinetics is taken. The participants take one capsule with 150mg EGCG with one glass of tab water. In the following ten hours blood samples for the analysis of the EGCG plasma concentration is taken. After 4h and 10 h participant get a meal and a snack. No other food is allowed during their stay on the research ward.

6.4 Schema:

Day	0.00	0:30	1.00	2:00	3:00	4:00	5:00		7:00	9:00	20:00
-x	**Preliminary check-up, blood sample: RNA, DNA, EDTA-Plasma**										
-4	**1Cps. à 150mg EGCG**	← →<br>Intake at home, participants have to document the intake and adverse event in a diary in order to increase compliance.									**1Cps. à 150mg EGCG**
-3	**1Cps. à 150mg EGCG**										**1Cps. à 150mg EGCG**
-2	**1Cps. à 150mg EGCG**										**1Cps. à 150mg EGCG**
-1	**1Cps. à 150mg EGCG**										**1Cps. à 150mg EGCG**
**1**	**BE-0 (Predose): 1 Cps. 150mg EGCG**	**BE-1: 10ml**	**BE-2: 10ml, UAW**	**BE-3: 40ml BE: RNA DNA, Plasma, CRP**	**BE-4: 10ml, UAW**	**BE-5: 10ml, UAW, Lunch**	**BE-6: 10ml, UAW**		**BE-7: 10ml, UAW**	**BE-8: 10ml, UAW**	
Foo d	Fasten from 22:00 day -1	**	**	Small snack **after** BE-3		Lunch **after** BE-6			Snack	(Dinner **after** BE-9)	

BE: Blood sample for kinetic measurement; UAW: Adverse events; gray: Time on test station, HF: Heart rate; RR: blood pressure

**** If the participants suffers from hypoglycemia, 10g of glucose or a EGCG-free soft drink is allowed (better no juice)**

6.5 Blood sampling during study

During preliminary check-up one fasting blood sample (participant should not eat at least 3h before) for the analysis of routine laboratory parameters: Blood cell count, creatinine, glucose, CRP, bilirubin, GOT, GPT, γGT. Another 10ml of blood is taken for genotyping and 30ml for RNA and protein extraction. In total 60ml blood is taken during preliminary check-up.

During study day blood samples will be taken over 10 h for the analysis of EGCG kinetics and in addition 30ml blood is taken after 2h for RNA and protein extraction and the analysis of CRP. For the pharmacokinetic analysis flowing scheme is planned: blood sampling (each 10ml, EDTA monovette) at time point 0 (before intake of capsule, predosage) and 30min, 1,2,3,4,5,6,8 and 10 h after intake. The exact time of blood sampling is documented in the case report form (CRF) in the case that real time point differs more than +/- 5 minutes (for 30 min sampling +/- 2 minutes) from the planned time point. Exact time points will be used for pharmacokinetic calculations.
In total 120ml blood is taken during study day.

6.6. Experimental Procedure

6.6.1 Genotyping

Blood sample for genotyping are frozen at -20°C within 24h after sampling. For DNA extraction blood samples are slowly defrosted at room temperature. DNA is extracted with standardized procedure (Qiagen DNA extraction kit). For genotyping validated methods are used (DNA-sequencing, Taqman, PCR-RFLP)

6.6.2 Genome-wide analysis

This study is not primarily designed for the analysis of genome-wide DNA-data. But it is possible under certain conditions (for example that the kinetic results strongly differ in 6 out of 100 participants) that a genome-wide analysis could make sense. Therefor participants were asked to consent in a genome-wide analysis. These analyses have only the aim to explain the nutrikinetic or effects of EGCG. (narrow definition of the aim)

6.6.3 Candidate gene analysis

Variants in genes, which probably influence nutrikinetic or the effect of EGCG according to literature or could at least be involved due to biological plausibility, are analyzed. In a first step we will analyze known functional variants as well as gene variants with common alleles or haplotypes. We will analyze genes like Catechol-O-Methyltransferase (COMT), OATP1A2, -1B3 and other relevant transport proteins. Further we will analyze genes coding for glucuronyltransferases, and if we see significant effects of EGCG on oxidative stress, inflammation or cardio-vascular parameters we may also analyze genes related to this effects.

To identify new polymorphisms in genes which are yet not well characterized like OATP1B3 we plan to systematically sequence the coding and bordering non-coding regions of the gene

including 5'- und 3'-regions in a sub population of our participants. If we can detect such mutations we will establish specific genotyping assays.

6.6.4 RNA-isolation and gene expression analysis

Total RNA is isolated from blood sample using standard methods (Ficoll cell separation, Qiagen RNeasy Mini Kit, Pax-Gen). It is planned to investigate if the expression of genes of the drug transport, metabolism, cell growth, generation or detoxification of reactive oxygen species is changed by a 4-day treatment with EGCG. Expression of candidate genes is quantified using qPCR (Sybr Green, TaqMan). In this respect, as for the genetic analysis, participants were asked to consent in a genome-wide analysis. In this case common Affymetrix Expressions-Microarrays should be used. In addition to RNA, gene expression differences will also be semi-quantitatively on protein level using western blot. For this reason protein fractions are isolated from isolated lymphocytes and frozen at -80°C.

6.6.5. Bioanalytics

For the determination of EGCG concentration in the blood, 10ml EDTA blood samples are collected, protected from light and centrifuged within 30min after collection for 10 min and 2000g at room temperature. The supernatant (plasma) is divided into 500µl aliquots and 500µl stabilization buffer is added. Stabilization buffer contains EDTA and ascorbic acid. [100 mg ethylenediamin-tetraacidic acid (EDTA) disodiumsalt and 10 g ascorbic acid solved in 1000 mL sodium acetate buffer (35 mM: 2.38 g sodium acetate trihydrate and 1.00 mL acetic acid dissolved in water and adjusted to 1000 mL)]. Stabilized plasma samples are stored at -80°C and later shipped on try ice.

The analytic measurements will be carried out using HPLC and electro-chemical detection at the department of clinical pharmacology at the university Göttingen (director: Prof. Dr. med. J. Brockmöller) [13]. It is planned to analyze plasma concentrations of EGCG as the major aim of the project. If possible the plasma concentration of methylated metabolites (COMT), as well as glucuronides and sulfate metabolites of EGCG should be determined in the plasma samples or urine in order to characterize the causes of this variability.

6.7. Allowance and follow-up

Follow-up includes a check-up similar to the Preliminary check-up and performed after the study day. Participants get an allowance of 80€.

7. Statistical analysis

7.1. General

It is the aim of this study to investigate in vivo the influence of genetic polymorphisms in COMT, OATP1A2, OATP1B3 and further molecules involved in EGCG effect on the kinetics of EGCG. It should be tested if plasma-concentration-time profiles (AUC, Cmax, and oral clearance) differ in relation to the genotype.

.

7.2 Primary outcome

Oral clearance (Dosage/AUC) of 150mg EGCG in carriers of genetic polymorphisms compared to wild type carriers.

7.3 Secondary outcome

Maxima blood concentration (C_{max}), Time of the maximal blood concentration (T_{max}), half life time ($T_{1/2}$), adverse events, changes in gene expression profiles, epigenetic changes in blood cells, concentration profile of the used capsules including description of rate and variability of absorption, distribution (alpha half-life) and terminal elimination (beta half-life) of the relevant green tea catechins as far as they are detectable.

7.4 Sample size estimation

With a sample size of n=100 it is possible to detect genetic variation with an allele frequency of 5% at least once with a level of significance of 5%.

Assuming a frequency of 20% of carriers of the methionine variant (heterozygote plus homozygote carrier) and an allele frequency of 11% for the COMT polymorphism an effect on the pharmacokinetics would be detectable in n=100 participants. It had been shown that a mean AUC of 1403 ng/ml h with a standard deviation of 632 can be detected after multiple dosing of 200mg EGCG in n=9 participants [8]. If n=20 participants carry either Val/Met or Met/Met genotypes and n=80 carry Val/Val genotype a difference of 30% and more in the oral clearance of EGCG could be detected with a power of 80% and a significance of 0.05 (sample size calculated with nQuery Advisor 4.0).

In addition, with the estimated sample size of n=100 it is possible to determine the distribution of nutrikinetic parameters of EGCG in healthy participants and give evidence if there are sub-populations showing a poor absorption or fast metabolism or a good absorption and a poor elimination of this substances.

7.5 Statistical methods:

The results will be analyzed using non-parametric Jonckheere Terpstra Trend Tests based one the estimation of a trend in the EGCG kinetics in carriers of wild type >heterozygote > homozygote.

Wild type and homozygote carriers will be compared with parametric t-test. This study is of exploratory nature. A significant association detected by testing individual factors is suggested to be important, even if it is no longer significant after adjusting to multiple testing. This study is to our knowledge the first trial studying the genetic variability of green tea catechin EGCG and has the characteristics of a pilot study.

AUCs will be calculated using non-compartmental analysis as well as population kinetic modeling. An experimental analysis with calculation of median, mean, variance and graphic presentation in Box-Plots will be conducted.

Participants discontinuing the study or fulfilling exclusion criteria will be marked as drop-out and will be excluded from nutrikinetic analysis.

8. Safety – Risk for Participants

8.1 Study drug

For this study, a commercially available green tea extract with an EGCG content of 50% is used. Green tea extract is a nutrition supplement and no medical product. EGCG is the active substance of green tea extract. It is usually consumed as green tea or black tea beverage or it can be bought in drug stores or on the internet as nutrition supplement capsules (e.g. Dr. Loges Prävent capsules or BTC Vcaps).

8.2 Amount of drug

For this study 150mg of EGCG per capsule is twice daily used (300mg per day and participant). This is the amount which is usually the content of 3-5 cups of green tea every day.

8.3 Possible risks and adverse events

Adverse effects of green tea extract are described as rare events during long intake. No adverse effects have to be expected during 5 days of EGCG intake. Case reports reported a development of changes in the level of liver enzymes in the context of food or food supplement containing green tea [14]. From 216 reports about adverse events in temporal relation to the intake of food containing green tea, only in 7 cases a causal relation between increase of transaminases and the intake of green tea extract was plausible. The main compound EGCG seem to be causable as in experiments EGCG show a 10-time higher cytotoxicity on isolated rat liver cells than other catechins [15].

The dosage of 300mg EGCG used in this study which is equal to 4-7 cups of green tea every day is not associated with any known adverse event.

In a dose-finding study for EGCG 33 patients with stable remitted CLL were treated over 6 month with 400 and 2000mg daily. In this study grade 3 toxicity was observed in 2 Patients, in one case from diarrhea in the other case from abdominal pain [16].

8.4 Blood sampling

The risks of venous blood sampling are the occurrence of mild pain, bleeding or hematomas. Extremely rare but in principle possible are infection complications at the puncture, nerve injury or strong bleeding due to arterial puncture. These complications are very rare and in our practical experience such serious complications never happened in more than 1000 cases of venous blood sampling.

In principle, allergic reactions can occur due to all used materials, irritation caused by adhesive plaster is relative common. A disposition to allergic reaction will be inquired and considered if necessary.

8.5 Physical activity during study day

During study day the participants are free to move, but they should sit or lay down during blood sampling.

8.6 Eating and drinking during study.

One day before the study day participants should not drink tea, especially green tea. A list containing information about food which could contain EGCG or similar compounds is given to the participants ahead of study.

Participants must not eat from 22:00 the day before the study day until two hours after the intake of the study drug at study day. Afterward the participants get a bread roll, lunch and dinner during their stay on the research ward at study day. For the choice of the dish, attention will be payed that the food does not contain EGCG or similar substances. For drink participants get mineral water.

Because it is known that nutrikinetic of EGCG is influenced by food intake (more than 3-times higher blood levels under fastened then fed conditions) participants should not eat additional food. No more than 3L of water is allowed to be drunk during study day
.

8.7 Monitoring of the participants

To minimize the risks during study participants are continuously monitored by trained medical personal. Physicians are available at the research institute during the complete duration of study. All personal involved in the study is specifically trained at begin of the study.

9. Adverse Events

9.1 Definitions

An **adverse event** (**AE**) is any untoward medical occurrence in clinical investigation subject and which does not necessarily have a causal relationship with this treatment.
It includes every untoward medical occurrence in a patient or clinical investigation subject administered a pharmaceutical product or in our case a food supplement between first intake and 24hours after the last administration.
An adverse event (AE) can therefore be any unfavorable and unintended sign (including an abnormal laboratory finding), symptom, or disease temporally associated with the use of a medicinal (investigational) product, whether or not related to the medicinal (investigational) product

Preexisting Diseases which worsen during the study are also defined as AE. AEs can become serious adverse events if they fulfill following criteria.

A **serious adverse event (SAE)** is defined as each adverse event or side effect that independently from causality occurs between first administration and 24hours after the last administration and fulfills following criteria:

- results in death (remark: death is the consequence and not the event)
- is life-threatening, (remark: the term “life- threatening” describes an situation in which a patient is in danger to life, and not an event which could lead to death if it would have been more serious)
- requires inpatient hospitalization or prolongation of existing hospitalization,
- results in persistent or significant disability/incapacity, or
- is a congenital anomaly/birth defect.
- Event which leads to an action to prevent one of the above mentioned criteria.

An **adverse drug reaction** is defined as any adverse event (as defined above) for which a causality with the study is possible, likely or certain.

A **serious adverse drug reaction** is defined as any severe adverse event (as defined above) for which a causality with the study is possible, likely or certain.

An **unexpected serious adverse drug reaction** is a severe adverse event that is not a typical adverse event for the test drug or intervention documented in the information sheet for trial participants.

9.2. Handling and documentation of adverse events

All serious adverse events occurring during the trial are documented in the CRF. The severity or intensity of the adverse event is rated with a 4-point scale (mild, medium, severe, life-threatening) and documented in the CRF.

- Mild: events, not influencing daily life
- Medium: disorders that curb or impair normal daily activity
- Severe: inability to work or execute normal daily activity
- life-threatening: direct danger to life

The connection between treatment and adverse event has to be assessed.

Etic commission is informed as fast as possible about all serious and unexpected serious adverse drug reaction (as defined in 9.1) occurring during the study.

9.3 Handling and follow-up of adverse events.

All adverse events especially in causality with the test drug have to be followed-up until all symptoms completely fade away, the basal level is reached or the condition is stabilized. If there is a distinct explanation for the AE, it has to be documented in CRF.

10. Data management

10.1 Data collection, data analysis and data transfer

All data collected und this study is under data protection. Personal data with the exception to day of birth, age and gender will not be released from test facility. Only pseudonymized data is transferred in form of CRFs and stored for analysis
Only the investigators at the test facility are authorized to assign the pseudonymization code with the personal data of the participant. Upon end of the trial study documents are archived according to the actual rules of data protection law.

Study data is documented in case report forms (CRFs). Following documents are available for the study:

- documentation of informed consent
- screening
- inclusion and exclusion criteria
- study protocol
- completion of study
- documentation of AE

DNA/RNA and blood samples are pseudonymized with a study code (EGCG_Kin) and a number for the participant. Samples are stored as long as storage is possible and as long as a scientific use in the purpose of the study is possible. Samples are stored for such a long period of time because many targets (e.g. in Genome, Transcriptome, Proteome, Metabolome) will be analyzed for the project but analysis of this targets is time consuming and can last for many years. Participants are explicitly informed and have consented on the unlimited storage of samples at the University of Ulm and the transfer of plasma and urine samples to the university Göttingen. If a participant asks for the premature destruction of the samples after given consent, the participant should contact the clinical study investigator. Using an identification list the samples can be assigned to the participant and afterward be destroyed. The participants are explicitly informed about this fact in the participant information sheet. Samples are only used to study kinetics and the effects of EGCG.

10.2. Pseudonymization of the data and professional discretion

Persons with access to the data are informed about privacy and confirm with a signature the information on professional discretion. A transfer of data to other persons is forbidden.

11. Responsibilities and Signature

Principle investigator, study execution and analysis:
Prof. Dr. med. Julia Kirchheiner
Institut für Naturheilkunde und Klinische Pharmakologie
Universität Ulm
Helmholtzstr. 20
D-89081 Ulm
Tel: 0731-65603
Fax: 0731-65605
E-mail: julia.kirchheiner@uni-ulm.de

Pharmakogenetics/ Biomarker Screen:
Dr. med. Angela Seeringer
Sektion Klinische Pharmakologie
Institut für Naturheilkunde und Klinische Pharmakologie
Universität Ulm
Helmholtzstr. 20
89081 Ulm
Tel: 0731-65612
Fax: 0731-65605

Sample analytics, measurement of EGCG in plasma, pharmacokinetic analysis
Prof. Dr. med. Jürgen Brockmöller
Abteilung Klinische Pharmakologie der Universität Göttingen
Robert-Koch-Str. 40
D-37075 Göttingen, Germany
Telephone: +49 - 551 - 395770
Fax: +49 - 551 - 3912767
E-mail: jbrockm@gwdg.de

Financial Support:
German cancer aid

Ethics committee:

Ethiss committee of the medical faculty of the university Ulm

Prof. Dr. med. Uwe Brückner, head of ethics committee

Signature Principle investigator :

Date:<br><br><br><br>Prof. Dr. J. Stingl<br>(Name)

12. Literatur

1. Lee, M.J., et al. (2002). Pharmacokinetics of tea catechins after ingestion of green tea and (-)-epigallocatechin-3-gallate by humans: formation of different metabolites and individual variability. *Cancer Epidemiol Biomarkers Prev*. 11(10 Pt 1): p. 1025-32.
2. Yang, C.S., et al. (1998). Blood and urine levels of tea catechins after ingestion of different amounts of green tea by human volunteers. *Cancer Epidemiol Biomarkers Prev*. 7(4): p. 351-4.
3. Roth, M., B. Timmermann, and B. Hagenbuch (2011). Interactions of green tea catechins with organic anion transporting polypeptides. *Drug Metab Dispos*. epub ahead of print.
4. Jodoin, J., M. Demeule, and R. Beliveau (2002). Inhibition of the multidrug resistance P-glycoprotein activity by green tea polyphenols. *Biochim Biophys Acta*. 1542(1-3): p. 149-59.
5. Zhu, B.T., et al. (2000). O-Methylation of tea polyphenols catalyzed by human placental cytosolic catechol-O-methyltransferase. *Drug Metab Dispos*. 28(9): p. 1024-30.
6. Chow, H.H., et al. (2001). Phase I pharmacokinetic study of tea polyphenols following single-dose administration of epigallocatechin gallate and polyphenon E. *Cancer Epidemiol Biomarkers Prev*. 10(1): p. 53-8.
7. Chow, H.H., et al. (2003). Pharmacokinetics and safety of green tea polyphenols after multiple-dose administration of epigallocatechin gallate and polyphenon E in healthy individuals. *Clin Cancer Res*. 9(9): p. 3312-9.
8. Ullmann, U., et al. (2004). Plasma-kinetic characteristics of purified and isolated green tea catechin epigallocatechin gallate (EGCG) after 10 days repeated dosing in healthy volunteers. *Int J Vitam Nutr Res*. 74(4): p. 269-78.
9. Ullmann, U., et al. (2003). A single ascending dose study of epigallocatechin gallate in healthy volunteers. *J Int Med Res*. 31(2): p. 88-101.
10. Lachman, H.M. (2008). Does COMT val158met affect behavioral phenotypes: yes, no, maybe? *Neuropsychopharmacology*. 33(13): p. 3027-9.
11. Franke, R.M., L.A. Scherkenbach, and A. Sparreboom (2009). Pharmacogenetics of the organic anion transporting polypeptide 1A2. *Pharmacogenomics*. 10(3): p. 339-44.
12. Schwarz, U.I., et al. Identification of novel functional organic anion-transporting polypeptide 1B3 polymorphisms and assessment of substrate specificity. *Pharmacogenet Genomics*. 21(3): p. 103-14.
13. Lee, M.J., et al. (1995). Analysis of plasma and urinary tea polyphenols in human subjects. *Cancer Epidemiol Biomarkers Prev*. 4(4): p. 393-9.
14. Sarma, D.N., et al. (2008). Safety of green tea extracts : a systematic review by the US Pharmacopeia. *Drug Saf*. 31(6): p. 469-84.
15. Galati, G., et al. (2006). Cellular and in vivo hepatotoxicity caused by green tea phenolic acids and catechins. *Free Radic Biol Med*. 40(4): p. 570-80.
16. Shanafelt, T.D., et al. (2009). Phase I trial of daily oral Polyphenon E in patients with asymptomatic Rai stage 0 to II chronic lymphocytic leukemia. *J Clin Oncol*. 27(23): p. 3808-14.
17. Chow, H.H., et al. (2005). Effects of dosing condition on the oral bioavailability of green tea catechins after single-dose administration of Polyphenon E in healthy individuals. *Clin Cancer Res*. 11(12): p. 4627-33.
