# Supplementary material for: Population nutrikinetics of green tea extract
Source: PLoS One. 2018 Feb 21;13(2):e0193074. doi: 10.1371/journal.pone.0193074 (PMC5821365; doi:10.1371/journal.pone.0193074)
Supplement: S5 File — (PDF) [file pone.0193074.s006.pdf]

Research Article

Population nutrikinetics of green tea extract

Catharina Scholl[1*], Anna Lepper[2], Thorsten Lehr[3], Nina Hanke[3], Katharina Luise Schneider[1], Jürgen Brockmöller[4], Thomas Seufferlein[5], Julia Carolin Stingl[1]

Modeling Supplement

***** Corresponding author
Email: catharina.scholl@bfarm-research.de

1. Non-compartmental analysis

Non-compartmental analysis using Phoenix WinNonlin (Version 6.4) was conducted to calculate the following output parameters: C_{max}, t_{max}, $AUC_{0\text{-}end}$, $t_{1/2}$, V/F, CL/F. Their computation formulas are given below.

C_{max}: Maximum observed concentration, occurring at t_{max}. If not unique, then the first maximum is used.

t_{max}: Time of maximum observed concentration. For steady-state data, t_{max} corresponds to points collected during a dosing interval. If the maximum observed concentration is not unique, then the first maximum is used.

$AUC_{0\text{-}end}$: Area under the curve from the time of dosing (t_0) to the last measurable (positive) concentration (t_{end}). Calculated with the linear trapezoidal rule (where δt is (t_2-t_1))

$$AUC(t1 - t2) \ = \ \delta t \ \cdot \ \frac{C1 + C2}{2}$$

and the linear interpolation method (to find C* at time t* for $t_1 < t^* < t_2$)

$$C * = \ C1 \ + \left|\frac{t * \ - t1}{t2 \ - \ t1}\right| \cdot \ (C2 \ - \ C1)$$

$t_{1/2}$: Terminal half-life was estimated as $t_{1/2}$ = ln(2)/ λz. λz is the first order rate constant associated with the terminal (log-linear) portion of the plasma concentration curve. Estimated by linear regression of time vs. log concentration.

V/F: For oral administration models, the fraction of dose absorbed cannot be estimated; therefore Volume for these models is V/F where F is the fraction of dose absorbed. Volume of distribution based on the terminal phase and the AUC starting at the time of dosing (t_0) extrapolated from the last measurable concentration (C_{end}) to infinity was estimated as

$$V/F \ = \ \frac{Dose}{\lambda z \ \cdot \ (AUC(0 - end) \ + \ \frac{Cend}{\lambda z})} \ / \ F$$

CL/F: For oral administration models, the fraction of dose absorbed cannot be estimated; therefore Clearance for these models is CL/F where F is the fraction of dose absorbed. Total body clearance at steady-state was estimated as (where τ is the dosing interval)

$$CL/F \ = \ \frac{Dose}{AUC(0 - \tau)} \ / \ F$$

2. Compartmental pharmacokinetic analysis

Concentration-time profiles of EGCG, EGC and ECG were best described by two-compartment models with one central and one peripheral compartment. Absorption of the gallate compounds EGCG and ECG was modeled by consecutive zero-order dissolution and first-order absorption with lag time. Absorption of EGC was modeled by first-order absorption with lag time, only. The final 3 model scripts are given below.

```
$PROBLEM        Green Tea Extract EGCG
$INPUT          ID TIME TAD AMT RATE DOSE EVID CMT DV ANALYTE SEX AGE WT HT BMI COMT MRP22T
                MRP37T MRP71T MRP81T OATP41T OATP23T OATP11T PGP10T PGP11T PGP20T SULT18T SULT37T SULT75T
                UGT GREENTEA SMOKER ALCOHOL CONTRACEPTIVES AST ALT BILI LEUKO ERY HB HK MCV MCH MCHC
                THROMBO
$DATA           ../DATASET/Bfarm_Tea_PK_V13.dat IGNORE=@ IGNORE(ANALYTE.NE.1)
$SUBROUTINES    ADVAN6 TOL=5

$MODEL
COMP=(DEPOT)
COMP=(CENTRAL, DEFOBS)
COMP=(PERIPH)

$PK
OATP41EFF=THETA(9)                                ; OATP1B1_rs4149056 →   OATP41T: TT=0, CT=1, CC=2
MRP71EFF=1
IF (MRP71T.EQ.0) MRP71EFF=THETA(10)               ; MRP2_rs717620 →       MRP71T: CC=0, CT=1, TT=2
K12=THETA(1)*EXP(ETA(1))                          ; K12 depot to central
CL=THETA(2)*(1+OATP41EFF*OATP41T)*EXP(ETA(2))     ; CL elimination from central
V2=THETA(3)                                       ; V2 central volume
Q=THETA(4)                                        ; Q central to peripheral
V3=THETA(5)                                       ; V3 peripheral volume
D1=THETA(6)*EXP(ETA(3))                           ; Duration
F1=THETA(7)*MRP71EFF*EXP(ETA(4))                  ; Bioavailability from depot
ALAG=THETA(8)*EXP(ETA(5))                         ; Lag time
S2=V2/1000
K20=CL/V2
K23=Q/V2
K32=Q/V3

$DES
DADT(1)=        - K12*A(1)
DADT(2)=        + K12*A(1)      - K23*A(2) + K32*A(3)       - K20*A(2)
DADT(3)=                        + K23*A(2) - K32*A(3)

$ERROR
IPRED=F
DEL=0
IF (IPRED.EQ.0) DEL=0.0001
W=IPRED
IRES=DV-IPRED
IWRES=IRES/(W+DEL)
Y=IPRED*(1+EPS(1))+EPS(2)

$THETA (0, 1)           ; K12
$THETA (0, 200)         ; CL
$THETA (0, 300)         ; V2
$THETA (0, 100)         ; Q
$THETA (0, 3000)        ; V3
$THETA (0, 1)           ; D
$THETA (1 FIX)          ; F
$THETA (0, 0.5)         ; ALAG
$THETA (1)              ; OATP41EFF additive
$THETA (1)              ; MRP71EFF CC

$OMEGA 0.1              ; K12
$OMEGA 0.1              ; CL
$OMEGA 0.1              ; D1
$OMEGA 0.1              ; F1
$OMEGA 0.1              ; ALAG

$SIGMA 0.1              ; RV prop.
$SIGMA 0.1              ; RV add.

$EST METHOD=1 INTER MAXEVAL=9999 NOABORT PRINT=1 SIG=3 POSTHOC
$COV
$TABLE ID TIME IPRED IWRES CWRES TAD AMT RATE DOSE EVID CMT ANALYTE OATP41EFF MRP71EFF ETAS(1:LAST)
FILE=tab2000 NOPRINT ONEHEADER
```

```
$PROBLEM        Green Tea Extract EGC
$INPUT          ID TIME TAD AMT RATE=DROP DOSE EVID CMT DV ANALYTE SEX AGE WT HT BMI COMT MRP22T MRP37T
                MRP71T MRP81T OATP41T OATP23T OATP11T PGP10T PGP11T PGP20T SULT18T SULT37T SULT75T UGT
                GREENTEA SMOKER ALCOHOL CONTRACEPTIVES AST ALT BILI LEUKO ERY HB HK MCV MCH MCHC
                THROMBO
$DATA           ../DATASET/Bfarm_Tea_PK_V13.dat IGNORE=@ IGNORE(ANALYTE.NE.2)
$SUBROUTINES    ADVAN6 TOL=5

$MODEL
COMP=(DEPOT)
COMP=(CENTRAL, DEFOBS)
COMP=(PERIPH)

$PK
CONCEP=1
IF (CONTRACEPTIVES.EQ.1) CONCEP=THETA(8)              ; Use of hormonal contraceptives → No=0, Yes=1
OATP23EFF=1                                           ;
IF (OATP23T.EQ.0) OATP23EFF=THETA(9)                  ; OATP1B1_rs2306283 →      OATP23T: AA=0, GA=1, GG=2
MRP37EFF=1                                            ;
IF (MRP37T.EQ.0) MRP37EFF=THETA(10)                   ; MRP2_rs3740066 →         MRP37T: CC=0, CT=1, TT=2
MRP71EFF=THETA(11)                                    ; MRP2_rs717620 →          MRP71T: CC=0, CT=1, TT=2
UGTEFF=1                                              ;
IF (UGT.EQ.0) UGTEFF=THETA(12)                        ; UGT1A1*28_[6TA/7TA] →    UGT: (-)=0, (-/TA)=1, TA=2
COMTEFF=1                                             ;
IF (COMT.EQ.2) COMTEFF=THETA(13)                      ; COMT_rs4680 →            COMT: GG=0, GA=1, AA=2
K12=THETA(1)                                          ; K12 depot to central
CL=THETA(2)*OATP23EFF*UGTEFF*EXP(ETA(1))              ; CL elimination from central
V2=THETA(3)*CONCEP*(1+MRP71EFF*MRP71T)*EXP(ETA(2))    ; V2 central volume
Q=THETA(4)*MRP37EFF*EXP(ETA(3))                       ; Q central to peripheral
V3=THETA(5)                                           ; V3 peripheral volume
F1=THETA(6)*COMTEFF*EXP(ETA(4))                       ; Bioavailability from depot
ALAG=THETA(7)*EXP(ETA(5))                             ; Lag time
S2=V2/1000
K20=CL/V2
K23=Q/V2
K32=Q/V3

$DES
DADT(1)=    - K12*A(1)
DADT(2)=    + K12*A(1)     - K23*A(2) + K32*A(3)     - K20*A(2)
DADT(3)=                   + K23*A(2) - K32*A(3)

$ERROR
IPRED=F
DEL=0
IF (IPRED.EQ.0) DEL=0.0001
W=IPRED
IRES=DV-IPRED
IWRES=IRES/(W+DEL)
Y=IPRED*(1+EPS(1))

$THETA (0, 1)           ; K12
$THETA (0, 600)         ; CL
$THETA (0, 1000)        ; V2
$THETA (0, 700)         ; Q
$THETA (0, 9000)        ; V3
$THETA (1 FIX)          ; F
$THETA (0, 0.4)         ; ALAG
$THETA (1)              ; CONCEP
$THETA (0.5)            ; OATP23EFF AA
$THETA (2)              ; MRP37EFF CC
$THETA (-0.4)           ; MRP71EFF additive
$THETA (1)              ; UGTEFF (-)
$THETA (1)              ; COMTEFF AA

$OMEGA 0.2              ; CL
$OMEGA 0.7              ; V2
$OMEGA 1.1              ; Q
$OMEGA 0.1              ; F
$OMEGA 0.01             ; ALAG

$SIGMA 0.1              ; RV prop.

$EST METHOD=1 INTER MAXEVAL=9999 NOABORT PRINT=1 SIG=3 POSTHOC
$COV
$TABLE ID TIME IPRED IWRES CWRES TAD AMT DOSE EVID CMT ANALYTE CONCEP OATP23EFF MRP37EFF MRP71EFF UGTEFF
COMTEFF ETAS(1:LAST) FILE=tab2033 NOPRINT ONEHEADER
```

```
$PROBLEM        Green Tea Extract ECG
$INPUT          ID TIME TAD AMT RATE=DROP DOSE EVID CMT DV ANALYTE SEX AGE WT HT BMI COMT MRP22T MRP37T
                MRP71T MRP81T OATP41T OATP23T OATP11T PGP10T PGP11T PGP20T SULT18T SULT37T SULT75T UGT
                GREENTEA SMOKER ALCOHOL CONTRACEPTIVES AST ALT BILI LEUKO ERY HB HK MCV MCH MCHC
                THROMBO
$DATA           ../DATASET/Bfarm_Tea_PK_V13.dat IGNORE=@ IGNORE(ANALYTE.NE.3)
$SUBROUTINES    ADVAN6 TOL=5

$MODEL
COMP=(DEPOT)
COMP=(CENTRAL, DEFOBS)
COMP=(PERIPH)

$PK
K12=THETA(1)*EXP(ETA(1))          ; K12 depot to central
CL=THETA(2)*EXP(ETA(2))           ; CL elimination from central
V2=THETA(3)                       ; V2 central volume
Q=THETA(4)                        ; Q central to peripheral
V3=THETA(5)                       ; V3 peripheral volume
D1=THETA(6)*EXP(ETA(3))           ; Duration
F1=THETA(7)*EXP(ETA(4))           ; Bioavailability from depot
ALAG=THETA(8)*EXP(ETA(5))         ; Lag time
S2=V2/1000
K20=CL/V2
K23=Q/V2
K32=Q/V3

$DES
DADT(1)=        - K12*A(1)
DADT(2)=        + K12*A(1)        - K23*A(2) + K32*A(3)        - K20*A(2)
DADT(3)=                          + K23*A(2) - K32*A(3)

$ERROR
IPRED=F
DEL=0
IF (IPRED.EQ.0) DEL=0.0001
W=IPRED
IRES=DV-IPRED
IWRES=IRES/(W+DEL)
Y=IPRED*(1+EPS(1))+EPS(2)

$THETA (0, 2)                     ; K12
$THETA (0, 200)                   ; CL
$THETA (0, 300)                   ; V2
$THETA (0, 100)                   ; Q
$THETA (0, 3000)                  ; V3
$THETA (0, 2)                     ; D
$THETA (1 FIX)                    ; F
$THETA (0, 0.5)                   ; ALAG

$OMEGA BLOCK (5)
0.571                             ; K12
-0.11 0.137                       ; CL
0.21 -0.0994 0.619                ; D
-0.0044 0.0861 -0.0146 0.32       ; F
0.0069 0.0316 0.0572 0.0602 0.186 ; ALAG

$SIGMA 0.1                        ; RV prop.
$SIGMA 1                          ; RV add.

$EST METHOD=1 INTER MAXEVAL=9999 NOABORT PRINT=1 SIG=3 POSTHOC
$COV
$TABLE ID TIME IPRED IWRES CWRES TAD AMT RATE DOSE EVID CMT ANALYTE OATP41EFF MRP71EFF ETAS(1:LAST)
FILE=tab701 NOPRINT ONEHEADER
```

3. Model evaluation plots

Standard VPCs and logarithmic goodness-of-fit plots are presented in the main document (Figures 6 and 7). Linear goodness-of-fit plots and plots of conditional weighted residuals versus predicted plasma concentrations as well as versus time after dose are given below.

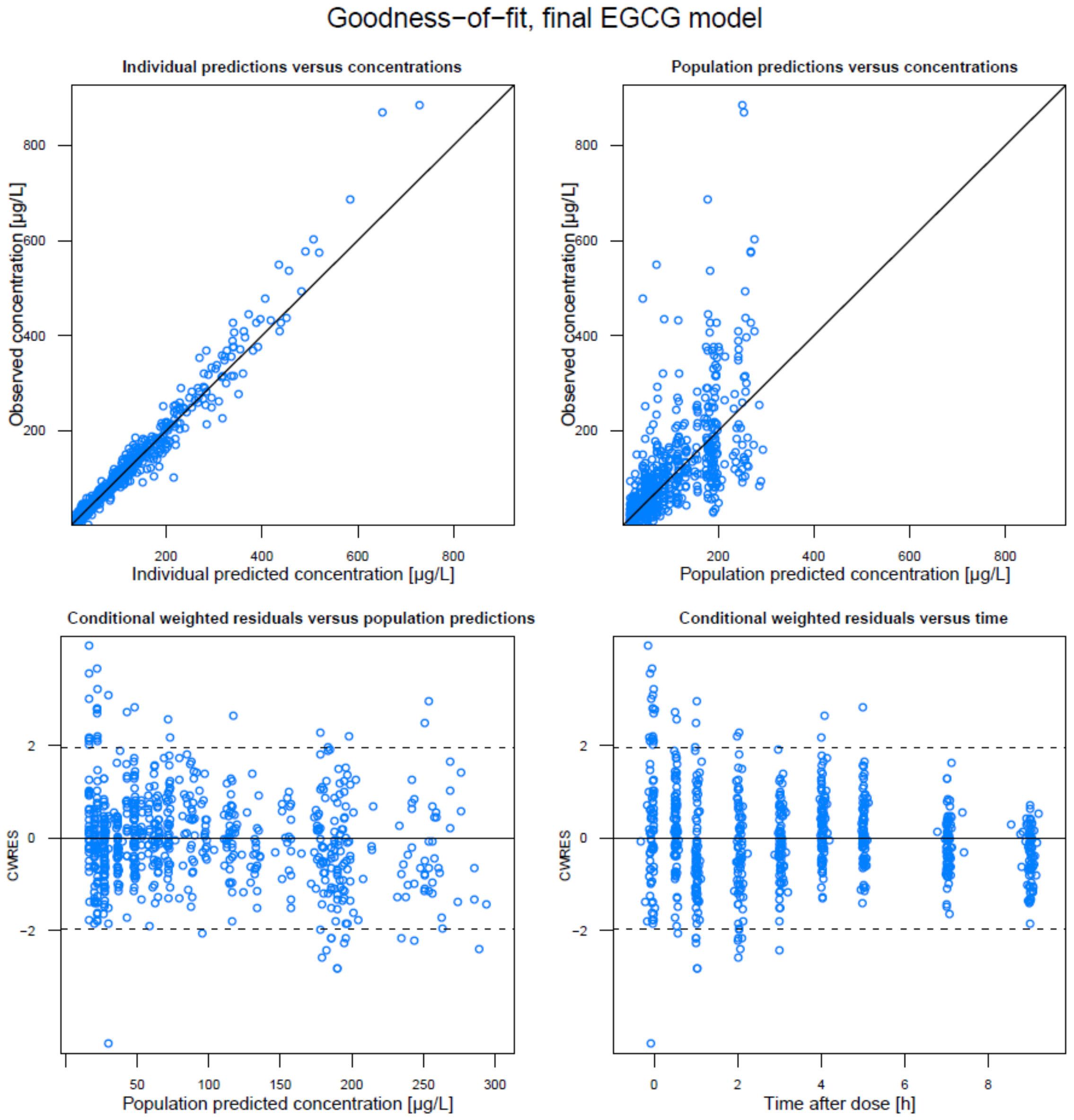

Fig 1: Goodness-of-fit plots and residual plots of the final EGCG PopPK model. The upper panel shows observed (y-axis) vs. predicted plasma concentrations (x-axis) scattered around the line of identity. The lower panel shows conditional weighted residuals (y-axis) vs. predicted plasma concentrations and vs. time (x-axis) scattered around a mean of 0.

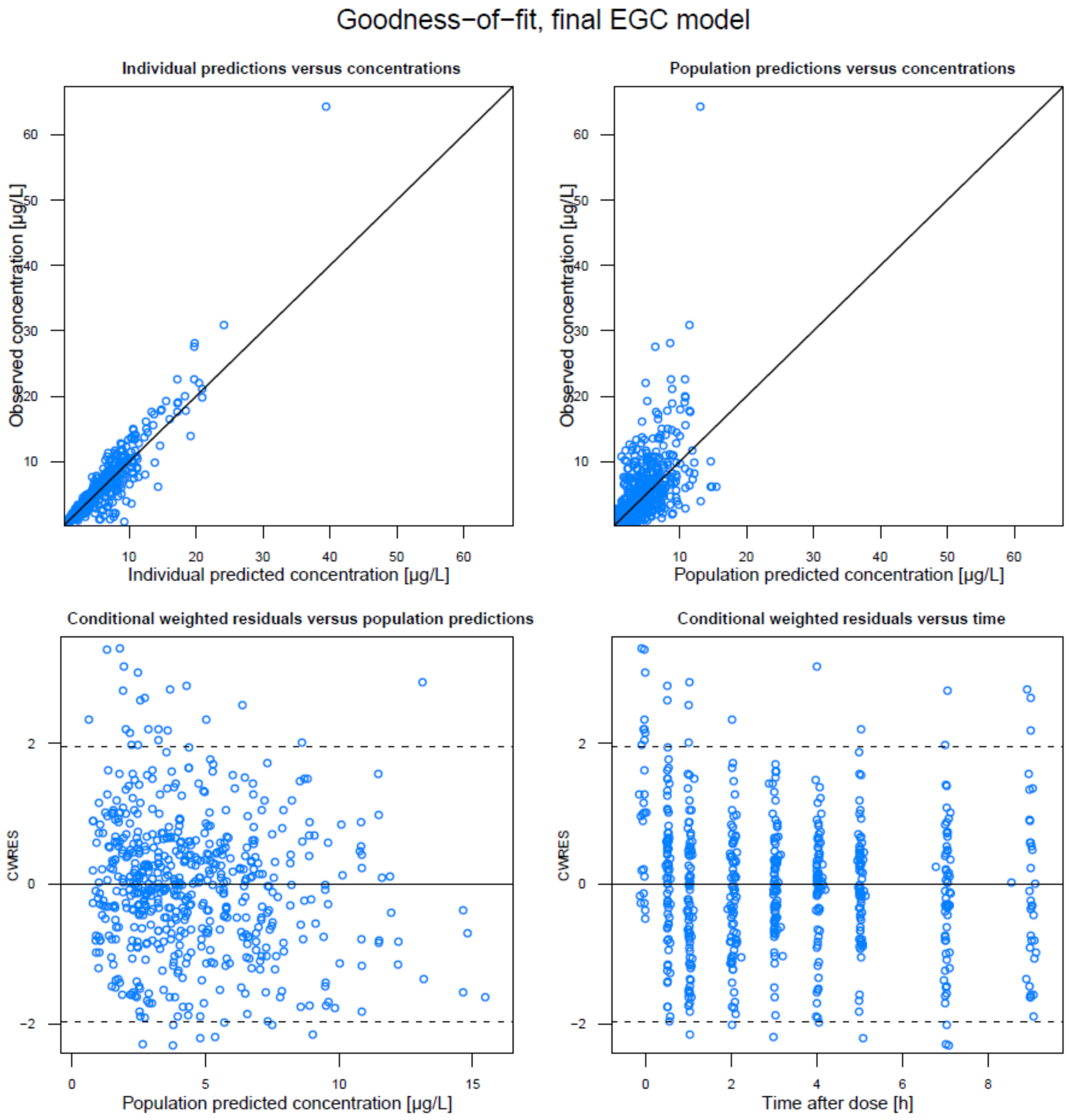

Fig 2: Goodness-of-fit plots and residual plots of the final EGC PopPK model. The upper panel shows observed (y-axis) vs. predicted plasma concentrations (x-axis) scattered around the line of identity. The lower panel shows conditional weighted residuals (y-axis) vs. predicted plasma concentrations and vs. time (x-axis) scattered around a mean of 0.

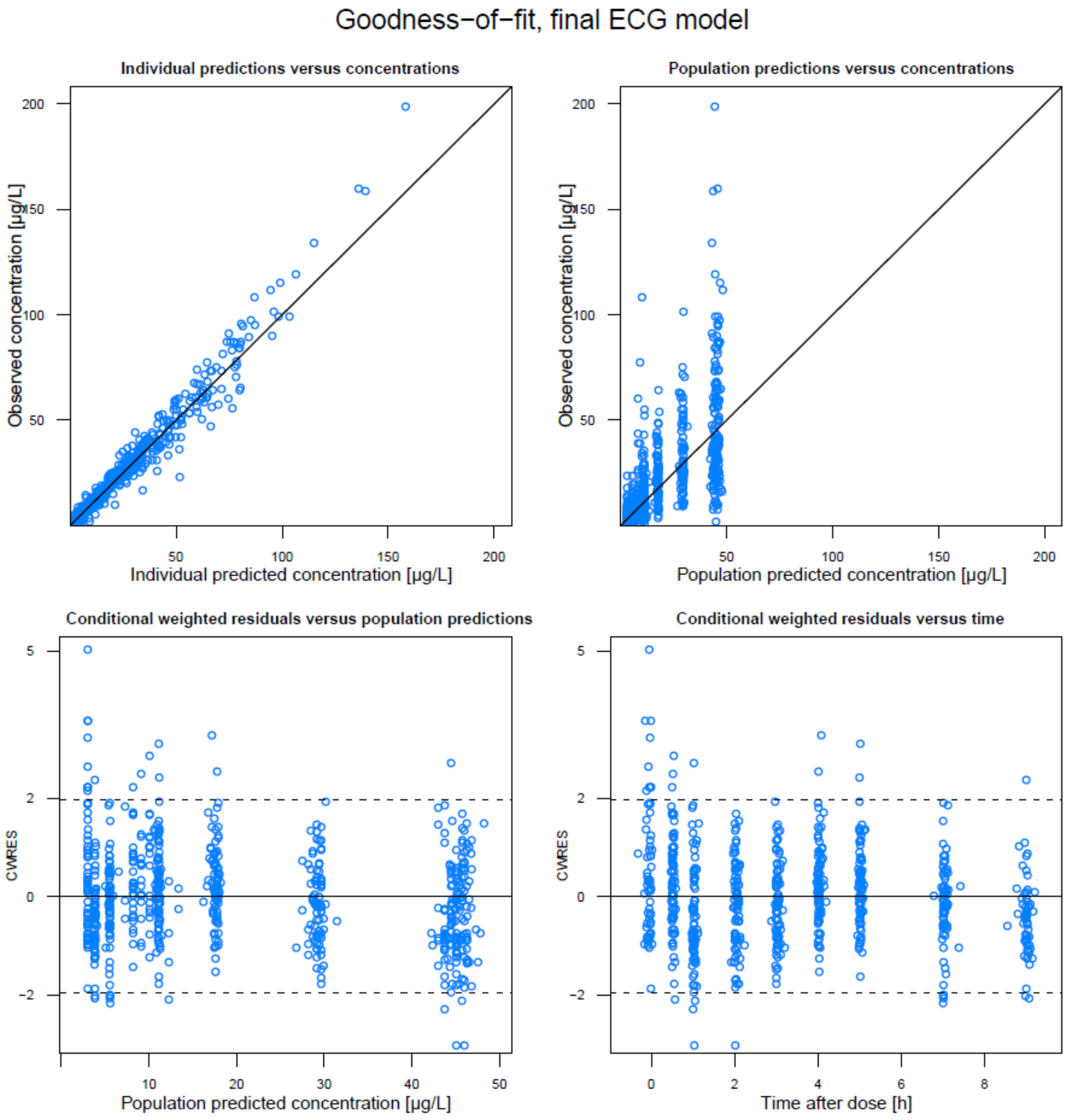

Fig 3: Goodness-of-fit plots and residual plots of the final ECG PopPK model. The upper panel shows observed (y-axis) vs. predicted plasma concentrations (x-axis) scattered around the line of identity. The lower panel shows conditional weighted residuals (y-axis) vs. predicted plasma concentrations and vs. time (x-axis) scattered around a mean of 0.

References:

Phoenix® WinNonlin® User's Guide (Phoenix WinNonlin 6.4), Certara, L.P., St. Louis, MO, USA
